# Genetic Interactions of *MAF1* Identify a Role for Med20 in Transcriptional Repression of Ribosomal Protein Genes

**DOI:** 10.1371/journal.pgen.1000112

**Published:** 2008-07-04

**Authors:** Ian M. Willis, Gordon Chua, Amy H. Tong, Renee L. Brost, Timothy R. Hughes, Charles Boone, Robyn D. Moir

**Affiliations:** 1 Department of Biochemistry, Albert Einstein College of Medicine, Bronx, New York, United States of America; 2 Banting and Best Department of Medical Research, University of Toronto, Toronto, Ontario, Canada; Yale University, United States of America

## Abstract

Transcriptional repression of ribosomal components and tRNAs is coordinately regulated in response to a wide variety of environmental stresses. Part of this response involves the convergence of different nutritional and stress signaling pathways on Maf1, a protein that is essential for repressing transcription by RNA polymerase (pol) III in *Saccharomyces cerevisiae*. Here we identify the functions buffering yeast cells that are unable to down-regulate transcription by RNA pol III. *MAF1* genetic interactions identified in screens of non-essential gene-deletions and conditionally expressed essential genes reveal a highly interconnected network of 64 genes involved in ribosome biogenesis, RNA pol II transcription, tRNA modification, ubiquitin-dependent proteolysis and other processes. A survey of non-essential *MAF1* synthetic sick/lethal (SSL) genes identified six gene-deletions that are defective in transcriptional repression of ribosomal protein (RP) genes following rapamycin treatment. This subset of *MAF1* SSL genes included *MED20* which encodes a head module subunit of the RNA pol II Mediator complex. Genetic interactions between *MAF1* and subunits in each structural module of Mediator were investigated to examine the functional relationship between these transcriptional regulators. Gene expression profiling identified a prominent and highly selective role for Med20 in the repression of RP gene transcription under multiple conditions. In addition, attenuated repression of RP genes by rapamycin was observed in a strain deleted for the Mediator tail module subunit Med16. The data suggest that Mediator and Maf1 function in parallel pathways to negatively regulate RP mRNA and tRNA synthesis.

## Author Summary

The Maf1 protein is an essential negative regulator of transcription by RNA polymerase III in *S. cerevisiae* and functions to integrate responses from diverse nutritional and stress signaling pathways that coordinately regulate ribosome and tRNA synthesis. These signaling pathways are not well-defined, and efforts to understand the role of Maf1 in this process have been complicated by a lack of known functional motifs in the protein and by a paucity of direct physical interactions with Maf1. To understand the biological importance of down-regulating RNA polymerase III transcription and to identify functional relationships with Maf1, we employed synthetic genetic array (SGA) analysis. We show that the genetic neighborhood around Maf1 is highly interconnected and enriched for a small number of functional categories, most of which are logically linked to the function of Maf1 as the regulator of RNA polymerase III transcription. We found that deletions in a subset of *MAF1* SSL genes, including subunits of the RNA polymerase II Mediator complex, lead to defects in transcriptional repression of ribosomal protein (RP) genes. Since Mediator subunits are not efficiently cross-linked to RP genes in chromatin, our results suggest that Mediator interactions with these highly expressed genes are fundamentally different from many other genes.

## Introduction

Nuclear gene transcription in proliferating cells is dedicated primarily to the synthesis of ribosomes and tRNAs. As illustrated by studies in *Saccharomyces cerevisiae*, the doubling of cell mass with each cell cycle involves the production of ∼200,000 ribosomes along with 3–6 million molecules of tRNA and consumes >80% of the nucleotides needed for transcription during this ∼100 minute interval [1]–[3]. This expenditure of metabolic energy is tightly regulated by diverse signaling pathways that sense the quality and quantity of nutrients or environmental stresses [1],[2]. Under conditions that are unfavorable for cell growth, transcription of rDNA and tRNA genes by RNA pols I and III and RNA pol II transcription of ribosomal protein (RP) genes is rapidly and coordinately repressed [1],[4]. Current evidence suggests that this coordinate response results from the convergence of specific signaling pathways on one or more transcription components in each polymerase system [4]–[9] and references therein. However, substantial gaps in understanding remain concerning the components and structure of these pathways, their targets and mechanisms of action.

Studies on RP gene transcription have identified several regulatory factors including Sfp1, Rap1, Fhl1, Ifh1 and Crf1 [5]–[7],[9] and references therein but it is unclear how these proteins communicate with the general RNA pol II transcription machinery. In contrast to this complexity, a single negative regulatory protein, Maf1, appears to serve as the conduit through which all repression signals pass in order to affect transcription by RNA pol III [4],[10]. The Maf1 protein interacts directly with Brf1, a subunit of the initiation factor TFIIIB, as well as RNA pol III and these interactions inhibit the assembly and function of TFIIIB-DNA complexes in vitro [10],[11]. The functional importance of these interactions is supported by their conservation from yeast to humans [12]. The essential role of Maf1 in the repression of RNA pol III transcription demonstrates a capacity to integrate responses from multiple nutritional and stress signaling pathways that coordinately regulate ribosome and tRNA synthesis [13]. This property of Maf1 provides unique opportunities to examine the mechanisms of signal integration, the nature of the upstream pathways, their downstream targets and their effects on the transcription machinery.

Yeast strains deleted for *MAF1* are viable and exhibit wild-type growth rates even though 10–15% of nuclear gene transcription is refractory to repression [2]. Maf1 does not contain any motifs of known function and evidence from a variety of sources suggests that the majority of Maf1 in yeast is not stably associated with other proteins under normal or repressing conditions: Co-immunoprecipitation experiments find only 10–20% of cellular Maf1 associated with RNA pol III and <1% of Maf1 associated with Brf1 [10],[11]. No other significant interactions have been found by affinity purification and mass spectrometry of protein complexes in yeast or in genome-wide two hybrid screens [14]. Given the limited physical interactions of Maf1, we initiated a study of its functional relationships using synthetic genetic array (SGA) analysis. The local genetic neighborhood around *MAF1* is highly interconnected and enriched for components of several protein complexes involved in ribosome biogenesis and RNA pol II transcription. We show that genetic interactions between *MAF1* and subunits of the RNA pol II Mediator complex, in particular *MED20*, are functionally linked by a common role in repression of tRNA and RP gene transcription, respectively.

## Results

### Synthetic Genetic Array Analysis of *MAF1*

**Figure 1:**
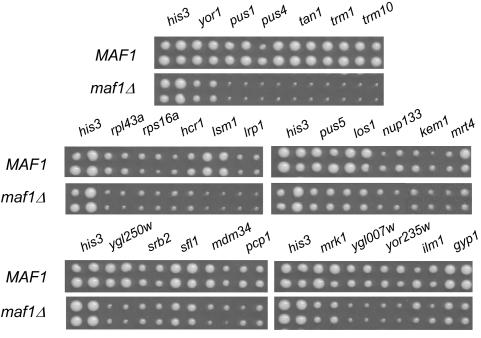
Genetic interactions between *MAF1* and non-essential gene deletions. Representative viable gene-deletion strains (G418-resistant) that were confirmed by random spore analysis as having fitness defects with *maf1Δ* were arrayed in quadruplicate and crossed to clonNat-resistant *MAF1* (Y5518) and *maf1Δ* (Y6338) query strains to compare the growth of haploid double-drug resistant strains following the standard SGA protocol [15]. The final double-drug containing plates were incubated at 30°C. A *his3Δ* strain was included as a negative control (i.e. no interaction with the *maf1Δ* strain).

A *maf1Δ* strain was screened in triplicate against an ordered array of ∼4700 viable gene-deletion strains and the relative growth of the double mutants was scored by computer-based image analysis [15]. Random spore analysis was then used to validate candidate genetic interactions. The initial list of *MAF1* SSL interactions contained 35 genes (Figure 1 and Table S1). Subsequently, the analysis was extended to an array of ∼800 strains containing different essential genes under tetracycline (Tet) promoter control [16]. Consistent with the ∼five-fold higher interaction density of essential genes in synthetic genetic networks [17], an additional 29 SSL interactions were validated by random spore analysis from triplicate screens of a *maf1Δ* query strain against the Tet-promoter array. The entire collection of 64 genes exhibiting synthetic interactions with *MAF1* is highly enriched for a small number of functional categories, several of which are logically linked to the function of Maf1 as a transcriptional regulator of RNA pol III genes. Notably, 40% of *MAF1* SSL genes (26/64 genes, *p*<7.0E-18) are involved in ribosome biogenesis or translation (Table S1). Other functional categories that are represented at significantly higher frequencies than expected by chance include RNA pol II transcription (9 genes, *p*<5.0E-4), tRNA modification (6 genes, *p*<4.0E-6) and ubiquitin-dependent proteolysis (5 genes, *p*<7.9E-3). These data suggest important functional relationships between *MAF1* and the genes within these categories [18].

**Figure 2:**
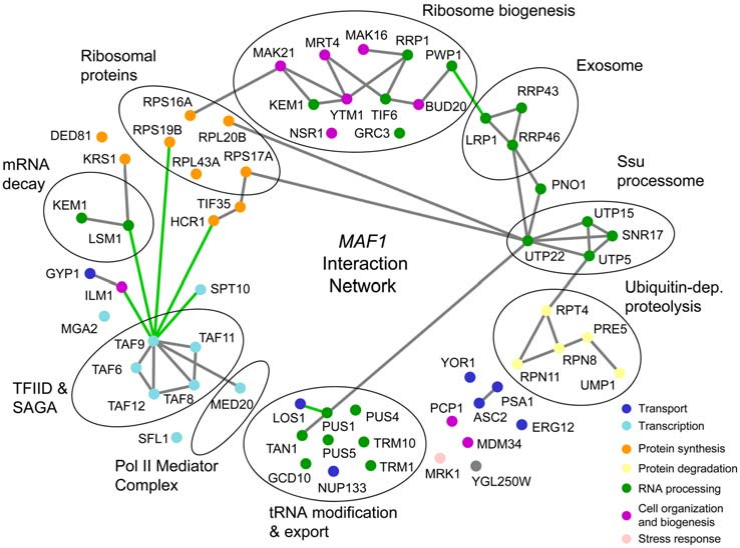
*MAF1* genetic interaction network. Genetic and physical interactions from BioGRID are shown between *MAF1* SSL genes identified in screens of the essential and non-essential strain arrays. Nodes are colored by Bioprocess. Circles identify well-defined protein complexes except for the tRNA modification & export group where the genes are related by biochemical function. Two *MAF1* SSL genes (*FYV5* and *YGL007W*) that lacked interactions in the BioGRID database are not shown in the figure. Genetic interactions are shown in green and protein-protein interactions determined by affinity purification are shown in gray.

To determine the relationships between the genes in the *MAF1* genetic interaction network, each SSL gene was queried against the BioGRID database [14] to compile a list of known genetic and physical interactions. These interactions were then superimposed on the set of *MAF1* SSL genes and the overlap was displayed graphically using Osprey software (Figure 2). The resulting interaction network is remarkably coherent; 70% (45/64) of *MAF1* SSL genes are connected by genetic or protein-protein interactions to one or more genes in the network. The majority of these interactions (47 gray edges out of 54 total interactions, Figure 2) were determined from multiple studies by affinity purification and mass spectrometry [14] and identify components of several well known macromolecular complexes (the 26S proteasome, the ssu processome, the exosome, pre-ribosomal processing intermediates, the cytoplasmic Lsm complex, the TFIID and SAGA complexes and the RNA pol II Mediator complex). The connectivity between these complexes suggests that a relatively small number of biological explanations could account for the ability of *MAF1* SSL genes to buffer cells that are unable to down-regulate RNA pol III transcription (see below and in the Discussion).

Within the broad functional category of ribosome biogenesis, defects in the synthesis of the large or small ribosomal subunits resulting from impaired rRNA processing, reduced levels of ribosomal proteins or their inefficient assembly yield synthetic phenotypes with *MAF1*. Interestingly, some of these genes (*TIF6* and several *RPL* genes) have previously been shown to block repression of rDNA and RP gene transcription following interruption of the secretory pathway [19],[20]. Similarly, the genetic interaction between *UTP22* and *MAF1* (Figure 2) suggests a functional relationship between the transcription and processing of the large rRNAs and the transcription of RP and RNA pol III genes. These functional associations reflect the role of Utp22 as a subunit of both the ssu processome and the CURI complex [21],[22]. Based on these results, we hypothesized that other *MAF1* SSL genes in the ribosome biogenesis category, along with genes in some of the other functional categories, might play a role in regulating the transcription of ribosomal components. Indeed, a survey of all the non-essential *MAF1* SSL genes revealed that rapamycin-mediated transcriptional repression of RP genes was substantially attenuated in *RPL20B*, *MRT4*, *KEM1*, *BUD20*, *LSM1* and *MED20* mutant strains (Figure S1A and S1B). Relative to the untreated wild type and mutant controls, northern analysis of the affected strains showed that the levels of *RPL3* and *RPL28* mRNAs following rapamycin treatment were elevated three to nine fold over wild type (Figure S1B). Along with the elevated levels of RP mRNAs that are seen in cells depleted for Utp22 and Tif6 [20],[22], it appears that a subset of *MAF1* SSL genes is associated with defects in the repression of RP gene transcription.

### Multiple Mediator Subunits Exhibit Genetic Interactions with *MAF1*

**Figure 3:**
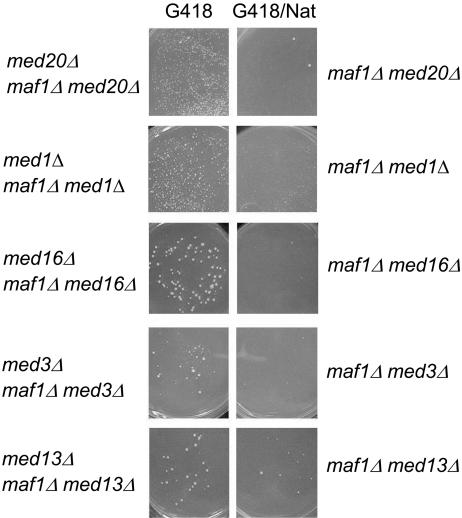
Genetic interactions between *MAF1* and multiple Mediator subunits. G418-resistant Mediator subunit deletions in strain BY4741 were crossed to a clonNat-resistant *maf1Δ* query strain (Y6338) and genetic interactions were assessed by random spore analysis [15]. Growth at 35°C (Med20) or at 37°C (all other strains) is compared on haploid selection plates containing G418 or G418 and clonNat, which selects for strains with the indicated genotypes. In the absence of effects on strain viability, approximately equal numbers of haploid colonies are expected on the two media. Images of haploid selection plates containing no antibiotics or only clonNat (which selects for *maf1Δ*) have been omitted for clarity as the growth of the *maf1Δ* single mutant is indistinguishable from wild-type. Deletion of *MED5*, *MED9*, *MED31* or *cycC* but not *MED12* also resulted in synthetic growth defects with *maf1Δ* at 37°C in the random spore assay (data not shown).

In light of the preceding observations, we were especially intrigued that Med20 (Srb2), a non-essential subunit from the head module of the Mediator complex, was among the *MAF1* SSL genes exhibiting defects in the repression of RP genes. Given that the role of the head module of Mediator and of Med20 specifically, is not typically associated with transcriptional repression, we confirmed the effect of deleting *MED20* on *RPL3* and *RPL28* mRNA levels by northern analysis of multiple biologically independent samples (Figure S1C). In these experiments, rapamycin-mediated repression in the *med20Δ* strain was reduced 2.6–5.0 fold relative to the wild-type strain. This result led us to question why only one subunit of the 25 subunit Mediator complex [23] was identified as having a genetic interaction with *MAF1* (Figure 2). Estimates of the false negative rate in SGA screens [18] and potential differences in the strength of the synthetic phenotype suggested that other Mediator subunit deletion strains might exhibit fitness defects in combination with a deletion of *MAF1*. To examine these possibilities, direct random spore tests were performed on an additional nine deletion strains representing Mediator subunits from the other three structural modules of the complex; the middle, tail and Cdk modules. Growth of the haploid meiotic products was conducted at 30°C and at elevated temperatures since we had noted that *MAF1* SSL phenotypes were frequently stronger under these conditions. This is illustrated for the *med20Δ maf1Δ* strain which shows conditional synthetic lethality at or above 35°C (Figure 3 and Figure S2B). While none of the other tested Mediator subunit deletion strains exhibited fitness defects with *maf1Δ* at 30°C, eight of the nine deletion strains showed reduced viability and/or slow growth at higher temperatures (Figure 3 and data not shown). Notably, deletion of *MED16* (*SIN4*) conferred conditional synthetic lethality at 37°C. Consistent with the fact that loss of *MED16* dissociates a set of physically interacting tail module subunits (including Med2, Med3, Med15) from the rest of the complex [24], a similar conditional synthetic phenotype was observed with deletion of *MED3*. In summary, these results extend the functional relationship between *MAF1* and *MED20* inferred from their genetic interaction at 30°C to subunits in every structural module of the Mediator complex.

### Positive and Negative Roles for Med20 in the Transcriptional Response to Rapamycin

**Figure 4:**
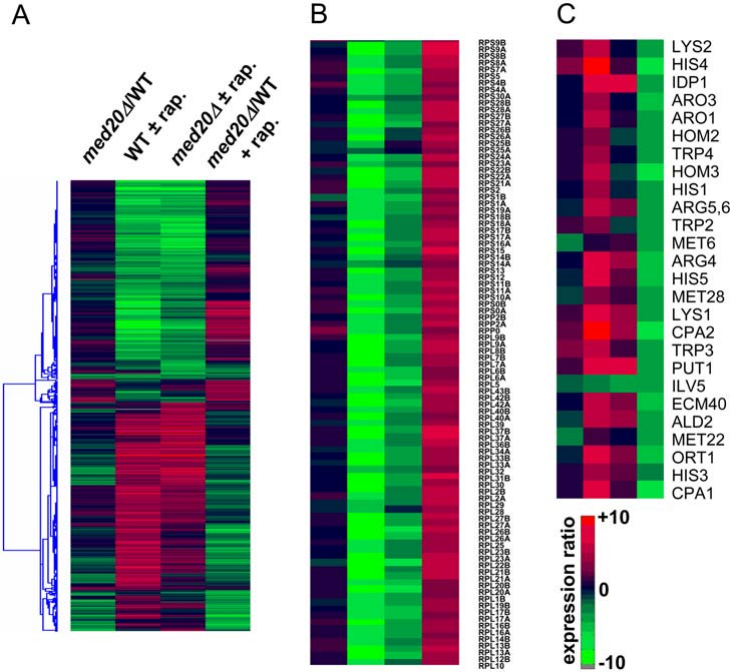
Microarray expression profiles of wild-type and *med20Δ* strains before and after treatment with rapamycin. Clustergram comparisons of gene expression profiles obtained in dye-swap experiments under four conditions; from left to right in each panel, *med20Δ/MED20*, *MED20*±rapamycin, *med20Δ*±rapamycin and *med20Δ*+rapamycin/*MED20*+rapamycin. RNA samples were prepared from cells grown at 30°C. Decreased (green) and increased (red) expression is shown relative to the wild-type strain or the untreated control. A The expression of 1420 genes that increased or decrease by two-fold or more in any one of the four pair-wise comparisons were subjected to hierarchical clustering. B The repression of RP genes (96 of 138 genes) by rapamycin was specifically attenuated in the *med20Δ* strain. The average level of repression of RP genes was only two-fold in the *med20Δ* strain versus more than six-fold for the wild-type strain. C Rapamycin induction of a subset of Gcn4-regulated genes is diminished significantly in the *med20Δ* strain. Expression ratios are compared for 25 Gcn4-regulated genes involved in amino acid biosynthesis.

The finding that multiple Mediator subunits interact genetically with *MAF1* suggests that Mediator and Maf1 function in parallel pathways. We considered that these buffering pathways might involve the transcriptional response to conditions that repress ribosome and tRNA synthesis since the role of Maf1 in repressing RNA pol III transcription entails the integration of signals that coordinately regulate these processes [4],[10],[13]. To examine the function of Med20 under repressing conditions, we conducted microarray experiments in wild-type and *med20Δ* strains that had been treated (or not) with rapamycin to inhibit TOR signaling (microarray data are available at the National Center for Biotechnology Information GEO database under accession number GSE11397). Messenger RNA representing each of the four conditions (wild-type, *med20Δ*, ±rapamycin) was used to prepare Cy5- and Cy3-labeled cDNAs. Pairs of dye-reversed cDNA samples were then hybridized to spotted arrays of yeast ORFs. The resulting data were filtered to select genes whose expression increased or decreased two-fold or more in any of the four pairwise comparisons (*med20Δ/MED20*, *MED20*±rapamycin, *med20Δ*±rapamycin and *med20Δ*+rapamycin/*MED20*+rapamycin, Table S2) and then subjected to hierarchical clustering (Figure 4). Several important conclusions emerged from these experiments: (i) Deletion of *MED20* does not appreciably affect the global pattern of gene expression under normal growth conditions: Only 116 genes were affected beyond the two-fold cutoff in our experiments. Using the same criteria, even fewer genes were affected in a previously reported comparison of unstressed wild-type and *med20Δ* strains [25] (see Text S1). An analysis of the combined datasets for shared GO Bioprocess terms indicates that major cellular process such as ribosome biogenesis and assembly, translation, transcription, the organization and biogenesis of the nucleus, membranes and the cytoskeleton, as well as other processes, are largely or entirely unaffected by deletion of *MED20* (Table S3). In particular, the expression of genes involved in the synthesis, processing or function of RNA pol III transcripts is not affected in the *med20Δ* strain and RNA pol III gene transcription is effectively repressed by rapamycin treatment in the absence of *MED20* (Figure S2A). Thus, a function for Med20 in RNA pol III transcription can be discounted as an explanation for its genetic interaction with *MAF1*. (ii) Rapamycin treatment of the wild-type strain showed a characteristic response with the induction and repression of specific sets of genes representing ∼20% of the genome (Figure 4, Text S1, and Figure S3). As reported in other studies ([26] and references therein), RP genes and genes of the *Ribi* regulon involved in ribosome biogenesis and related functions were strongly repressed by rapamycin while general amino acid control genes and many other Gcn4-regulated genes were strongly induced (Text S1). (iii) Within the group of rapamycin-responsive genes, deletion of *MED20* selectively diminished the level of induction and repression (Figure 4). For example, the level of activation of a subset of Gcn4-regulated genes was attenuated significantly: Of the 197 genes whose expression after rapamycin treatment was 2–12 fold lower in the *med20Δ* strain than in the wild-type strain, 74 (38%) were Gcn4 targets (*p* = 1E-32). Notably, genes involved in amino acid biosynthesis and related metabolic processes were highly enriched within this group (25 genes, GOID 6519, *p* = 7.34E-19, Figure 4C). These results are consistent with the requirement for Mediator in the activation of specific Gcn4-regulated genes [24],[27] and extend this requirement to a larger group of Gcn4-target promoters by identifying a critical role for Med20 in their activation following rapamycin treatment. In addition, we found 97 out of 138 RP genes among the 170 genes whose expression following rapamycin treatment was 2 to 6-fold higher in the *med20Δ* strain than in the treated wild-type strain (Figure 4B, Table S2). In agreement with our expectations from northern blotting of specific RP mRNAs (Figure S1), deletion of *MED20* compromises the repression of RP genes by rapamycin. The attenuated repression of RP genes in the absence of Med20 is highly specific as repression of genes in the *Ribi* regulon, which show nearly identical transcriptional responses under many different environmental conditions [5],[28], was unaffected: Similar numbers of *Ribi* genes were down-regulated by rapamycin in both wild-type and *med20Δ* strains (125 and 133 genes, respectively, above the two-fold cutoff). Moreover, only six *Ribi* genes (statistically equivalent to a random distribution) were found among the 170 genes exhibiting a two-fold or higher difference in expression when comparing rapamycin-treated *med20Δ* and wild-type strains. Thus, the data indicate a unique and highly selective requirement for a head module subunit of Mediator in the repression of RP gene transcription by rapamycin.

### Med20 Is Required for Efficient Repression of RP Genes under Multiple Conditions

**Figure 5:**
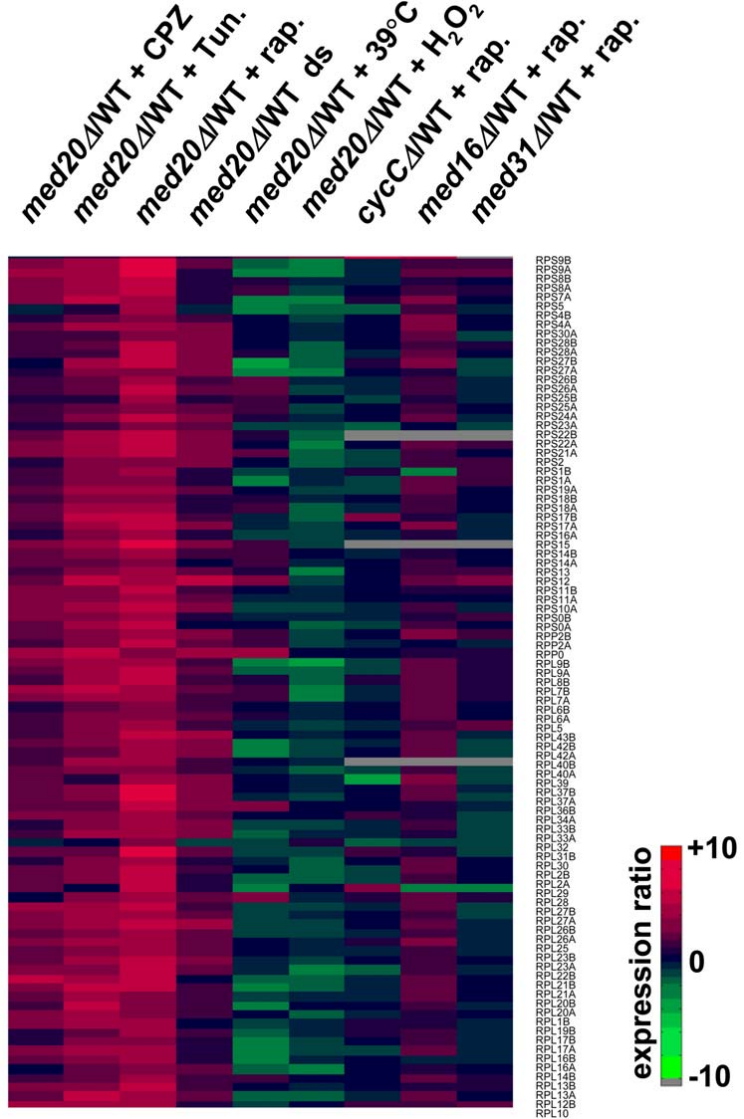
Analysis of RP gene expression in Mediator subunit deletion strains under different repressing conditions. Clustergram comparison of expression ratios are shown for 96 RP genes. Changes in expression (increased in red and decreased in green) are shown relative to the treated wild-type strain under six repressing conditions (rapamycin, tunicamycin, CPZ, post-diauxic shift, transient heat shock and hydrogen peroxide, see Methods). The effect of rapamycin is compared in four Mediator subunit deletion strains. Each deleted subunit represents a different structural module of the complex (*med20* in the head, *med31* in the middle, *med16* in the tail and *cycC* in the Cdk module). Except for the transient heat shock, all strains were grown at 30°C.

RP genes are coordinately down-regulated under a wide variety of nutrient-limiting and stress conditions [1],[28]. Virtually all of these conditions also cause Maf1-dependent repression of RNA pol III transcription [4],[10],[13]. Given the essential function of Maf1 in conveying the signals from diverse pathways to the RNA pol III transcription machinery, we were interested to know whether Med20 serves a general or condition-specific role in repressing RP gene transcription. Microarray profiles were generated from pairs of fluor-reversed experiments where wild-type and *med20Δ* strains were treated with tunicamycin, chlorpromazine (CPZ), hydrogen peroxide or mild heat stress (29–39°C). In addition, expression profiles of the two strains were compared following the diauxic shift from glucose fermentation to respiratory metabolism. All of these conditions repress dramatically the transcription of RP genes [1],[28]. Clustergram comparisons of 1063 genes whose expression differed two-fold or more in any of the six conditions (including rapamycin), revealed similar profiles for rapamycin, tunicamycin, and CPZ treatments along with post-diauxic cells (Figure S4). These similarities were especially pronounced for RP genes (Figure 5), which were highly enriched among the genes exhibiting attenuated repression in the *med20Δ* strain (*p* values ranged from 1.85E-9 to 1.7E-128). These data suggest an integral role for Med20 in the repression of RP gene transcription under four of the six conditions. In contrast, no significant contribution of Med20 was evident in the down-regulation of RP genes under conditions of oxidative or mild heat stress (Figure 5). The lack of an effect on RP genes in these experiments is apparently specific since deletion of *MED20* clearly affected other responses (Figure S4). For example, the induction of many heat shock genes was increased in the *med20Δ* strain following heat stress (11 out of 62 genes above the two-fold cutoff, *p* = 2.42E-8, Table S4). The recruitment of Mediator to heat shock genes and its requirement for gene activation by heat stress is well known [29],[30] although a role for Med20 in this process has not previously been described. Similarly, the characteristic induction of many oxidative stress and heat shock response genes in hydrogen peroxide-treated cells was also increased substantially in the *med20Δ* strain (17 out of 260 genes, *p* = 1.16E-7, Table S4). The contribution of Med20 in this response is consistent with previous work demonstrating the importance of Cdk module inactivation for the induction of oxidative stress response genes [31].

Expression profiling of Mediator subunit deletion strains under normal growth conditions has revealed epistatic relationships and a pathway of signal transduction between specific Mediator subunits [25]. This led us to examine the role of subunits in the middle, tail and Cdk modules of Mediator in the repression of RP gene transcription by rapamycin. In contrast to the deletion of *MED20* in the head module, deletions of *MED31* and *CYCC* in the middle and Cdk modules, respectively, had no detectable effect on the repression of RP genes at 30°C relative to the wild-type strain (Figure 5, Table S5). Repression of RP gene transcription was also examined by northern analysis in a strain deleted for *MED13* (*SRB9*). This subunit in the repressive Cdk module is a direct target of protein kinase A (PKA) and TOR kinase signaling is thought to control ribosome biogenesis in part by antagonizing the Ras/PKA pathway [32],[33]. However, the wildtype and *MED13* deletion strains showed no differences in their response to rapamycin (data not shown). These results are consistent with the genetic interaction data in that synthetic phenotypes between *MAF1* and Mediator subunits from the middle and Cdk modules were not apparent at 30°C but were only revealed at 37°C (Figure 3). Deletion of *MED16* (*SIN4*) in the tail module showed a modest reduction in the extent of repression of RP genes at 30°C (1.5±0.2 fold relative to wild-type for the 121 RP genes yielding signals in the repressed gene set, Figure 5, Table S5). This effect is consistent with the difference in the strength of the synthetic phenotypes of the *med16Δ maf1Δ* and the *med20Δ maf1Δ* strains at 30°C. Considering that these double mutant strains are both synthetically lethal at elevated temperatures (Figure 3), the findings indicate that Med16 plays a minor role relative to Med20 in rapamycin repression of RP genes under normal growth conditions.

## Discussion

The large (>1 MDa) Mediator complex is organized into four structurally distinct modules, the head, middle, tail and Cdk modules, and functions to transduce regulatory information from DNA–bound activators and repressors to the general RNA pol II transcription machinery [23],[34],[35]. In addition to its role in regulating transcription, studies with temperature-sensitive head module subunits (e.g. Med17/Srb4) have suggested that Mediator is essential for all transcription in vivo [36]. This is supported by the ability of Mediator to stimulate basal transcription in vitro and by the temperature-sensitivity of this stimulation in extracts of an *srb4-138* mutant strain [37]. Recently, the ubiquitous function of Mediator in transcription has been questioned based on chromatin immunoprecipitation (ChIP) experiments showing that the association of Mediator and RNA pol II with many actively transcribed genes is not correlated [29]. Indeed, the observation that Mediator associates very poorly with the enhancer regions of RP and glycolytic genes, which together account for 50% or more of RNA pol II transcription in actively growing cells [1], has suggested that Mediator may not be required for their transcription [29]. Other groups have reported Mediator associations with the coding regions of highly expressed genes [38],[39]. However, Mediator binding ratios in RP coding regions are also very low (e.g. an average binding ratio of 1.3 was determined from 28 experiments versus 4.3 from 13 experiments for RNA pol II, [38]). Our examination of the molecular basis for synthetic fitness defects between Maf1 and different Mediator subunits has revealed a prominent role for a non-essential head module subunit, Med20, in the repression of RP gene transcription under several different conditions. Together with similar observations for a tail module subunit, Med16, our results bear directly on the issue of Mediator involvement in RP gene transcription.

Studies published to date have attributed the head module of Mediator with a largely positive role in transcription [25]; negative regulation by head module subunits under specific nutritional or environmental conditions has not been reported. We find that Med20 functions both positively and negatively on different subsets of genes under a range of environmental conditions (Figures 4, 5 and Figure S4). For the induction of Gcn4-regulated genes by rapamycin, the effect of deleting *MED20* is consistent with other reports showing reduced recruitment of Mediator by promoter-bound Gcn4 and diminished transcriptional activation of Gcn4-controlled genes when Med20 or subunits of the tail module are deleted [24],[27]. For RP genes, where the association of Mediator by ChIP is poor, the evidence supporting a direct role for Mediator in repression is based on the specificity of the response and the fact that changes in gene expression in unstressed *med20Δ* cells are minimal and are unlikely to impact RP gene transcription ([25] and see below). RP and *Ribi* genes show nearly identical transcription responses to environmental and genetic perturbation [5],[28] even though the promoters of these genes generally contain different cis-acting elements (Rap1 and/or Abf1 sites for RP genes, PAC and/or RRPE elements for *Ribi* genes). Despite these differences, both sets of genes are regulated by Sfp1 in response to nutrients and stress conditions including rapamycin [5]. The fact that the *Ribi* genes are repressed normally by rapamycin in *med20Δ* strains whereas the repression of RP genes is attenuated indicates that the TOR signaling pathway mediating this response is not impaired and suggests that the differences in repression are likely independent of Sfp1. Molecular genetic, biochemical and structural studies indicate that deletion of *MED20* does not significantly perturb the overall structure of Mediator: The absence of Med20 does not affect the assembly of other head module subunits into a stable complex [40] or the association of the head module with other modules of Mediator [23],[24],[41]. These data together with the crystal structure of a Med8-C-Med18-Med20 submodule and EM images suggest that Med20 occupies a peripheral position in the head module and in the complete complex [40],[41]. In support of the limited structural effects of deleting *MED20*, the expression profile of unstressed *med20Δ* cells shows that only a small number of genes are affected (Figure 4, Table S3, [25]). Importantly, the annotated functions of this small group of genes do not reveal changes in transcription or other processes that might indirectly account for the attenuated repression of RP genes. Given the data indicating that Mediator is essential for all RNA pol II transcription [36],[37], our findings are consistent with a direct effect of Mediator on RP gene transcription under specific repressing conditions. However, as noted above, Mediator subunits are not efficiently cross-linked to RP genes in ChIP assays [29],[38],[39]. We infer from this that the nature of the interactions between Mediator and RP genes is fundamentally different from other genes that exhibit robust Mediator ChIP signals. One possibility is that the function of Mediator on RP genes may require only a transient association. Alternatively, the physical nature of the interaction between Mediator and the nucleoprotein complexes assembled on RP genes may not be compatible with its efficient crosslinking. Focusing on the prominent effect of Med20 (Figure 4), a third explanation is that this protein functions independently of the Mediator complex in the repression of RP genes. While we cannot exclude this possibility, it does not account for the attenuated repression observed when the tail module subunit Med16 is deleted (Figure 5 and Table S5). Moreover, the synthetic interactions between *MAF1* and Mediator subunits representing each structural module of the complex imply that a function of Mediator, not just Med20, underlies the functional relationship with Maf1. As discussed below, a growing body of evidence supports the view that this relationship involves the coordinate regulation of ribosome and tRNA synthesis. Given the role of Maf1 in repressing RNA pol III transcription, an analogous role for Mediator in RP gene transcription is consistent with the typical interpretation of SSL interactions, namely, that the genes function in parallel pathways. Therefore, we suggest that Mediator and Maf1 function at the downstream end of distinct signaling pathways to negatively regulate RP mRNA and tRNA synthesis, respectively.

Unlike deletion of *MAF1*, which quantitatively blocks repression of RNA pol III transcription [4], deletion of *MED20* only attenuates repression of RP genes. Thus, the signaling pathways that repress RP genes must have multiple targets within the RNA pol II transcription machinery. Besides Mediator, what other transcriptional targets are involved in the repression of RP genes? Previous work has identified Crf1 as a TOR kinase-regulated corepressor of RP genes [7]. We tested whether deletion of *CRF1*, either by itself or in combination with a deletion of *MED20* could affect rapamycin-mediated repression of RP genes in the SGA strain background (S288C). Although we generated the *crf1Δ* strains *de novo*, northern analysis of multiple RNA samples did not reveal any quantitative differences compared to the controls (data not shown). This result is consistent with findings in the W303 strain background [42], indicating that the corepressor function of Crf1 at RP genes is strain-specific. Other observations suggest that the TFIID complex may participate in the repression of RP genes. TFIID occupancy of RP genes is high [43] and the transcription of RP genes is strongly TFIID-dependent [44]. This dependence reflects both a core promoter recognition function and a coactivator function of TFIID on these promoters [44],[45]. Our SGA screens identified synthetic fitness defects between *MAF1* and five *TAF*s, two of which (*TAF8* and *TAF11*) are unique to the TFIID complex [43]. The basis for these genetic interactions may be similar to *MED20*. In other words, synthetic growth defects may result, in part, from the inability to repress RNA pol III transcription coupled with attenuated repression of RP gene transcription. This interpretation is consistent with the identification of genetic interactions between *MAF1* and genes in the ribosome biogenesis category (*TIF6* and several *RPL* genes), where functional insufficiencies are known to block the repression of rDNA and RP gene transcription following interruption of the secretory pathway [19],[20]. Another link to transcriptional control of ribosome synthesis is provided by the genetic interaction between *UTP22* and *MAF1*. *UTP22* encodes one of three essential gene products (the others being Ifh1 and Rrp7) that associate with casein kinase II (CK2) to form the CURI complex [22]. This complex is thought to coordinate two parallel pathways necessary for ribosome synthesis, namely, the transcription and processing of pre-rRNA and the transcription of ribosomal protein genes. The presence of CK2 in the complex further strengthens the proposed functional association between *MAF1* and ribosome synthesis based on studies of CK2 in the transcriptional response of RNA pols I and III to DNA damage [46]. Finally, we found that nearly one-fifth of the *MAF1* SSL genes identified in the non-essential gene-deletion array are associated with defects in the repression of RP gene transcription (Figure S1). These observations support our hypothesis that the genetic interaction between *MAF1* and *MED20* is related to the combination of defects in the repression of RNA pol III and RP gene transcription. This interpretation does not exclude the possibility that other changes in the *maf1 med20* double mutant strain may contribute to its synthetic phenotype. Given the genetic interactions of *MAF1* with subunits of Mediator and the TFIID complex, our identification of a negative regulatory function for Med20 at RP genes suggests a possible relationship with TFIID in this process since the head module of Mediator contains a multipartite TBP-binding site that includes a direct interaction between TBP and Med20 [41].

In addition to genes involved in ribosome biogenesis and transcription, our SGA analysis of *MAF1* revealed a significant functional relationship with enzymes involved in tRNA modification (Figure 2, Table S1). This group of interactions supports a previous proposal concerning the paradoxical anti-suppressor phenotype of *maf1Δ* strains. Loss of *MAF1* function causes a significant increase in the cellular level of mature tRNA (from ∼10% to ∼25% of total RNA) yet the activity of the *SUP11-o* nonsense suppressor decreases [47]. This anti-suppressor phenotype was suggested to result from incomplete isopentenylation of an adenine base (A37, adjacent to the anticodon) which is important for tRNA decoding efficiency. A recent study of synthetic interactions between certain non-essential tRNA modifying enzymes has highlighted their function in tRNA stability and cell survival [48]. Our findings demonstrate that tRNA modifications become critical in the *maf1Δ* strain since the additional loss of any one of six tRNA modifying enzymes results in a synthetic growth defect (Figure 1). We anticipate that an analysis of the genetic interactions between *MAF1* and this group of enzymes will provide new insights into their biological function.

## Materials and Methods

### SGA Methods

Triplicate SGA screens of a *maf1Δ* query strain (Y6338 *Matα can1Δ::MFA1pr-HIS3 lyp1Δ ura3Δ0 leu2Δ0 his3Δ1 met15Δ0 maf1Δ::natR*) were performed against the non-essential gene-deletion array (∼4700 strains) and against an array of conditionally-expressed essential genes (∼800 Tet-promoter strains). Each screen was conducted with duplicate copies of the array in a 768 colony per plate format as described previously [15],[17],[18]. In Tet-promoter array screens, the haploid double mutant strains were scored for growth on medium with and without doxycycline (10 µg/ml). Visual inspection and computer-based analysis of digital images was used to identify double mutant strains exhibiting fitness (growth) defects [18] relative to a control set of double mutants obtained using strain Y5518 (*Matα mfa1Δ::MFA1pr-HIS3 lyp1Δ ura3Δ0 leu2Δ0 his3Δ1 met15Δ0 can1Δ::natR*). Candidate synthetic genetic interactions were validated by random spore analysis [15],[17] at either 30°C or at elevated temperatures (35–37°C) since this enhanced the severity of the synthetic fitness defect in many cases. The enrichment of GO Bioprocess terms in the *MAF1* SSL gene set was calculated by hypergeometric distribution with aid of the MIPS Functional Catalogue Database.

### Construction of the *MAF1* Genetic Interaction Network

Random spore-validated *MAF1* SSL genes were queried against the BioGRID Database version 2.0.23 (released Jan 3, 2007) to compile a list of 4012 interactions involving 1225 genes. Interactions were found for all but two *MAF1* SSL genes (Fyv5, and YGL007W). The set of interactions was superimposed onto the *MAF1* SSL gene set using Osprey software and filtered to reveal interactions between nodes in the *MAF1* genetic interaction network.

### Microarray Experiments

Strain BY4741 (*Mata ura3Δ0 leu2Δ0 his3Δ1 met15Δ0*) and isogenic deletion strains (*xxxΔ:kanR*) were grown in YPD at 30°C to an optical density (A600) of ∼0.2 before addition of drugs or drug vehicle, unless otherwise indicated. Treatments with rapamycin (0.2 µg/ml from a 1 mg/ml stock solution in DMSO, AG Scientific) and CPZ (250 µM from a 500 mM stock solution in water, Sigma) were for 1 hour [4]. Treatments with hydrogen peroxide (0.32 mM, Sigma) and tunicamycin (2.5 µg/ml from a 5 mg/ml stock in 75% methanol, Sigma) were for 30 min. and 90 min. respectively [4],[28]. A transient mild heat shock treatment of cells growing at 29°C was achieved by centrifugation and resuspension in pre-warmed, 39°C medium for 20 min. [28]. To compare cells following the diauxic shift, an early log culture (OD600 = 0.01) was grown for 48 hours at 30°C and then harvested. Detailed procedures for culturing cells, RNA preparation, hybridization, image acquisition and data processing for microarrays have been described [49]. Replicates of each sample were performed using a fluor-reversal strategy [50]. Microarray data have been deposited in the Gene Expression Omnibus Database under accession number GSE11397.

## Supporting Information

Figure S1 Northern analysis of RP genes in wild-type and *MAF1* SSL strains before and after rapamycin treatment.
(0.15 MB PDF)

Figure S2 Transcription of a tRNALeu gene is robustly repressed by rapamycin in the med20 strain.
(0.07 MB PDF)

Figure S3 Genes induced and repressed by rapamycin treatment of strain S288c.
(0.10 MB PDF)

Figure S4 Clustergram comparison of *med20Δ* versus wild-type expression ratios under different environmental conditions.
(0.05 MB PDF)

Table S1 Phenotypes and functions of *MAF1* SSL genes.
(0.03 MB PDF)

Table S2 Expression ratios (log base 10) comparing *med20Δ* and wild-type strains before and after rapamycin treatment.
(0.43 MB PDF)

Table S3 Yeast GO bioprocess terms represented in merged *med20Δ* versus wild-type datasets.
(0.04 MB PDF)

Table S4 Expression ratios (log base 10) comparing *med20Δ* versus wild-type strains under different repressing conditions.
(0.41 MB PDF)

Table S5 Expression ratios of ribosomal protein genes comparing different Mediator subunit deletions versus wild-type after rapamycin treatment.
(0.04 MB PDF)

Text S1 Supporting text.
(0.10 MB PDF)
