# Supplementary figures and images for: Genetic Interactions of MAF1 Identify a Role for Med20 in Transcriptional Repression of Ribosomal Protein Genes
Source: PLoS Genet. 2008 Jul 4;4(7):e1000112. doi: 10.1371/journal.pgen.1000112 (PMC2435279; doi:10.1371/journal.pgen.1000112)

Fig. S1

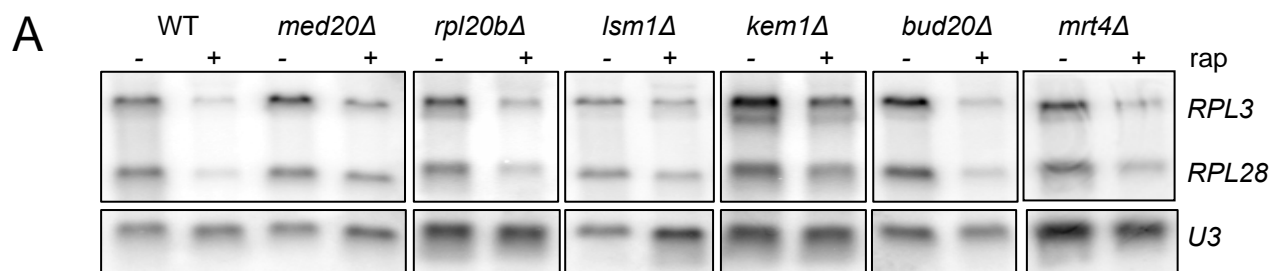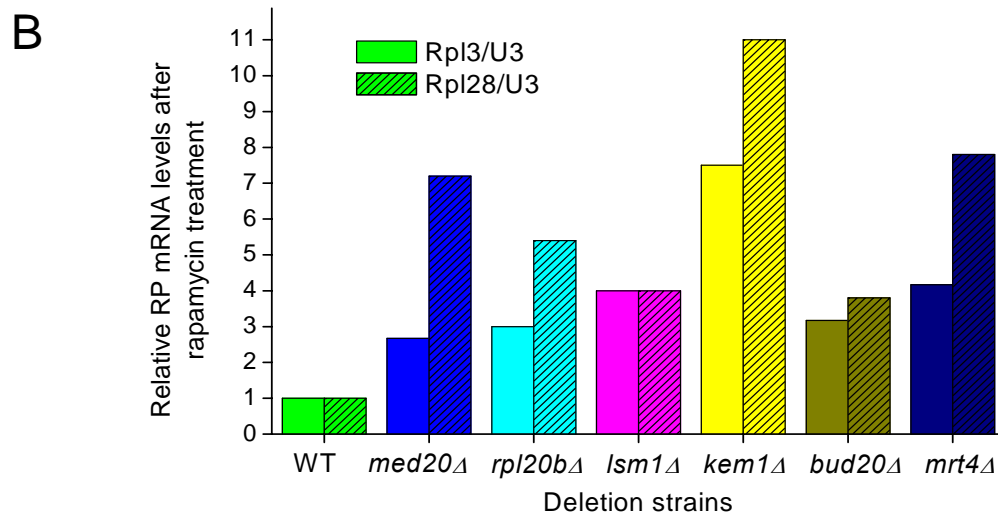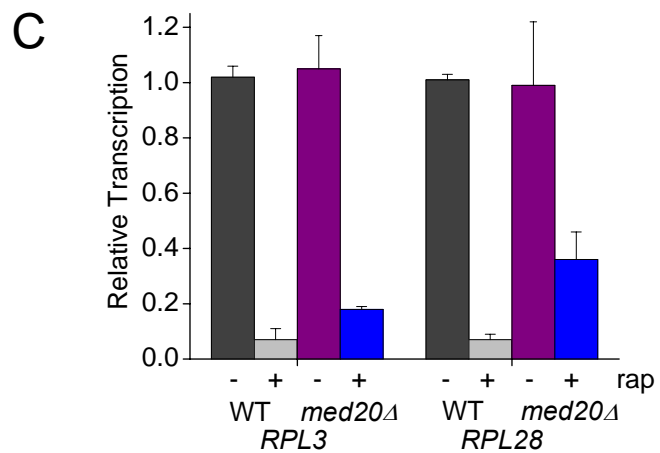

Supplement: Figure S1 — Northern analysis of RP genes in wild-type and MAF1 SSL strains before and after rapamycin treatment. (0.15 MB PDF) [file pgen.1000112.s001.pdf]

Fig. S2

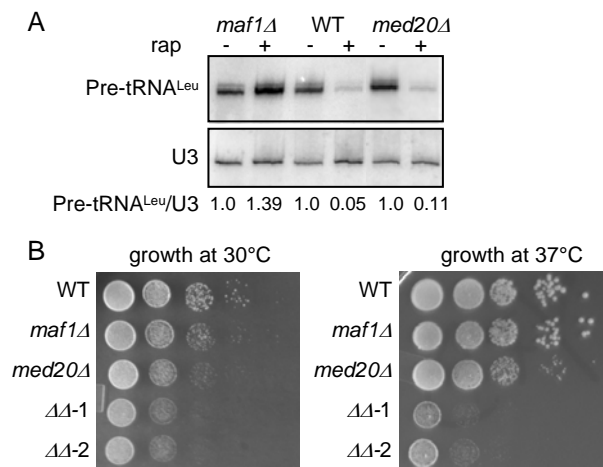

Supplement: Figure S2 — Transcription of a tRNALeu gene is robustly repressed by rapamycin in the med20 strain. (0.07 MB PDF) [file pgen.1000112.s002.pdf]

Fig. S3

Rap. Repressed

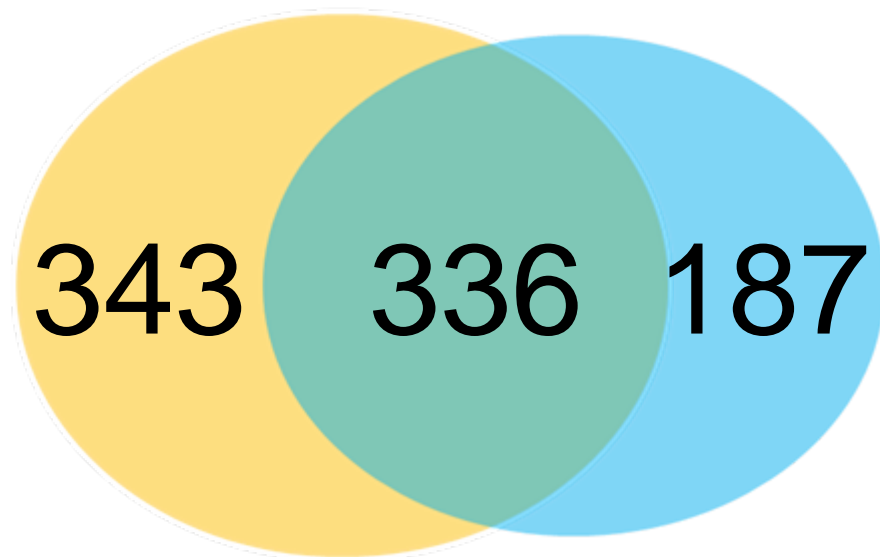

Chen & Powers

Willis et al.,

Rap. Induced

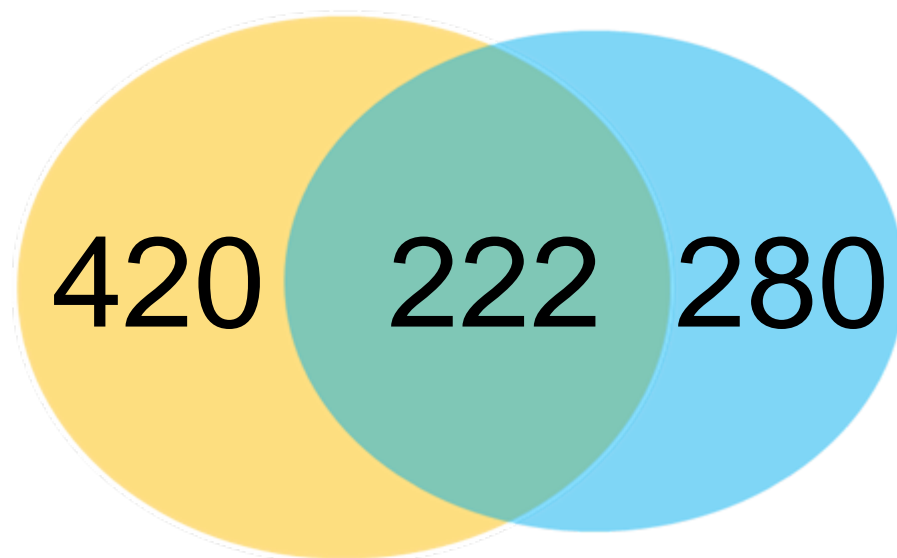

Chen & Powers

Willis et al.,

Supplement: Figure S3 — Genes induced and repressed by rapamycin treatment of strain S288c. (0.10 MB PDF) [file pgen.1000112.s003.pdf]

Fig. S4

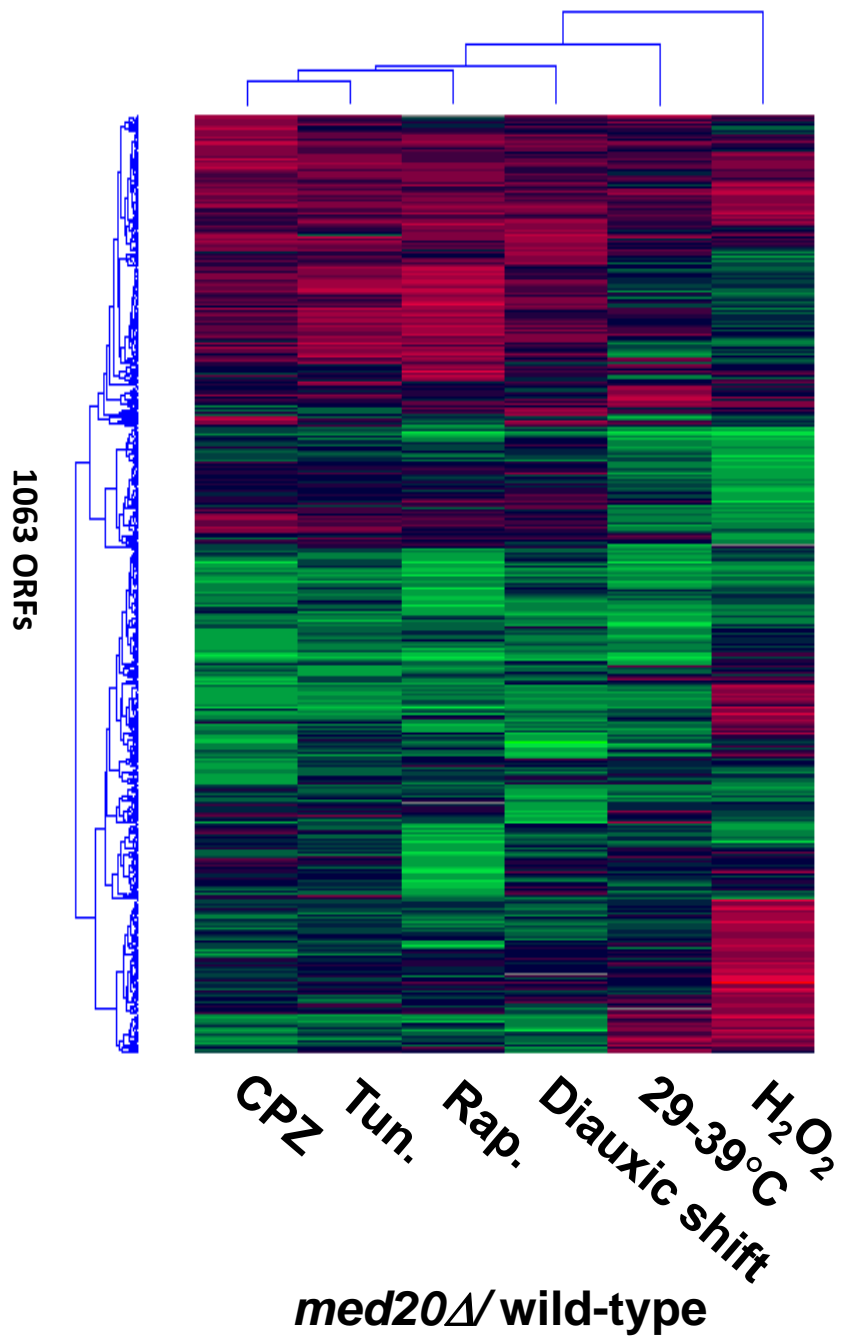

Supplement: Figure S4 — Clustergram comparison of med20Δ versus wild-type expression ratios under different environmental conditions. (0.05 MB PDF) [file pgen.1000112.s004.pdf]
