# Supplementary material for: Genetic Interactions of MAF1 Identify a Role for Med20 in Transcriptional Repression of Ribosomal Protein Genes
Source: PLoS Genet. 2008 Jul 4;4(7):e1000112. doi: 10.1371/journal.pgen.1000112 (PMC2435279; doi:10.1371/journal.pgen.1000112)
Supplement: Table S1 — Phenotypes and functions of MAF1 SSL genes. (0.03 MB PDF) [file pgen.1000112.s005.pdf]

Table S1
Phenotypes and Functions of *MAF1* SSL Genes

Gene	Deletion Lethal/Viable	Random Spore Analysis[a]	Gene Function	Biological process
MAK21	L	SS	unknown function	ribosome biogenesis, large subunit
TIF6	L	SS	ribosomal large subunit binding	ribosome biogenesis, large subunit
MAK16	L	SS	unknown function	ribosome biogenesis, large subunit
YTM1	L	SS	unknown function	ribosome biogenesis, large subunit
BUD20	V	SS-SS	unknown function	ribosome biogenesis, large subunit
RRP1	L	SS	unknown function	ribosome biogenesis, large subunit
MRT4	V	SS-SS	unknown function	ribosome biogenesis, large subunit & mRNA decay
KEM1	V	SS*	5'-3' exoribonuclease, 35S rRNA processing	ribosome biogenesis & mRNA decay
YOR235W	V	SS	dubious ORF, deletion overlaps SNR17a	ribosome biogenesis, small subunit
UTP15	L	SS	snoRNA binding, processing of 20S pre-rRNA	ribosome biogenesis, small subunit
UTP5	L	SS	snoRNA binding, processing of 20S pre-rRNA	ribosome biogenesis, small subunit
UTP22	L	SS	snoRNA binding, processing of 20S pre-rRNA	ribosome biogenesis, small subunit
PNO1	L	SS	pre-18S rRNA processing	ribosome biogenesis, small subunit
NSR1	V	SS*	rRNA processing	ribosome biogenesis
PWP1	L	SS	unknown function, rRNA processing	ribosome biogenesis
GRC3	L	SS	unknown function, rRNA processing	ribosome biogenesis
LRP1	V	SS-ts	exosome	ribosome biogenesis
RRP43	L	SS	exosome	ribosome biogenesis
RRP46	L	SS	exosome	ribosome biogenesis
HCR1	V	SS*	processing of 20S pre-rRNA, translation eIF 3 complex	ribosome biogenesis & translation
RPS19B	V	SS*	ribosomal protein	translation
RPS17A	V	SS*	ribosomal protein	translation
RPL43A	V	SS-SS	ribosomal protein	translation
RPL20B	V	SS*	ribosomal protein	translation
RPS16A	V	SS*	ribosomal protein	translation
TIF35	L	SS	translation initiation factor 3(eIF3) subunit	translation
KRS1	L	SS	lysine-tRNA ligase	aminoacylation
DED81	L	SS	asparaginyl-tRNA aminoacylation	aminoacylation
LSM1	V	SS-ts	Sm like protein	cytoplasmic mRNA decay
PUS1	V	SS-SL	pseudouridylate synthase, tRNA positions 26-28, 34-36, 65, 67	tRNA modification

PUS4	V	SS-SS	pseudouridylate synthase, tRNA position 55	tRNA modification
PUS5	V	SS*	pseudouridine synthase, mitochondrial 21S rRNA	rRNA modification
TAN1	V	SS-ts	tRNA acetyltransferase	tRNA modification
TRM1	V	SS*	tRNA (guanine-N2-)-methyltransferase	tRNA modification
TRM10	V	SS*	tRNA (guanine-N1-)-methyltransferase	tRNA modification
GCD10	L	SS-SL	tRNA (1-methyladenosine) methyltransferase	tRNA modification
LOS1	V	SS*	tRNA karyopherin	tRNA nuclear export
NUP133	V	SS*	nuclear pore complex	nucleocytoplasmic transport
SRB2	V	SS-SL	mediator subunit	RNA polymerase II transcription
SPT10	V	SS-ts	putative histone acetyltransferase	RNA polymerase II transcription
SFL1	V	SS*	transcription factor homologous to Hsf1	RNA polymerase II transcription
MGA2	V	SS*	transcriptional activator	RNA polymerase II transcription
TAF6	L	SS	TFIID and SAGA complexes	RNA polymerase II transcription
TAF8	L	SS	TFIID complex	RNA polymerase II transcription
TAF9	L	SS	TFIID and SAGA complexes	RNA polymerase II transcription
TAF11	L	SS	TFIID complex	RNA polymerase II transcription
TAF12	L	SS	TFIID and SAGA complexes	RNA polymerase II transcription
UMP1	V	SS*	maturation of 20S proteasome	ubiquitin-dependent protein catabolism
PRE5	L	SS	proteasome endopeptidase, 20S core	ubiquitin-dependent protein catabolism
RPT4	L	SS	ATPase, 19S regulatory particle	ubiquitin-dependent protein catabolism
RPN8	L	SS	proteasome endopeptidase, 19S regulatory particle	ubiquitin-dependent protein catabolism
RPN11	L	SS	proteasome endopeptidase, 19S regulatory particle	ubiquitin-dependent protein catabolism
MRK1	V	SS-SS	Glycogen synthase kinase 3 (GSK-3) homolog	stress response
MDM34	V	SS*	mitochondrial outer membrane protein	mitochondrion organization and biogenesis
PCP1	V	SS*	mitochondrial serine protease	mitochondrion organization and biogenesis
YOR1	V	SS*	xenobiotic-transporting ATPase	transport
FYV5	V	SS*	unknown function	ion homeostasis
GYP1	V	SS-SS	Rab GTPase activator	vesicle-mediated transport
PSA1	L	SS	mannose-1-phosphate guanyltransferase	cell wall biosynthesis
ACS2	L	SS	Acetyl-coA synthetase isoform	acetyl-CoA biosynthesis

ERG12	L	SS	mevalonate kinase	ergosterol biosynthesis
YGL007W	V	SS-ts	unknown function	unknown process
YGL250W	V	SS-SS	unknown function	unknown process
ILM1	V	SS*	unknown function	unknown process

[a]SS For non-essential genes, synthetic sick at 30°C; For essential genes, synthetic sick in the presence or absence of doxycycline

SS-SS synthetic sick at 30°C and at 35-37°C

SS* synthetic sick at 35-37°C

SS-ts synthetic phenotype at 30°C enhanced at elevated temperatures

SS-SL For non-essential genes, synthetic sick at 30°C, lethal at 35-37°C; For essential genes, synthetic sick -doxycycline, lethal + doxycycline
