# Supplementary material for: Genetic Interactions of MAF1 Identify a Role for Med20 in Transcriptional Repression of Ribosomal Protein Genes
Source: PLoS Genet. 2008 Jul 4;4(7):e1000112. doi: 10.1371/journal.pgen.1000112 (PMC2435279; doi:10.1371/journal.pgen.1000112)
Supplement: Table S2 — Expression ratios (log base 10) comparing med20Δ and wild-type strains before and after rapamycin treatment. (0.43 MB PDF) [file pgen.1000112.s006.pdf]

Table S2
Expression ratios (log base 10) comparing med20Δ and wild-type strains before and after rapamycin treatment
The data have been filtered to select genes whose expression increased or decreased two fold or more in any of the four pairwise comparisons

UNIQID	Gene Name	med20/WT	WT +/- rapamycin	med20 +/- rapamycin	med20/WT + rapamycin
YPL220W	RPL1A	0.239892	-0.542612	-0.257786	NaN
YPL192C	PRM3	0.028019	-0.089602	-0.371525	NaN
YPL081W	RPS9A	0.010845	-1.184456	-0.370688	0.792405
YLR185W	RPL37A	0.097813	-0.966224	-0.288388	0.705401
YHR061C	GIC1	-0.13448	-0.062068	0.034822	0.688293
YDR500C	RPL37B	0.083627	-1.047937	-0.385713	0.688107
YLL009C	COX17	0.495923	-0.054028	0.147178	0.682061
YBL071C	YBL071C	0.250918	-0.163865	-0.17553	0.668368
YBR189W	RPS9B	-0.002531	-1.035597	-0.329334	0.661152
YOL040C	RPS15	0.078857	-0.950487	-0.193118	0.637436
YLR406C	RPL31B	0.053659	-0.97134	-0.426663	0.635714
YIL018W	RPL2B	0.038804	-0.862459	-0.259751	0.622047
YFR031C-A	RPL2A	0.148142	-0.769675	-0.262356	0.582971
YGL189C	RPS26A	0.064406	-0.845315	-0.109832	0.577591
YGR214W	RPS0A	0.152945	-0.913992	-0.442015	0.576644
YGR034W	RPL26B	0.058058	-1.037908	-0.596379	0.570964
YOL039W	RPP2A	0.130512	-0.787855	-0.248768	0.56857
YKR057W	RPS21A	0.166726	-0.959117	-0.382545	0.563017
YFL034C-A	RPL22B	-0.084047	-1.026742	-0.584272	0.557962
YDL082W	RPL13A	0.166239	-0.839425	-0.314712	0.557959
YLR367W	RPS22B	-0.062151	-1.078866	-0.502795	0.548732
YBR048W	RPS11B	0.041274	-0.773734	-0.298046	0.546169
YPL249C-A	RPL36B	0.092176	-0.749451	-0.206364	0.545808
YJR094W-A	RPL43B	0.079206	-0.788469	-0.295404	0.544692
YDR471W	RPL27B	0.081062	-0.85323	-0.43556	0.540626
YML024W	RPS17A	0.047923	-0.842447	-0.279956	0.538612
YER102W	RPS8B	0.117025	-0.76625	-0.250779	0.536283
YLL045C	RPL8B	0.040313	-0.937432	-0.377371	0.533813
YPL079W	RPL21B	0.071874	-0.923933	-0.287453	0.532388
YGL147C	RPL9A	0.039564	-0.996145	-0.318171	0.521344
YOR293W	RPS10A	0.122578	-0.853531	-0.402153	0.520309
YHR021C	RPS27B	0.069041	-0.921556	-0.378412	0.517029
YOR167C	RPS28A	0.060191	-0.774578	-0.187935	0.514154
YDR447C	RPS17B	0.082807	-0.754981	-0.285331	0.513686
YFR032C-A	RPL29	-0.025424	-0.546459	-0.188905	0.512835
YER074W	RPS24A	0.006499	-0.719377	-0.347083	0.512531
YER058W	PET117	0.322495	0.027848	0.19313	0.509352
YJL190C	RPS22A	0.096328	-0.993173	-0.461434	0.507972
YBL072C	RPS8A	0.145677	-0.712775	-0.239942	0.505028
YLR264W	RPS28B	0.018802	-0.821964	-0.245131	0.504382
YBR191W	RPL21A	0.118651	-0.861067	-0.403355	0.504293
YBL087C	RPL23A	0.029866	-0.831431	-0.23714	0.50283
YBR071W	YBR071W	0.148488	0.113222	0.445527	0.495155
YPL143W	RPL33A	-0.035359	-0.937086	-0.407104	0.493553
YPL198W	RPL7B	0.08718	-0.849378	-0.203931	0.491217

YER131W	RPS26B	0.07043	-0.725674	-0.117067	0.491196
YGL068W	YGL068W	0.378093	-0.186224	-0.001101	0.483462
YDR450W	RPS18A	0.010412	-0.84183	-0.26668	0.482657
YGL076C	RPL7A	0.103547	-0.859001	-0.189162	0.482097
YDL213C	YDL213C	0.123549	-0.807568	-0.404484	0.48058
YGL103W	RPL28	-0.01346	-0.559886	-0.061391	0.480058
YDR418W	RPL12B	0.091571	-0.999659	-0.503767	0.479573
YER056C-A	RPL34A	-0.007775	-0.746713	-0.374977	0.475857
YMR142C	RPL13B	0.127857	-0.572353	-0.250679	0.475665
YGR269W	YGR269W	0.055182	0.061821	0.030552	0.473666
YOL127W	RPL25	0.069203	-0.59445	-0.229492	0.47215
YPL131W	RPL5	0.08545	-0.845368	-0.564583	0.471607
YIL070C	MAM33	0.25625	-0.191574	0.032859	0.469288
YOR234C	RPL33B	0.061538	-0.875326	-0.411851	0.467382
YOR020C	HSP10	0.448515	0.025606	0.200827	0.463932
YHR010W	RPL27A	0.108839	-0.816255	-0.412844	0.453166
YDR025W	RPS11A	0.136281	-0.696404	-0.294242	0.451136
YJL180C	ATP12	0.245961	-0.141687	0.113471	0.45012
YDL165W	CDC36	0.107551	-0.472556	-0.041449	0.448653
YBL074C	AAR2	0.267634	-0.040354	-0.048666	0.448
YGR109C	CLB6	-0.000037	-0.113577	-0.055661	0.447643
YLR344W	RPL26A	0.047977	-0.879122	-0.540513	0.447175
YNL069C	RPL16B	0.139347	-0.822804	-0.398497	0.440531
YNR037C	RSM19	0.268698	-0.009607	0.186422	0.437527
YLR333C	RPS25B	-0.016033	-0.449968	0.071861	0.435642
YLR048W	RPS0B	0.150414	-0.76686	-0.362619	0.429804
YDR064W	RPS13	0.065318	-0.840874	-0.318186	0.427738
YKR085C	MRPL20	0.216175	-0.19336	-0.07431	0.42716
YHR141C	RPL42B	0.041725	-0.819581	-0.362258	0.426889
YML009C	MRPL39	0.155218	-0.109321	0.142424	0.425152
YAL003W	EFB1	0.194839	-0.602084	-0.092383	0.42319
YMR143W	RPS16A	-0.056664	-0.728564	-0.348894	0.422739
YER117W	RPL23B	0.008764	-0.708365	-0.229027	0.420713
YGR140W	CBF2	0.125076	-0.040349	-0.242574	0.419817
YPL072W	UBP16	0.264867	-0.254217	-0.00193	0.419687
YJL191W	RPS14B	0.003183	-0.599291	-0.134721	0.417614
YJR048W	CYC1	0.157369	-0.121464	0.132928	0.416884
YBL027W	RPL19B	-0.007529	-0.788134	-0.375939	0.416712
YNL162W	RPL42A	-0.026306	-0.875223	-0.488386	0.416163
YML063W	RPS1B	-0.143355	-0.630945	-0.518988	0.415906
YNL067W	RPL9B	0.166273	-0.945577	-0.491205	0.411194
YDR296W	MHR1	0.178427	-0.011372	0.195241	0.410293
YDR543C	YDR543C	0.082736	0.277587	-0.04043	0.408006
YOR075W	UFE1	-0.184455	-0.573636	-0.414734	0.407202
YOR096W	RPS7A	0.133785	-0.892534	-0.513707	0.405812
YOL071W	YOL071W	0.112571	0.119053	0.379691	0.404289
YGR215W	RSM27	0.044595	-0.519086	-0.11186	0.400247
YJL189W	RPL39	-0.015612	-0.635927	-0.27066	0.398495
YJR145C	RPS4A	0.107158	-0.695092	-0.358523	0.39833
YDR382W	RPP2B	0.051756	-0.678156	-0.225859	0.397804
YIL133C	RPL16A	0.071306	-0.735202	-0.415247	0.391845
YOL121C	RPS19A	0.022221	-0.756993	-0.356947	0.391486

YGL030W	RPL30	0.019324	-0.86166	-0.378219	0.389675
YOR369C	RPS12	0.065293	-0.737048	-0.375792	0.388255
YJR123W	RPS5	0.103934	-0.788595	-0.349611	0.38763
YJL177W	RPL17B	0.011735	-0.735171	-0.492857	0.383436
YKR094C	RPL40B	0.025802	-0.776924	-0.368655	0.380461
YLR075W	RPL10	0.0884	-0.593946	-0.142837	0.376675
YLR340W	RPP0	0.319787	-0.587206	-0.27969	0.37464
YDR083W	YDR083W	0.009287	-0.203295	-0.327669	0.371954
YLR314C	CDC3	0.30353	-0.143887	-0.02349	0.370649
YLR150W	STM1	0.106328	-0.555058	-0.12851	0.368421
YHR203C	RPS4B	0.192487	-0.675982	-0.393547	0.365001
YML073C	RPL6A	0.072787	-0.897494	-0.445531	0.364937
YLR287C-A	RPS30A	0.027249	-0.417828	-0.149634	0.359564
YPR133W-A	TOM5	0.251198	-0.045717	0.123676	0.358756
YOL007C	CSI2	0.051724	-0.167239	0.199699	0.355542
YPL197C	YPL197C	-0.038737	-0.438157	-0.481658	0.353701
YHL001W	RPL14B	0.096557	-0.778416	-0.391164	0.350893
YCR031C	RPS14A	-0.006188	-0.431314	-0.11871	0.350842
YNR072W	HXT17	-0.008454	-0.255355	-0.200526	0.349399
YNL284C	MRPL10	0.228069	-0.069465	0.073151	0.348994
YDR041W	RSM10	0.201246	0.012143	0.178776	0.348736
YBR037C	SCO1	0.235773	0.063403	0.176845	0.348494
YMR116C	ASC1	0.122421	-0.391099	-0.025044	0.348045
YJR113C	RSM7	0.318058	-0.023773	0.031757	0.347527
YMR016C	SOK2	-0.127709	-0.318201	-0.302065	0.346888
YOR348C	PUT4	-0.167123	0.162414	0.554102	0.345096
YMR242C	RPL20A	0.090133	-0.871483	-0.68853	0.341661
YIL148W	RPL40A	0.082465	-0.81301	-0.30959	0.341504
YBR069C	TAT1	0.278525	-0.799566	-0.590832	0.340058
YML026C	RPS18B	-0.062963	-0.701819	-0.26306	0.339834
YOR150W	MRPL23	0.237537	-0.039937	0.174598	0.339406
YMR229C	RRP5	0.233217	-0.070034	-0.231	0.338826
YMR214W	SCJ1	0.437664	-0.273616	-0.485809	0.338039
YDL045W-A	MRP10	0.182917	-0.147475	0.07375	0.337685
YBL092W	RPL32	0.006945	-0.518611	-0.20035	0.336843
YLR341W	YLR341W	0.349208	-0.15467	-0.194419	0.335291
YGR118W	RPS23A	0.137047	-0.553901	-0.24862	0.334289
YDL227C	HO	0.098589	-0.336386	-0.150648	0.33416
YNL184C	YNL184C	0.123607	-0.276488	-0.168146	0.333158
YLR448W	RPL6B	0.067997	-0.778936	-0.484395	0.332598
YOR312C	RPL20B	0.091906	-0.796262	-0.539579	0.331968
YNL289W	PCL1	0.057759	-0.089965	0.274086	0.331473
YJR034W	PET191	0.101445	-0.037692	0.149262	0.331199
YJR118C	ILM1	0.100918	-0.305055	-0.034751	0.330807
YCR046C	IMG1	0.160521	-0.08112	0.15996	0.328754
YNL185C	MRPL19	0.144837	-0.269724	-0.129212	0.328244
YDR042C	YDR042C	0.246441	-0.060201	0.02044	0.326342
YOL026C	YOL026C	0.103006	-0.022239	0.133593	0.324703
YKL156W	RPS27A	-0.04171	-0.840553	-0.483093	0.323653
YKR044W	YKR044W	0.07847	-0.457896	-0.239441	0.323244
YCL044C	YCL044C	0.317972	0.023433	0.106438	0.322095
YMR011W	HXT2	-0.054782	-0.372496	-0.260101	0.321224

YKL147C	YKL147C	0.025392	-0.46945	-0.419937	0.320896
YGL135W	RPL1B	0.116926	-0.708741	-0.334083	0.320189
YLR441C	RPS1A	0.126512	-0.723488	-0.346456	0.318908
YJL115W	ASF1	0.070952	-0.166758	-0.125552	0.315974
YDR115W	YDR115W	0.143769	-0.176814	0.009039	0.314334
YDL215C	GDH2	0.122228	-0.467078	-0.532015	0.313371
YGL123W	RPS2	0.133507	-0.654366	-0.281036	0.313174
YKL138C	MRPL31	0.311882	-0.171956	-0.120566	0.31147
YLR294C	YLR294C	0.245148	-0.058729	0.044427	0.310647
YDR493W	YDR493W	0.130663	-0.100558	0.02929	0.310084
YJR144W	MGM101	0.163354	-0.28432	-0.063017	0.310004
YGR150C	YGR150C	-0.044816	-0.471837	-0.23826	0.309669
YLR295C	ATP14	0.184917	-0.152076	0.068293	0.309063
YGR027C	RPS25A	-0.014337	-0.170643	0.045971	0.307441
YKL180W	RPL17A	0.091247	-0.642694	-0.520296	0.305242
YDR430C	YDR430C	0.153159	-0.06035	0.029248	0.304947
YDL192W	ARF1	0.149555	-0.203086	-0.043719	0.304866
YNL101W	YNL101W	-0.000793	-0.350942	-0.416457	0.30457
YLR009W	YLR009W	0.089153	-0.896461	-0.897926	0.304569
YKL137W	YKL137W	0.164654	-0.050867	0.170845	0.304354
YDR454C	GUK1	0.04044	-0.428154	-0.045901	0.304121
YMR024W	MRPL3	0.191583	-0.068648	0.093933	0.302783
YOL038W	PRE6	0.279455	-0.110405	0.127751	0.30109
YDR144C	MKC7	0.057757	-0.330507	-0.235151	0.299253
YOR271C	YOR271C	0.18498	-0.447143	-0.375988	0.298949
YHL011C	PRS3	-0.197608	-0.384917	-0.460902	0.298342
YPR132W	RPS23B	0.063253	-0.381452	-0.135124	0.298102
YGR262C	YGR262C	-0.137775	-0.541703	-0.561277	0.297623
YDL158C	YDL158C	0.07364	-0.526513	-0.160942	0.296222
YNR046W	YNR046W	0.136752	-0.442594	-0.377622	0.291605
YPL118W	MRP51	0.361456	0.083359	0.111374	0.291206
YKR002W	PAP1	0.170523	-0.515405	-0.331732	0.289401
YGR085C	RPL11B	0.015634	-0.810906	-0.516516	0.28932
YBR088C	POL30	-0.02237	-0.455733	-0.037256	0.288631
YHR064C	PDR13	0.316229	-0.347575	-0.170608	0.287656
YJL063C	MRPL8	0.36543	-0.102117	-0.006162	0.285155
YBR084C-A	RPL19A	0.116786	-0.75536	-0.380454	0.285104
YGR285C	ZUO1	0.451017	-0.096121	-0.100758	0.283333
YLR062C	YLR062C	0.18771	0.139124	0.378738	0.281656
YPR191W	QCR2	0.050278	-0.305188	-0.058669	0.281234
YOL037C	YOL037C	0.123867	-0.421331	-0.207356	0.280642
YMR163C	YMR163C	-0.203717	-0.724047	-0.586714	0.279814
YAL012W	CYS3	0.330341	-0.190184	-0.222739	0.279027
YHR038W	FIL1	-0.111302	-0.692472	-0.49504	0.277292
YMR194W	RPL36A	0.059964	-0.371214	-0.219253	0.275108
YOR232W	MGE1	0.079525	-0.376316	-0.197502	0.274936
YHR154W	ESC4	0.066927	-0.307735	-0.048309	0.26591
YLL061W	MMP1	0.151475	-0.338459	-0.514818	0.259984
YOR182C	RPS30B	0.003795	-0.335365	-0.089595	0.258502
YML125C	YML125C	0.191121	-0.391028	-0.277082	0.258454
YLR076C	YLR076C	0.003534	-0.39654	-0.390631	0.257067
YOR375C	GDH1	0.316602	0.900707	0.953251	0.256995

YLR260W	LCB5	0.003302	-0.449184	-0.147035	0.254416
YCR034W	FEN1	0.122714	-0.603834	-0.221359	0.252584
YOR206W	YOR206W	0.116805	-0.716436	-0.724558	0.250902
YMR127C	SAS2	0.077468	-0.375374	-0.174705	0.248557
YBR208C	DUR1	0.272125	0.811169	0.888296	0.248365
YGR286C	BIO2	-0.196166	-0.364864	-0.419241	0.247711
YDR152W	YDR152W	0.116039	-0.517744	-0.512995	0.247622
YMR048W	YMR048W	0.064527	0.435867	0.488834	0.244372
YDL050C	YDL050C	0.62047	-0.337317	-0.470903	0.243245
YKL099C	YKL099C	0.158988	-0.640537	-0.670073	0.240991
YLR012C	YLR012C	-0.03516	-0.447388	-0.409751	0.240581
YLR167W	RPS31	0.043671	-0.680307	-0.309192	0.236729
YAL007C	ERP2	0.084903	-0.310898	0.050946	0.234841
YIL052C	RPL34B	0.129501	-0.460747	-0.325635	0.234638
YNL209W	SSB2	0.144052	-0.620885	-0.568785	0.233717
YNR015W	SMM1	0.189777	-0.278583	-0.380235	0.233116
YLR388W	RPS29A	-0.016469	-0.759274	-0.539213	0.232007
YKL028W	TFA1	-0.126092	-0.32786	-0.245209	0.231782
YOR387C	YOR387C	-0.072225	-0.308368	-0.271527	0.231257
YDR511W	YDR511W	-0.051379	-0.019195	0.31789	0.231083
YDR045C	RPC11	0.025947	-0.492835	-0.292495	0.227645
YER135C	YER135C	-0.037483	-0.503904	-0.360734	0.225857
YOR276W	CAF20	0.098917	-0.405102	-0.197213	0.225087
YAL025C	MAK16	-0.05374	-0.498551	-0.568873	0.224956
YPR163C	TIF3	0.250927	-0.445545	-0.32889	0.224616
YPR127W	YPR127W	0.100714	-0.267595	-0.509838	0.22415
YLR197W	SIK1	0.166246	-1.133194	-0.985726	0.222496
YNL149C	YNL149C	0.052647	-0.439846	-0.264053	0.221334
YDL121C	YDL121C	0.252877	-0.603091	-0.763544	0.221062
YDL007W	RPT2	0.038398	-0.3651	-0.205005	0.221001
YGR055W	MUP1	-0.002206	-0.658149	-0.502852	0.219673
YBR025C	YBR025C	0.228191	-0.896956	-0.663392	0.219598
YOR310C	NOP58	0.067066	-1.006705	-0.887036	0.219049
YLR106C	YLR106C	0.202321	-0.60223	-0.486283	0.217024
YKR001C	VPS1	0.30232	-0.089257	-0.039236	0.216725
YMR260C	TIF11	0.106615	-0.441908	-0.269119	0.215526
YJL050W	MTR4	0.038084	-0.572647	-0.699607	0.213019
YML056C	IMD4	0.096206	-0.637587	-0.48429	0.212496
YOR004W	YOR004W	0.043186	-0.648144	-0.838249	0.212211
YDL064W	UBC9	0.115227	-0.446593	-0.193379	0.211891
YOR129C	YOR129C	-0.076225	-0.351307	-0.163827	0.210351
YDR023W	SES1	0.273952	-0.431891	-0.277596	0.209155
YNL061W	NOP2	0.174424	-0.799678	-0.962052	0.207755
YDR429C	TIF35	0.116387	-0.39362	-0.353737	0.206742
YNL081C	YNL081C	0.051803	-0.331167	-0.078731	0.206333
YGR234W	YHB1	-0.005929	-0.397962	-0.022508	0.205847
YNL096C	RPS7B	0.07628	-0.780368	-0.616277	0.204671
YOR309C	YOR309C	0.13461	-0.966707	-0.887109	0.202579
YHR076W	YHR076W	0.310525	0.116426	0.103481	0.202439
YBR181C	RPS6B	0.059429	-0.749468	-0.431708	0.201896
YDR031W	YDR031W	-0.020497	0.141922	0.327778	0.201473
YFL011W	HXT10	0.012743	-0.428164	-0.210949	0.201207

YIL064W	YIL064W	0.039198	-0.454287	-0.613331	0.200624
YDR101C	YDR101C	0.153247	-0.624024	-0.925738	0.200512
YPL040C	ISM1	0.015769	-0.483214	-0.406087	0.200067
YMR087W	YMR087W	0.013505	0.00607	0.322573	0.199842
YIL131C	FKH1	-0.161575	-0.314088	-0.336504	0.199293
YPL226W	NEW1	0.006475	-0.515866	-0.362621	0.19751
YCR087C-A	YCR087C-A	0.094655	-0.47145	-0.712595	0.197226
YLR073C	YLR073C	-0.042347	-0.416291	-0.313592	0.195271
YDL229W	SSB1	0.209662	-0.642496	-0.617034	0.195167
YMR286W	MRPL33	-0.007897	-0.306456	-0.074951	0.194056
YKR039W	GAP1	0.244137	1.248753	1.40134	0.193433
YGR141W	YGR141W	-0.07124	-0.45351	-0.267105	0.192567
YAR075W	YAR075W	0.148853	-0.551739	-0.454971	0.192555
YMR044W	IOC4	-0.042038	-0.44201	-0.345711	0.192369
YML115C	VAN1	-0.071663	-0.335109	-0.174074	0.192192
YHL033C	RPL8A	0.088553	-0.500403	-0.286989	0.191937
YER007C-A	YER007C-A	0.039442	-0.422674	-0.2436	0.191167
YDR156W	RPA14	0.081208	-0.400647	-0.41783	0.189845
YER156C	YER156C	0.069242	-0.368306	-0.238741	0.18975
YLR449W	FPR4	-0.090742	-0.311563	-0.570129	0.189396
YFR035C	YFR035C	0.042618	-0.419903	-0.187961	0.188988
YHR062C	RPP1	0.111383	-0.482641	-0.350603	0.188807
YLL008W	DRS1	-0.063925	-0.357676	-0.610661	0.188553
YLL039C	UBI4	-0.022058	-0.330474	-0.048311	0.187978
YHR170W	NMD3	0.058811	-0.728308	-0.914111	0.187319
YPL090C	RPS6A	0.080121	-0.782809	-0.551886	0.187097
YOR063W	RPL3	0.140805	-0.835126	-0.641015	0.186333
YLR198C	YLR198C	0.523192	-0.550545	-0.787756	0.184753
YHR143W-A	RPC10	0.074652	-0.403848	-0.181777	0.184101
YOR210W	RPB10	0.001617	-0.399386	-0.340635	0.184009
YOL093W	YOL093W	-0.02102	-0.273048	-0.350588	0.183977
YHR081W	YHR081W	0.04946	-0.59362	-0.482905	0.182488
YOL048C	YOL048C	-0.109195	-0.434409	-0.272371	0.182413
YBR190W	YBR190W	-0.038604	-0.274697	-0.351361	0.1822
YPL245W	YPL245W	0.082019	-0.383039	-0.256918	0.180735
YDL194W	SNF3	-0.040396	-0.352356	-0.144423	0.179857
YOL144W	NOP8	0.08421	-0.574851	-0.848851	0.179696
YLR029C	RPL15A	0.068443	-0.745484	-0.554049	0.179548
YGR103W	YGR103W	0.339869	-0.851203	-1.106082	0.17746
YEL026W	SNU13	0.014248	-0.95802	-0.818091	0.175848
YHR072W-A	NOP10	0.031942	-0.589388	-0.413186	0.175799
YKL153W	YKL153W	-0.086688	-0.501217	-0.304393	0.175496
YLR222C	YLR222C	0.063976	-0.625198	-0.83588	0.175158
YGL070C	RPB9	0.014548	-0.335818	-0.094564	0.174969
YML006C	GIS4	-0.048682	-0.386853	-0.308962	0.173883
YGL054C	ERV14	0.05839	-0.400667	-0.070127	0.172083
YER002W	YER002W	0.076797	-0.429621	-0.463402	0.171263
YLL027W	ISA1	0.034432	-0.41689	-0.164635	0.171161
YEL024W	RIP1	0.059161	0.11863	0.357457	0.169008
YPR103W	PRE2	0.133715	-0.30907	-0.122348	0.168399
YJR002W	MPP10	0.005424	-0.499906	-0.447792	0.168329
YLR074C	BUD20	0.00925	-0.468741	-0.61729	0.167784

YHR065C	RRP3	0.034369	-0.378676	-0.472434	0.167168
YOR341W	RPA190	0.17792	-0.999312	-0.902052	0.167063
YCR072C	YCR072C	0.152303	-0.649544	-0.692999	0.166975
YBL073W	YBL073W	-0.059379	-0.389591	-0.309381	0.165696
YDR496C	YDR496C	0.196632	-0.448317	-0.364638	0.163612
YDR184C	ATC1	-0.051722	-0.421359	-0.425803	0.16314
YOR215C	YOR215C	0.052312	0.081571	0.38045	0.162656
YDR113C	PDS1	0.03867	-0.236537	-0.363036	0.160511
YEL042W	GDA1	0.142573	-0.397868	-0.261703	0.158887
YJR063W	RPA12	0.019208	-0.517043	-0.437928	0.158401
YLR175W	CBF5	0.04728	-0.589336	-0.682583	0.158206
YMR061W	RNA14	-0.061485	-0.4013	-0.347117	0.157921
YFR057W	YFR057W	0.12181	-0.312083	-0.009324	0.157831
YAR018C	KIN3	-0.083622	-0.333089	-0.250354	0.157334
YDL031W	DBP10	0.167596	-0.307795	-0.405831	0.15628
YJL193W	YJL193W	-0.069062	-0.519492	-0.217604	0.156014
YDR449C	YDR449C	-0.001329	-0.497969	-0.530328	0.155425
YGR255C	COQ6	0.014454	0.090424	0.355541	0.155273
YOR272W	YTM1	0.208482	-0.480046	-0.655915	0.15241
YHR052W	YHR052W	0.052944	-0.651573	-0.658392	0.152085
YPR190C	RPC82	0.075323	-0.353865	-0.364036	0.151628
YCR057C	PWP2	0.167197	-0.485622	-0.609501	0.151408
YOR340C	RPA43	0.081272	-1.213916	-1.096019	0.151122
YAL039C	CYC3	0.317728	0.221968	0.219458	0.150952
YPL232W	SSO1	0.053579	-0.367486	-0.272483	0.150389
YBR031W	RPL4A	0.099361	-0.63298	-0.404442	0.149764
YPR137W	RRP9	0.022934	-0.424515	-0.49213	0.149741
YMR014W	BUD22	-0.082123	-0.245866	-0.413708	0.149561
YDR046C	BAP3	0.015761	-0.417373	-0.295416	0.14918
YDL027C	YDL027C	0.009345	0.340471	0.417899	0.148942
YGR272C	YGR272C	0.057101	-0.64504	-0.619888	0.148806
YGR035C	YGR035C	0.115717	-0.370975	-0.52967	0.148618
YNL308C	KRI1	-0.106476	-0.141057	-0.385548	0.148574
YFL045C	SEC53	-0.012977	-0.310206	-0.042735	0.147951
YML093W	YML093W	0.156884	-0.791017	-0.734052	0.146942
YPL207W	YPL207W	0.128995	-0.511127	-0.466197	0.146889
YLR221C	YLR221C	0.160259	-0.509168	-0.528442	0.146499
YJR007W	SUI2	0.12091	-0.564047	-0.354119	0.14638
YPR084W	YPR084W	-0.029741	-0.37453	-0.260136	0.145834
YMR246W	FAA4	0.125134	-0.343448	-0.26212	0.14577
YPL211W	NIP7	0.077585	-0.857261	-0.903295	0.145734
YBR029C	CDS1	0.121379	-0.321875	-0.271015	0.144406
YDR020C	YDR020C	0.125087	-0.246067	-0.321369	0.143247
YDR374C	YDR374C	-0.013148	-0.464425	-0.120267	0.14285
YJL209W	CBP1	0.171733	-0.301751	-0.273274	0.142631
YLR172C	DPH5	0.145024	-0.256191	-0.320917	0.142431
YER047C	SAP1	-0.027072	-0.574316	-0.249786	0.141559
YLR129W	DIP2	0.098077	-0.553442	-0.336446	0.141538
YNL050C	YNL050C	0.122838	-0.311974	-0.196373	0.141043
YAL046C	YAL046C	-0.035108	0.124964	0.334888	0.13989
YHR193C	EGD2	0.078154	-0.36582	-0.256125	0.139869
YOR246C	YOR246C	0.043132	-0.303776	-0.018037	0.139389

YDR502C	SAM2	0.033303	-0.685403	-0.439778	0.139336
YLR196W	PWP1	0.170771	-0.55341	-0.813797	0.138917
YMR259C	YMR259C	-0.116215	-0.330739	-0.347587	0.138443
YLR121C	YPS3	0.251985	0.338732	0.355884	0.137874
YPL021W	ECM23	-0.083316	-0.799349	-0.824161	0.137477
YBR001C	NTH2	-0.013123	-0.537735	-0.236314	0.137458
YER006W	YER006W	0.124012	-0.795079	-0.674679	0.137018
YBR180W	DTR1	-0.025953	-0.596498	-0.399685	0.136711
YPL146C	YPL146C	-0.066015	-0.285625	-0.477962	0.136672
YER148W	SPT15	0.088076	-0.329243	-0.198455	0.135331
YDR377W	ATP17	0.171338	0.102145	0.340887	0.134684
YNL142W	MEP2	0.009878	1.478617	1.67432	0.134617
YER110C	KAP123	0.101082	-0.568059	-0.483229	0.134546
YLR186W	YLR186W	0.016386	-0.578348	-0.513575	0.134521
YGR123C	PPT1	-0.053102	-0.635823	-0.702366	0.134407
YMR114C	YMR114C	-0.072255	0.221935	-0.303211	0.13412
YNR033W	ABZ1	-0.02931	-0.522882	-0.473946	0.133875
YLR053C	YLR053C	-0.081482	0.172955	0.375095	0.133563
YHR088W	RPF1	0.08386	-0.374705	-0.368066	0.131556
YIL086C	YIL086C	0.162511	-0.169513	-0.312389	0.13097
YPL128C	TBF1	-0.095643	-0.322772	-0.193361	0.130954
YFR001W	LOC1	-0.055848	-0.510537	-0.439705	0.130175
YDL206W	YDL206W	0.150322	-0.322474	-0.131954	0.129907
YMR254C	YMR254C	0.024291	-0.421749	-0.403233	0.129779
YBR284W	YBR284W	-0.0926	-0.219274	-0.364401	0.129764
YGL122C	NAB2	-0.105066	-0.414878	-0.275332	0.128775
YEL054C	RPL12A	0.052128	-0.426261	-0.386525	0.128349
YIL085C	KTR7	0.150843	-0.187192	-0.30796	0.128323
YLR443W	ECM7	0.076965	-0.436236	-0.288182	0.128077
YLR446W	YLR446W	-0.123763	-0.294133	-0.336834	0.128049
YMR069W	YMR069W	-0.054988	-0.273147	-0.353939	0.128042
YGL029W	CGR1	-0.051848	-0.49017	-0.651883	0.127825
YGR145W	YGR145W	0.105166	-0.633978	-0.6838	0.127501
YCL054W	SPB1	0.116796	-0.399327	-0.584075	0.127465
YDR047W	HEM12	-0.041286	-0.32133	-0.165069	0.127139
YBL045C	COR1	0.18682	0.257155	0.303216	0.126831
YNL247W	YNL247W	0.266196	-0.31202	-0.306811	0.126787
YOR006C	YOR006C	-0.307162	NaN	0.8396	0.125902
YPL235W	RVB2	0.062735	-0.308976	-0.214521	0.125404
YLR288C	MEC3	-0.033948	-0.311963	-0.438191	0.124904
YFL001W	DEG1	-0.029958	-0.424984	-0.200159	0.124556
YMR310C	YMR310C	-0.001102	-0.462142	-0.542961	0.124472
YGL187C	COX4	-0.009647	0.055074	0.336282	0.123321
YKR059W	TIF1	0.146987	-0.46628	-0.202463	0.121894
YNL175C	NOP13	0.125553	-0.62074	-0.766021	0.121888
YFL022C	FRS2	0.10114	-0.376799	-0.231078	0.121782
YJR158W	HXT16	-0.058069	-0.224957	-0.370488	0.121334
YDR339C	YDR339C	-0.074095	-0.308558	-0.155438	0.120858
YPL093W	NOG1	0.052647	-0.635339	-0.693155	0.120615
YNL132W	YNL132W	0.208432	-0.482686	-0.598116	0.12039
YKL082C	YKL082C	0.072661	-0.444207	-0.556728	0.120352
YKL078W	YKL078W	0.046677	-0.781027	-0.878474	0.118702

YHL007C	STE20	0.092506	-0.169482	-0.310745	0.118428
YKL030W	YKL030W	-0.152398	0.335666	0.33492	0.11831
YBR121C	GRS1	0.158955	-0.378693	-0.263705	0.118
YLR005W	SSL1	0.001588	-0.359254	-0.08563	0.117907
YIR032C	DAL3	-0.108091	1.050645	1.274653	0.117101
YKR077W	YKR077W	-0.140291	-0.003252	0.309565	0.116856
YCR038C	BUD5	0.042341	-0.334741	-0.192631	0.116088
YGR228W	YGR228W	0.323623	-0.050535	-0.137235	0.116066
YLR244C	MAP1	0.121547	-0.483157	-0.298069	0.114425
YPR114W	YPR114W	0.047272	-0.392161	-0.160077	0.114088
YBL067C	UBP13	0.051253	-0.239207	-0.337562	0.11331
YLR093C	NYV1	0.004098	-0.608208	-0.351186	0.11285
YHR069C	RRP4	0.046768	-0.315842	-0.21018	0.112849
YFL029C	CAK1	-0.032024	0.345417	0.187833	0.111957
YOL041C	NOP12	0.115828	-0.561915	-0.582402	0.111174
YPR187W	RPO26	0.038643	-0.45579	-0.386453	0.110954
YPR002W	PDH1	-0.041182	0.569047	0.882931	0.110559
YPL244C	HUT1	0.007179	-0.368885	-0.345405	0.109837
YMR239C	RNT1	0.1177	-0.652258	-0.492016	0.109594
YJL220W	YJL220W	0.134937	-0.503808	-0.165148	0.109479
YBR067C	TIP1	0.549623	0.444629	0.347226	0.108262
YCR016W	YCR016W	0.108188	-0.371046	-0.41613	0.107894
YJL111W	CCT7	0.059406	-0.379879	-0.255833	0.107804
YNL313C	YNL313C	0.051564	-0.362567	-0.321933	0.107498
YMR017W	SPO20	-0.071318	-0.257174	-0.35632	0.107384
YIL103W	YIL103W	0.053949	-0.337502	-0.405577	0.107271
YMR231W	PEP5	-0.100485	-0.32794	-0.191658	0.10695
YLR017W	MEU1	0.109682	-0.432832	-0.348737	0.1068
YDR381W	YRA1	-0.153891	-0.482262	-0.160843	0.106246
YNL002C	RLP7	0.082922	-0.613653	-0.708374	0.106143
YCL049C	YCL049C	-0.064528	0.350475	0.571632	0.105634
YLR259C	HSP60	0.35875	0.004199	-0.071574	0.105464
YIL158W	YIL158W	-0.021922	-0.312034	-0.247146	0.105077
YOR315W	YOR315W	-0.121518	-0.438214	-0.214105	0.104945
YMR146C	TIF34	0.14178	-0.455564	-0.309699	0.104694
YDR361C	BCP1	0.080177	-0.539807	-0.473189	0.103868
YLR051C	YLR051C	0.096228	-0.559186	-0.418934	0.103398
YBR079C	RPG1	-0.005628	-0.33535	-0.402803	0.103365
YLR249W	YEF3	0.076766	-0.759542	-0.637098	0.102971
YPL086C	ELP3	0.159509	-0.417305	-0.592578	0.102428
YOR065W	CYT1	-0.028255	0.154633	0.406585	0.102345
YLR416C	YLR416C	-0.055409	-0.465469	-0.270336	0.102292
YBL044W	YBL044W	-0.113165	-0.321466	-0.189215	0.102255
YJR003C	YJR003C	0.03667	-0.357187	-0.313196	0.10136
YIL155C	GUT2	0.048199	0.219699	0.327796	0.101328
YPR080W	TEF1	0.146895	0.036006	0.310484	0.10109
YJL109C	YJL109C	0.092284	-0.643888	-0.634489	0.100974
YHR169W	DBP8	0.049372	-0.462337	-0.234613	0.099662
YIL009W	FAA3	-0.064162	-0.548196	-0.214851	0.099653
YKL050C	YKL050C	-0.021056	-0.462116	-0.526318	0.098969
YHR025W	THR1	0.36804	-0.093964	-0.120764	0.098953
YGL118C	YGL118C	-0.078348	-0.088853	-0.367316	0.098289

YLR002C	YLR002C	0.052618	-0.412963	-0.557502	0.098272
YOR095C	RKI1	-0.039383	-0.742973	-0.704388	0.097738
YCL031C	RRP7	0.063992	-0.325132	-0.282035	0.097721
YFL059W	SNZ3	-0.078617	-0.439261	-0.251253	0.0965
YLR060W	FRS1	0.045787	-0.317713	-0.247899	0.096117
YDR309C	GIC2	0.045101	0.173036	0.416024	0.095825
YHR196W	YHR196W	0.028358	-0.506738	-0.755934	0.095676
YIL061C	SNP1	0.014979	-0.345354	-0.199132	0.095635
YDR395W	SXM1	-0.002316	-0.265821	-0.337992	0.095331
YLR067C	PET309	-0.082315	-0.304007	-0.11428	0.095165
YAL033W	POP5	-0.058809	-0.326604	-0.19318	0.095132
YBL078C	AUT7	0.049119	0.572696	0.651989	0.095066
YDR529C	QCR7	0.105391	0.157315	0.314409	0.09488
YOL097C	WRS1	0.193725	-0.231147	-0.392301	0.094684
YMR290C	HAS1	0.055158	-0.760367	-0.848143	0.0942
YDR514C	YDR514C	-0.001348	-0.31506	-0.271733	0.092801
YDR513W	TTR1	-0.098009	0.019234	0.374482	0.091265
YLL011W	SOF1	0.099329	-0.567781	-0.559281	0.089554
YML028W	TSA1	0.045486	0.401709	0.473785	0.088954
YML022W	APT1	0.039877	-0.565564	-0.393978	0.088916
YNL113W	RPC19	-0.071132	-0.299309	-0.452529	0.088886
YAL030W	SNC1	-0.024379	0.091643	0.303777	0.088699
YIR026C	YVH1	0.155068	-0.72013	-0.668301	0.088042
YOR091W	YOR091W	0.092319	-0.45003	-0.565102	0.08743
YCR047C	BUD23	0.070634	-0.361142	-0.294105	0.087237
YIR004W	DJP1	-0.082001	-0.332499	-0.291725	0.086069
YGR200C	ELP2	0.100747	-0.431882	-0.537395	0.085908
YDR091C	RLI1	0.080615	-0.526038	-0.427953	0.085632
YDL060W	YDL060W	0.087084	-0.289366	-0.474214	0.084828
YPR126C	YPR126C	0.375377	0.290274	0.150311	0.084603
YDR012W	RPL4B	0.128754	-0.509906	-0.446425	0.084227
YOR001W	RRP6	0.029932	-0.391065	-0.361548	0.083716
YGR155W	CYS4	-0.010847	-0.406956	-0.278243	0.083706
YLL062C	MHT1	-0.09573	-0.309269	-0.141961	0.08357
YAR066W	YAR066W	0.058228	-0.349915	-0.169117	0.08342
YLL041C	SDH2	-0.138413	0.230032	0.387035	0.083079
YBL068W	PRS4	-0.013324	-0.567855	-0.443836	0.082993
YPL271W	ATP15	0.040252	0.222363	0.358494	0.081896
YOR295W	YOR295W	-0.013758	-0.330793	-0.267914	0.081854
YDL168W	SFA1	-0.125221	0.312544	0.417503	0.081824
YPL170W	YPL170W	-0.06104	0.116312	0.340087	0.081752
YKL054C	VID31	0.05072	-0.293943	-0.301163	0.08157
YAL019W	FUN30	0.108612	-0.238983	-0.396867	0.08126
YBR154C	RPB5	0.039828	-0.521128	-0.545331	0.081123
YCL036W	YCL036W	0.008914	-0.332243	-0.19839	0.08091
YDL228C	YDL228C	0.37592	-0.348606	-0.468911	0.08026
YCL059C	KRR1	0.044192	-0.250933	-0.417435	0.079087
YDL136W	RPL35B	0.195466	-0.371592	-0.489769	0.078982
YIL091C	YIL091C	0.067932	-0.369582	-0.650424	0.07895
YNL182C	YNL182C	0.033943	-0.572175	-0.475449	0.078804
YHL016C	DUR3	-0.023352	0.350536	0.674115	0.078332
YCR020W-B	HTL1	-0.066835	-0.144592	-0.538538	0.078322

YOL022C	YOL022C	0.112251	-0.383201	-0.342613	0.078148
YOR021C	YOR021C	0.08076	-0.373479	-0.423931	0.077924
YER106W	YER106W	0.033911	-0.055449	-0.311251	0.077711
YER122C	GLO3	-0.052301	-0.338896	-0.272863	0.077534
YPL043W	NOP4	0.037504	-0.426241	-0.634119	0.077194
YLR287C	YLR287C	0.01262	-0.39444	-0.396142	0.07714
YPR198W	SGE1	0.02611	0.224221	0.396829	0.077138
YJL011C	YJL011C	-0.00454	-0.331957	-0.251096	0.076009
YBL081W	YBL081W	-0.059276	-0.341506	-0.16403	0.074415
YOR359W	YOR359W	-0.029566	-0.746559	-0.375421	0.073867
YOR145C	YOR145C	0.037201	-0.408584	-0.413376	0.072567
YGL126W	SCS3	-0.198977	-0.369494	-0.354704	0.072277
YDR312W	SSF2	0.108474	-0.132704	-0.383777	0.071821
YOL142W	RRP40	0.023311	-0.326433	-0.452835	0.071805
YDL148C	YDL148C	-0.026846	-0.339793	-0.390149	0.071451
YGL034C	YGL034C	-0.246266	-0.096387	-0.313653	0.071303
YGR128C	YGR128C	0.039257	-0.602328	-0.746713	0.070541
YGR268C	YGR268C	0.174618	0.296825	0.333402	0.069696
YDR120C	TRM1	0.162467	-0.249758	-0.339343	0.069211
YNL151C	RPC31	0.032575	-0.249116	-0.416293	0.068929
YDR114C	YDR114C	-0.024239	-0.310895	-0.249057	0.06863
YNL062C	GCD10	-0.105464	-0.084616	-0.320663	0.06836
YOL115W	TRF4	-0.043067	-0.2451	-0.311529	0.068344
YKL216W	URA1	-0.092563	-0.737329	-0.642312	0.068264
YHR051W	COX6	0.020348	0.195076	0.341467	0.06781
YLL034C	YLL034C	0.013036	-0.222214	-0.387285	0.066882
YJL010C	YJL010C	-0.011752	-0.605294	-0.614229	0.066818
YML120C	NDI1	0.069417	0.192454	0.348521	0.066652
YHR148W	IMP3	0.091997	-0.397239	-0.735857	0.066557
YKL021C	MAK11	0.100466	-0.502499	-0.453299	0.066339
YIR036C	YIR036C	0.025208	0.29533	0.407188	0.065815
YKR081C	YKR081C	0.070774	-0.723923	-0.923213	0.065756
YNL075W	IMP4	0.086906	-0.433156	-0.640985	0.065678
YLR050C	YLR050C	-0.108712	0.206767	0.430573	0.0653
YLR090W	XDJ1	0.305585	0.208407	0.114164	0.065133
YOL012C	HTZ1	0.06931	-0.316499	-0.179588	0.065029
YPR010C	RPA135	0.129267	-0.845791	-0.989922	0.064725
YNL274C	YNL274C	-0.067801	-0.203645	-0.331754	0.063646
YHR085W	YHR085W	-0.026031	-0.277888	-0.419999	0.062947
YBR142W	MAK5	0.224278	-0.400733	-0.619576	0.062945
YNR070W	YNR070W	0.030761	-0.621242	-0.748862	0.062513
YAL036C	FUN11	0.160002	-0.640838	-0.669754	0.062378
YLR276C	DBP9	-0.041847	-0.826975	-0.556827	0.061766
YKL056C	YKL056C	0.085424	-0.573834	-0.2694	0.061528
YBR015C	MNN2	-0.045155	-0.333383	-0.08415	0.061248
YER126C	YER126C	-0.007318	-0.325844	-0.385057	0.06095
YAL008W	FUN14	-0.144369	0.096144	0.451708	0.060035
YCR079W	YCR079W	0.001292	0.241795	0.324635	0.059982
YPL237W	SUI3	0.182669	-0.392803	-0.348272	0.059952
YOR252W	YOR252W	0.07874	-0.462954	-0.284546	0.059862
YOR104W	YOR104W	0.064602	0.268438	0.339172	0.059354
YNL172W	APC1	0.051048	-0.435106	-0.189926	0.059168

YIR031C	DAL7	0.041782	0.702106	0.797589	0.058988
YPL028W	ERG10	-0.152697	-0.461958	-0.551118	0.05869
YML079W	YML079W	-0.07603	0.157872	0.340316	0.058398
YER064C	YER064C	-0.08742	-0.331656	-0.256193	0.058231
YLR409C	YLR409C	-0.049899	-0.485283	-0.334738	0.057998
YGR251W	YGR251W	-0.016572	-0.389248	-0.325448	0.057773
YGL246C	RAI1	0.079309	-0.342266	-0.302047	0.05731
YIR029W	DAL2	-0.021049	0.681033	0.88182	0.056873
YLR302C	YLR302C	0.017676	-0.034121	-0.340422	0.056686
YLR348C	DIC1	-0.296761	0.269554	0.778594	0.056439
YDL182W	LYS20	0.010167	-0.290544	-0.486779	0.055757
YNR038W	DBP6	0.054356	-0.374473	-0.393371	0.054828
YDR527W	YDR527W	0.076263	-0.360586	-0.27217	0.054434
YEL071W	DLD3	0.065558	0.680837	0.811025	0.053655
YPL266W	DIM1	0.109729	-0.375486	-0.535836	0.053358
YGR160W	YGR160W	0.205633	-0.176832	-0.323894	0.052308
YDL141W	BPL1	0.091666	-0.40056	-0.41686	0.052
YMR093W	YMR093W	0.042473	-0.576497	-0.596865	0.05168
YLR336C	SGD1	0.074011	-0.425534	-0.365894	0.051372
YHR068W	DYS1	0.188137	-0.378273	-0.314559	0.05062
YDL196W	YDL196W	-0.105044	-0.437103	-0.398946	0.049802
YJR044C	YJR044C	-0.044111	0.139671	0.354077	0.049423
YKL029C	MAE1	-0.03265	0.357583	0.562278	0.048661
YER082C	YER082C	0.097594	-0.213037	-0.310964	0.048627
YNL242W	SPO72	-0.055302	0.325465	0.233655	0.048544
YOR361C	PRT1	0.153203	-0.434408	-0.467612	0.048398
YBL062W	YBL062W	0.422289	0.298667	0.219501	0.048343
YFL021W	GAT1	-0.023661	0.369629	0.517325	0.048235
YDR399W	HPT1	-0.096001	-0.437809	-0.281053	0.047879
YOL080C	REX4	0.060609	-0.612118	-0.675657	0.047852
YEL056W	HAT2	0.065997	-0.572268	-0.340626	0.046418
YOL020W	TAT2	0.035542	-0.322712	-0.161284	0.046403
YJL201W	ECM25	-0.172212	-0.495096	-0.360388	0.045274
YPR061C	YPR061C	-0.057339	0.126342	0.335254	0.045106
YER109C	FLO8	0.032013	-0.070189	-0.362557	0.044378
YMR235C	RNA1	-0.022759	-0.224075	-0.342023	0.044354
YDR300C	PRO1	0.104509	-0.268975	-0.339037	0.044292
YOL077C	BRX1	0.10082	-0.493864	-0.688489	0.04381
YBL077W	YBL077W	0.531728	0.004553	-0.198765	0.04371
YKL014C	YKL014C	-0.061373	-0.564619	-0.317242	0.042444
YOR225W	YOR225W	0.169605	0.398251	-0.157684	0.042405
YGL078C	DBP3	-0.153467	0.071149	-0.444373	0.041705
YKL181W	PRS1	0.135127	-0.360171	-0.381103	0.0405
YDR465C	RMT2	0.165672	-0.200824	-0.430524	0.03964
YPL206C	YPL206C	-0.015086	0.192205	0.315789	0.039513
YOR152C	YOR152C	-0.140801	0.053712	0.314539	0.038873
YDR051C	YDR051C	-0.124057	-0.431358	-0.203424	0.038305
YDL063C	YDL063C	0.120011	-0.538075	-0.690895	0.03802
YPL134C	ODC1	0.037207	0.379313	0.425725	0.037095
YLR457C	NBP1	-0.057876	-0.447618	-0.243006	0.036999
YKL201C	MNN4	-0.001928	-0.030977	-0.325467	0.036875
YBR083W	TEC1	-0.053658	-0.229704	-0.359765	0.03601

YJR045C	SSC1	0.304242	0.257528	-0.014792	0.035854
YKR056W	RNC1	0.11001	-0.32334	-0.438817	0.035743
YKL192C	ACP1	0.114369	0.356946	0.228843	0.035391
YOR168W	GLN4	0.027446	-0.409498	-0.458291	0.035311
YPL265W	DIP5	-0.065585	0.371501	0.740946	0.034803
YLR323C	YLR323C	-0.029403	-0.339648	-0.156666	0.034769
YMR238W	DFG5	0.08193	0.28769	0.341386	0.034651
YMR279C	YMR279C	-0.066944	-0.221891	-0.341384	0.033709
YBR068C	BAP2	0.084132	-0.705573	-0.447981	0.033287
YKR013W	PRY2	0.072657	0.159371	0.310891	0.033276
YOL042W	NGL1	-0.043745	-0.351933	-0.292288	0.029672
YOR013W	YOR013W	0.024677	-0.410777	-0.223832	0.029553
YER154W	OXA1	0.026325	-0.43979	-0.247171	0.028585
YIL110W	YIL110W	0.038292	-0.405529	-0.321886	0.026783
YLR003C	YLR003C	0.080784	-0.258762	-0.470892	0.026541
YHR066W	SSF1	0.147259	-0.691477	-0.632814	0.026527
YFR055W	YFR055W	0.084994	0.279328	0.564018	0.026428
YOR171C	LCB4	-0.074664	-0.593858	-0.405388	0.026034
YLR263W	RED1	-0.045933	-0.321565	-0.036547	0.025653
YGR060W	ERG25	0.01018	0.2942	0.40353	0.025496
YGR283C	YGR283C	0.037111	-0.268647	-0.397154	0.024212
YNL115C	YNL115C	-0.047932	0.28404	0.429711	0.023607
YHR213W	YHR213W	-0.104577	0.311867	-0.009851	0.022596
YKL191W	DPH2	0.056028	-0.279431	-0.330576	0.022449
YOR220W	YOR220W	0.030693	0.219637	0.439967	0.022425
YGR280C	YGR280C	0.112789	-0.398649	-0.743401	0.022412
YPR144C	YPR144C	0.145913	-0.387477	-0.458021	0.021372
YER178W	PDA1	0.186453	0.360282	0.396013	0.021177
YPR016C	TIF6	-0.142025	-0.45455	-0.228867	0.019209
YGL009C	LEU1	-0.003825	-0.515821	-0.243429	0.019009
YDR324C	YDR324C	0.046976	-0.444058	-0.453336	0.018858
YNR002C	FUN34	0.168711	0.40598	0.389432	0.018694
YGL097W	SRM1	-0.107867	0.301905	0.071298	0.018215
YLR356W	YLR356W	0.000681	0.478847	0.664585	0.017861
YOR116C	RPO31	-0.020382	-0.375805	-0.319342	0.016914
YPL160W	CDC60	0.089472	-0.358373	-0.417243	0.016188
YOR208W	PTP2	-0.007252	0.329166	0.272717	0.015917
YOR388C	FDH1	-0.028942	0.164834	0.382804	0.015805
YGL099W	YGL099W	0.098031	-0.314865	-0.516134	0.015203
YGL120C	PRP43	0.163367	-0.591747	-0.793123	0.015067
YJL033W	HCA4	0.061508	-0.035544	-0.398312	0.013942
YKR079C	YKR079C	0.016159	-0.473676	-0.46517	0.013397
YBR143C	SUP45	0.078874	-0.506133	-0.328453	0.013386
YOR108W	YOR108W	0.000016	-0.273583	-1.37575	0.013236
YLR432W	IMD3	-0.013321	-0.30754	-0.422516	0.013154
YBR234C	ARC40	0.112289	0.278145	0.349582	0.013061
YPL136W	YPL136W	-0.033137	0.320348	0.246985	0.012797
YNL333W	SNZ2	-0.111769	-0.309245	-0.22566	0.012376
YBL024W	NCL1	0.078961	-0.34082	-0.350684	0.012259
YOR105W	YOR105W	-0.051002	0.127367	0.421006	0.012108
YLR176C	RFX1	-0.096865	-0.865895	-0.758336	0.011498
YGL050W	YGL050W	-0.074844	-0.322385	-0.027415	0.011479

YML108W	YML108W	-0.004685	-0.406535	-0.48643	0.010957
YPL012W	YPL012W	0.234196	-0.442358	-0.565902	0.010922
YHR138C	YHR138C	-0.105111	0.175797	0.386433	0.010425
YOR374W	ALD4	-0.089951	0.066247	0.326581	0.009509
YGR159C	NSR1	0.100332	-0.401069	-0.856662	0.007591
YCR065W	HCM1	-0.060961	-0.225987	-0.337747	0.007004
YGL253W	HXK2	-0.009522	-0.340965	-0.19685	0.006136
YMR134W	YMR134W	-0.304715	-0.017068	-0.288764	0.005667
YNL014W	YNL014W	-0.068454	-0.609783	-0.623234	0.005439
YNL141W	AAH1	-0.159465	-0.875406	-1.016444	0.003705
YMR280C	CAT8	-0.072366	-0.32831	-0.150816	0.003416
YMR053C	STB2	0.009186	0.381873	0.284584	0.003169
YMR304C-A	YMR304C-A	0.117556	0.542592	0.270871	0.00315
YPL217C	BMS1	0.140533	-0.375997	-0.423262	0.00292
YNL261W	ORC5	-0.05692	-0.186133	-0.334105	0.001129
YJR121W	ATP2	0.050966	0.390595	0.274058	0.001021
YLR272C	YCS4	-0.027721	-0.47768	-0.142037	0.000897
YHR144C	DCD1	-0.11954	-0.405643	-0.173571	0.00057
YGR090W	YGR090W	-0.113107	-0.420987	-0.339407	0.000032
YDL163W	YDL163W	-0.0372	0.075594	0.322205	-0.000226
YJL107C	YJL107C	0.123681	0.316609	0.128595	-0.000562
YKR019C	IRS4	0.025445	-0.302559	-0.236222	-0.000782
YOR287C	YOR287C	0.038229	-0.359474	-0.573713	-0.001493
YLR414C	YLR414C	0.06341	0.292476	0.333329	-0.002079
YLR401C	YLR401C	0.100014	-0.267287	-0.449022	-0.002099
YLR431C	YLR431C	-0.000223	-0.00591	0.361165	-0.002702
YGR081C	YGR081C	-0.065581	-0.478305	-0.494144	-0.003473
YJL128C	PBS2	0.044868	-0.118008	-0.336268	-0.003999
YJR030C	YJR030C	0.040322	0.302282	0.056055	-0.005206
YOR243C	YOR243C	0.058054	-0.190493	-0.370332	-0.005646
YML080W	YML080W	-0.006749	-0.431012	-0.36495	-0.006618
YAR073W	IMD1	-0.028708	-0.331021	-0.27578	-0.007103
YKL095W	YJU2	-0.20553	-0.038579	-0.416139	-0.007429
YPL142C	YPL142C	0.060483	-0.313798	-0.262695	-0.008939
YJR139C	HOM6	0.090907	0.31768	0.291771	-0.008987
YPR099C	YPR099C	0.057908	0.336008	-0.602427	-0.009359
YHR028C	DAP2	0.029807	0.249367	0.407281	-0.009887
YBR002C	RER2	-0.143124	-0.311536	-0.233231	-0.009986
YGR264C	MES1	0.129619	-0.428571	-0.395447	-0.01069
YOL131W	YOL131W	-0.109868	-0.061353	-0.328093	-0.01135
YLR155C	ASP3-1	0.222552	0.629603	0.370039	-0.011449
YOR142W	LSC1	-0.045846	0.103072	0.326101	-0.011701
YDL220C	CDC13	-0.296949	0.490894	0.752818	-0.011904
YIL079C	YIL079C	-0.020309	-0.125844	-0.522245	-0.012397
YCR007C	YCR007C	-0.135372	0.306515	0.432399	-0.012676
YNL106C	INP52	-0.018884	-0.400597	-0.679536	-0.013552
YDR284C	DPP1	-0.052287	0.150356	0.328747	-0.014144
YOL123W	HRP1	0.037425	-0.401699	-0.579546	-0.014153
YDR154C	YDR154C	0.055333	0.545159	0.318206	-0.014895
YGR161C	YGR161C	0.043669	0.464913	0.476231	-0.015097
YJL159W	HSP150	0.06862	0.592785	0.580618	-0.015479
YOR015W	YOR015W	0.236318	0.325511	0.057223	-0.015666

YOL124C	YOL124C	0.174675	-0.557026	-0.870211	-0.015805
YCL025C	AGP1	0.068386	0.882404	0.964348	-0.016054
YDL110C	YDL110C	-0.16396	0.328603	0.497607	-0.018775
YOR081C	YOR081C	-0.064904	-0.39472	-0.147684	-0.022481
YCL037C	SRO9	0.055549	-0.200883	-0.363024	-0.022497
YIL124W	AYR1	0.099985	0.311531	0.335469	-0.023683
YGL104C	YGL104C	-0.093713	0.345824	0.370408	-0.023894
YOR324C	YOR324C	0.052145	-0.315422	-0.133824	-0.024633
YDR087C	RRP1	0.059337	-0.14696	-0.387807	-0.025268
YGL245W	YGL245W	0.285073	0.372375	0.13639	-0.025323
YKL027W	YKL027W	-0.088719	-0.338541	-0.491505	-0.025578
YNL144C	YNL144C	-0.014525	0.435601	0.057982	-0.026144
YIL010W	DOT5	-0.088066	0.190916	0.328229	-0.026662
YIL050W	PCL7	-0.110945	0.238376	0.37703	-0.027777
YLR346C	YLR346C	-0.105476	-0.561538	-0.403761	-0.028035
YDL237W	YDL237W	0.016742	0.238844	0.362225	-0.028325
YGR038C-A	YGR038C-A	0.174336	0.428729	0.186973	-0.029426
YEL048C	YEL048C	-0.055308	-0.330998	-0.263101	-0.030887
YBR278W	DPB3	-0.047513	0.138517	0.357371	-0.031871
YJL148W	RPA34	0.063967	-0.979126	-0.849258	-0.0325
YNL255C	GIS2	0.079837	-0.642506	-0.451223	-0.032534
YER065C	ICL1	-0.425048	-0.023536	-0.405135	-0.032612
YOR111W	YOR111W	-0.204177	0.095462	0.307824	-0.03269
YLR267W	BOP2	-0.048901	0.399057	0.549524	-0.032944
YBR286W	APE3	0.207402	0.305476	0.225114	-0.032992
YNL305C	YNL305C	0.06622	0.412019	0.415122	-0.033015
YNL054W-A	YNL054W-A	0.149618	0.42364	0.248296	-0.033333
YMR046C	YMR046C	0.15302	0.429966	0.259564	-0.033438
YKR060W	YKR060W	0.033892	-0.403008	-0.418883	-0.03406
YMR291W	YMR291W	-0.092091	0.324127	-0.059584	-0.034925
YKR053C	YSR3	0.228507	0.558099	0.239837	-0.035388
YJR161C	COS5	0.057465	0.181414	0.318287	-0.03599
YHR020W	YHR020W	-0.038669	-0.375473	-0.557121	-0.036421
YAL061W	YAL061W	0.046694	-0.445698	-0.483447	-0.036784
YML067C	ERV41	-0.169312	0.157006	-0.305701	-0.037224
YBR012W-A	YBR012W-A	0.177739	0.418503	0.265243	-0.037224
YPL239W	YAR1	0.159307	-0.355625	-0.489318	-0.037753
YDL086W	YDL086W	0.195943	0.371059	0.341097	-0.037943
YOR051C	YOR051C	-0.012529	-0.310786	-0.344226	-0.038002
YPR001W	CIT3	-0.391695	0.462114	0.436756	-0.038751
YHR112C	YHR112C	0.090485	0.401676	0.437524	-0.038998
YLR299W	ECM38	-0.391977	0.427932	0.749265	-0.039531
YOL103W-A	YOL103W-A	0.160832	0.335438	0.222818	-0.040778
YKR067W	YKR067W	0.098106	0.321209	0.238183	-0.040795
YGR121C	MEP1	0.014619	0.239417	0.513824	-0.040969
YDR259C	YAP6	0.083954	0.496148	-0.084983	-0.041331
YLR216C	CPR6	0.39222	-0.034702	-0.213102	-0.042257
YMR031C	YMR031C	-0.14139	-0.057787	-0.32031	-0.043295
YGR209C	TRX2	-0.160693	0.388146	0.486286	-0.04459
YHR214C-C	YHR214C-C	0.119716	0.342343	0.215884	-0.04584
YDR060W	YDR060W	0.102024	-0.725068	-0.908741	-0.046104
YMR305C	SCW10	-0.032357	0.340965	0.27511	-0.046277

YKL172W	EBP2	-0.030381	-0.207579	-0.389165	-0.046498
YLR345W	YLR345W	-0.07835	0.170741	0.303223	-0.048111
YBR026C	MRF1'	-0.107796	0.294216	0.35682	-0.050441
YNL201C	YNL201C	-0.262338	0.350658	0.057677	-0.05068
YIR037W	HYR1	-0.077402	0.247101	0.363315	-0.05113
YOR342C	YOR342C	-0.066304	-0.322394	-0.619109	-0.052518
YJR137C	ECM17	-0.061375	0.307152	0.050294	-0.052902
YMR273C	ZDS1	0.073601	-0.316406	-0.557238	-0.052984
YER068W	MOT2	-0.16646	0.403127	0.06691	-0.053
YKR046C	YKR046C	-0.034229	0.670831	0.837382	-0.053648
YOR010C	TIR2	0.048676	0.354391	0.503568	-0.054013
YJL198W	YJL198W	0.166148	-0.334131	-0.431019	-0.054066
YNL284C-A	YNL284C-A	0.120843	0.505977	0.345701	-0.054156
YLR227W-A	YLR227W-A	0.153897	0.346876	0.214782	-0.054533
YMR319C	FET4	-0.174699	-0.317026	-0.227939	-0.055069
YKL080W	VMA5	0.178498	0.429739	0.162737	-0.055553
YOR289W	YOR289W	0.112537	0.290106	0.318779	-0.056807
YKL087C	CYT2	0.028369	0.35388	-0.095779	-0.05704
YOL122C	SMF1	0.026453	0.24102	0.368412	-0.057742
YBR239C	YBR239C	-0.233959	0.417725	0.039719	-0.057861
YLR256W-A	YLR256W-A	0.142613	0.389013	0.233923	-0.058285
YIL168W	SDL1	-0.234882	0.320099	0.243029	-0.058749
YHR049W	YHR049W	-0.02864	-0.336488	-0.330157	-0.058981
YMR191W	YMR191W	0.001689	0.33087	0.331775	-0.059153
YHR089C	GAR1	0.087405	-0.396543	-0.78599	-0.059596
YBR230C	YBR230C	-0.203093	0.090012	0.395443	-0.059644
YFR040W	SAP155	-0.125661	-0.273977	-0.329533	-0.059758
YDL112W	TRM3	0.09982	-0.296204	-0.464459	-0.06078
YJL199C	YJL199C	-0.048251	0.37541	0.526722	-0.060811
YOR155C	YOR155C	-0.087119	0.328321	0.501768	-0.061734
YMR088C	YMR088C	0.007561	0.420537	0.63766	-0.061758
YBR092C	PHO3	-0.212594	-0.899199	-0.571057	-0.062327
YDR365C	YDR365C	0.080107	-0.403073	-0.572883	-0.062615
YNL336W	COS1	-0.086156	0.191278	0.383145	-0.062829
YDR170W-A	YDR170W-A	0.053413	0.528363	0.182439	-0.063088
YJL052W	TDH1	0.178389	0.187622	0.410725	-0.063448
YEL046C	GLY1	-0.082996	-0.210503	-0.303567	-0.063516
YKR024C	DBP7	0.061492	-0.193786	-0.421411	-0.063616
YGR019W	UGA1	-0.191587	0.430298	0.741688	-0.064868
YLR281C	YLR281C	-0.079064	0.467755	0.28684	-0.06555
YOR036W	PEP12	0.015553	0.438858	0.247034	-0.066444
YJL075C	YJL075C	0.323366	-0.009199	-0.037639	-0.066651
YOL082W	YOL082W	-0.229354	0.264803	0.425256	-0.066664
YDR210W-C	YDR210W-C	0.15849	0.486517	0.27025	-0.06681
YBR302C	COS2	-0.008753	0.26883	0.367331	-0.066997
YPL110C	YPL110C	-0.062529	0.251504	0.413473	-0.067157
YIL051C	MMD1	-0.288368	0.430351	0.74291	-0.06733
YCL009C	ILV6	0.350656	0.337016	0.063905	-0.06895
YAR050W	FLO1	0.06278	0.333051	0.21256	-0.06999
YDR236C	FMN1	0.012976	0.212489	0.333529	-0.070012
YMR161W	HLJ1	-0.015395	0.284959	0.312939	-0.07006
YPL257W-A	YPL257W-A	0.13157	0.330838	0.213325	-0.070074

YDR021W	FAL1	0.152813	-0.308626	-0.632545	-0.070196
YPL158C	YPL158C	-0.160504	0.392745	0.079502	-0.071089
YHR008C	SOD2	0.037899	0.387429	0.394304	-0.071845
YER177W	BMH1	-0.07833	0.339209	0.18171	-0.072623
YER138W-A	YER138W-A	0.075802	0.671187	0.481818	-0.073257
YJL151C	YJL151C	-0.141635	0.108905	0.400563	-0.073987
YFR017C	YFR017C	-0.259104	0.389725	0.564494	-0.074602
YLR304C	ACO1	0.030358	0.548601	0.568647	-0.074651
YCL064C	CHA1	-0.379672	0.523505	0.904267	-0.07476
YJR098C	YJR098C	-0.409116	-0.069518	-0.280359	-0.075147
YJL068C	YJL068C	-0.021593	0.279326	0.376474	-0.075384
YPR030W	CSR2	-0.009295	0.303655	0.167336	-0.075719
YOR078W	BUD21	-0.019115	-0.392511	-0.634953	-0.076285
YBR128C	APG14	-0.03619	0.30652	0.176061	-0.077226
YLR361C	YLR361C	-0.138822	0.31684	0.064435	-0.077709
YNL064C	YDJ1	0.118194	-0.367351	-0.545756	-0.078353
YHR040W	YHR040W	-0.327325	0.147652	-0.240793	-0.078608
YLR270W	YLR270W	-0.118607	0.311585	0.503192	-0.078642
YPR158W-A	YPR158W-A	0.123447	0.339609	0.22518	-0.078815
YGR162W	TIF4631	0.029361	-0.410709	-0.561019	-0.078868
YKL120W	OAC1	-0.256595	-0.322572	-0.201039	-0.079222
YIR038C	GTT1	-0.064864	0.325123	0.389937	-0.079597
YGL162W	SUT1	-0.048685	-0.37553	-0.353368	-0.080243
YAL017W	FUN31	0.093488	0.247046	0.404782	-0.081097
YER138C	YER138C	0.112926	0.367934	0.319432	-0.0823
YPL004C	YPL004C	0.000719	0.089883	0.321836	-0.08239
YKL107W	YKL107W	0.021296	-0.234666	-0.326658	-0.082772
YBR137W	YBR137W	-0.21649	0.139561	0.413894	-0.082962
YGR237C	YGR237C	-0.167289	0.514825	-0.020365	-0.083635
YIL013C	PDR11	-0.136031	0.324817	0.214672	-0.084205
YNL071W	LAT1	0.130689	0.314877	0.286668	-0.084627
YGR189C	CRH1	0.045751	0.549142	0.418602	-0.084959
YER080W	YER080W	-0.134287	0.575993	0.107706	-0.086625
YNL174W	YNL174W	0.323553	-0.155323	-0.405834	-0.086893
YBR116C	YBR116C	-0.161016	0.305982	0.112041	-0.087095
YER011W	TIR1	-0.135738	0.456765	0.495573	-0.087335
YBR212W	NGR1	-0.000276	0.321848	0.212975	-0.088106
YER049W	YER049W	0.058261	-0.154916	-0.3124	-0.089395
YIL020C	HIS6	-0.002805	-0.293038	-0.332789	-0.089687
YDL014W	NOP1	-0.062546	-0.208014	-0.356629	-0.089875
YIL160C	POT1	-0.04807	0.544643	0.348564	-0.089985
YGR027W-B	YGR027W-B	0.112179	0.318543	0.287364	-0.090074
YMR262W	YMR262W	-0.099083	0.314129	0.355541	-0.090318
YGR211W	ZPR1	0.278913	-0.555108	-0.877439	-0.090797
YAL028W	YAL028W	-0.006669	0.308215	0.229349	-0.090946
YLR068W	YLR068W	-0.096314	-0.087441	-0.527156	-0.091008
YLL028W	TPO1	-0.22732	0.473083	0.095352	-0.092275
YBR005W	YBR005W	-0.123601	0.575361	0.204414	-0.092484
YPL205C	YPL205C	0.008268	0.511223	0.152725	-0.092574
YGL052W	YGL052W	-0.010065	0.215098	0.341283	-0.093906
YAL053W	YAL053W	0.162987	0.440992	0.41508	-0.094601
YNL217W	YNL217W	-0.393789	-0.295629	NaN	-0.094937

YAL054C	ACS1	-0.212612	0.313005	0.444941	-0.095962
YLR044C	PDC1	0.154055	0.38075	0.305608	-0.096035
YDR097C	MSH6	0.095144	0.359184	0.237598	-0.096074
YML111W	BUL2	0.042853	0.305382	0.075359	-0.096792
YER137C-A	YER137C-A	0.109672	0.486924	0.314225	-0.096903
YBL107W-A	YBL107W-A	0.073197	0.670632	0.43164	-0.097148
YHL028W	WSC4	-0.389093	-0.025797	0.250661	-0.098526
YPL123C	RNY1	-0.078958	0.418764	0.514071	-0.098575
YNL178W	RPS3	0.14059	-0.247999	-0.42132	-0.098642
YLR157C-A	YLR157C-A	0.114499	0.434468	0.206719	-0.098799
YML100W-A	YML100W-A	-0.015202	0.447299	0.221928	-0.098924
YHR189W	YHR189W	-0.019083	0.205347	0.394339	-0.099129
YMR050C	YMR050C	0.140661	0.389657	0.261041	-0.099327
YHR132W-A	YHR132W-A	-0.066443	-0.261383	-0.318427	-0.100384
YMR205C	PFK2	-0.13028	0.395985	0.193519	-0.101325
YDR516C	YDR516C	-0.336882	-0.061869	0.29133	-0.101527
YEL045C	YEL045C	-0.048719	-0.243848	-0.356211	-0.101849
YJL079C	PRY1	-0.189306	0.162896	0.360074	-0.102074
YER035W	EDC2	-0.084364	0.317612	0.298412	-0.102426
YCL017C	NFS1	0.199828	0.345549	0.201302	-0.102871
YML078W	CPR3	-0.146884	0.316816	0.222002	-0.103539
YBR052C	YBR052C	-0.092184	0.267332	0.309442	-0.103672
YMR051C	YMR051C	0.105892	0.482943	0.261159	-0.10481
YOR285W	YOR285W	-0.11546	0.392673	0.463759	-0.105155
YOR355W	GDS1	0.168421	-0.17941	-0.442311	-0.105514
YCL042W	YCL042W	-0.400807	0.499772	0.2095	-0.105841
YCL035C	GRX1	-0.07769	0.228478	0.301794	-0.106313
YPR137C-A	YPR137C-A	0.091265	0.47038	0.293933	-0.107081
YHR113W	YHR113W	0.040902	0.336639	0.410887	-0.108601
YHR159W	YHR159W	0.123211	0.304486	0.180638	-0.10864
YGL205W	POX1	0.037405	0.33874	0.017006	-0.108881
YBR221C	PDB1	0.191742	0.422462	0.337284	-0.110419
YPL126W	NAN1	0.150002	-0.688651	-0.715439	-0.110645
YER159C-A	YER159C-A	0.104922	0.475625	0.277579	-0.110689
YPR129W	SCD6	0.042736	0.343425	-0.052531	-0.110794
YIL034C	CAP2	0.02185	0.304051	0.376207	-0.110944
YHR032W	YHR032W	-0.091218	-0.125517	-0.661603	-0.111101
YDL067C	COX9	0.037199	0.40628	0.284968	-0.111196
YGR161W-B	YGR161W-B	0.110182	0.488615	0.337569	-0.111341
YDL072C	YDL072C	0.03064	0.500247	0.483667	-0.11137
YER160C	YER160C	0.095401	0.357737	0.278851	-0.111808
YLR328W	YLR328W	-0.150208	-0.304026	-0.230941	-0.112415
YLR257W	YLR257W	-0.179577	0.225037	0.343323	-0.112624
YIL107C	PFK26	-0.048376	0.325064	0.416219	-0.113337
YBL017C	PEP1	-0.044299	0.327195	0.152378	-0.113475
YMR251W-A	HOR7	-0.22438	0.825161	0.89159	-0.114224
YGR038C-B	YGR038C-B	0.100734	0.323174	0.251804	-0.114499
YDR261C-C	YDR261C-C	0.128752	0.466395	0.368428	-0.115217
YOR230W	WTM1	-0.083334	0.290459	0.376255	-0.11523
YPL006W	NCR1	-0.05342	0.363662	0.529133	-0.115583
YDL239C	YDL239C	-0.141956	0.584623	0.58057	-0.115935
YDR391C	YDR391C	-0.190929	0.290365	0.402408	-0.116113

YFL062W	COS4	-0.057927	0.31992	0.305067	-0.116523
YNL036W	NCE103	0.140422	0.385169	0.111891	-0.116704
YKR093W	PTR2	0.005908	0.481449	0.551242	-0.117479
YAR009C	YAR009C	0.135981	0.46544	0.385838	-0.117748
YPR158W-B	YPR158W-B	0.146589	0.404896	0.315096	-0.117828
YDR098C-A	YDR098C-A	0.096343	0.49201	0.31394	-0.118595
YOR012W	YOR012W	-0.10367	0.272004	0.307874	-0.119093
YGR161C-C	YGR161C-C	0.139908	0.516445	0.345499	-0.119307
YDL180W	YDL180W	-0.131749	0.403224	0.598144	-0.119666
YOR142W-B	YOR142W-B	0.130408	0.40112	0.296299	-0.120626
YGR161CD01	#N/A	0.109704	0.462471	0.370509	-0.121472
YNL054W-B	YNL054W-B	0.118263	0.350361	0.246279	-0.121983
YKL164C	PIR1	0.056575	0.571214	0.455083	-0.122362
YDR204W	COQ4	-0.001593	0.41867	0.471229	-0.123202
YOR343C-B	YOR343C-B	0.119846	0.353453	0.223981	-0.123326
YGR027W-A	YGR027W-A	0.101937	0.461299	0.311393	-0.123408
YOR221C	MCT1	-0.100214	0.344027	0.417635	-0.123662
YGL037C	PNC1	-0.322657	0.374092	0.572937	-0.123822
YPL191C	YPL191C	-0.00427	0.357572	0.294936	-0.124383
YJR026W	YJR026W	0.119241	0.520162	0.25951	-0.124521
YLR180W	SAM1	-0.044651	-0.684758	-0.688504	-0.124838
YKR058W	GLG1	-0.168535	0.3601	-0.005668	-0.125512
YOR140W	SFL1	-0.07871	0.391452	0.007501	-0.125866
YAR010C	YAR010C	0.140114	0.44656	0.326513	-0.125877
YDR261W-B	YDR261W-B	0.067878	0.303673	0.167473	-0.125934
YDR032C	PST2	-0.026041	0.464537	0.442805	-0.126233
YJR028W	YJR028W	0.117087	0.509122	0.247219	-0.126531
YOR035C	SHE4	-0.007762	0.382358	0.288727	-0.126628
YDR098C-B	YDR098C-B	0.109366	0.38056	0.296901	-0.12675
YNL259C	ATX1	-0.445049	-0.076873	0.262744	-0.126906
YMR284W	YKU70	-0.266705	0.180655	0.321362	-0.127012
YPL257W-B	YPL257W-B	0.091014	0.407083	0.339702	-0.12813
YFL010W-A	AUA1	-0.189426	0.366125	-0.158293	-0.129724
YAL015C	NTG1	0.125701	0.334616	0.144497	-0.130047
YNR071C	YNR071C	-0.190073	0.716279	0.217964	-0.130281
YDR001C	NTH1	-0.095624	0.540162	0.592776	-0.130477
YDR109C	YDR109C	-0.142249	0.45451	0.042593	-0.131167
YPL273W	SAM4	0.077154	-0.205889	-0.359869	-0.132107
YCR104W	PAU3	-0.069967	0.325655	0.242764	-0.133142
YMR039C	SUB1	-0.100074	-0.100494	-0.314217	-0.133296
YGR192C	TDH3	0.090974	0.103215	0.315148	-0.133927
YBR289W	SNF5	-0.158477	0.457938	0.032854	-0.134032
YML045W	YML045W	0.120523	0.399628	0.340196	-0.134313
YBR117C	TKL2	-0.320651	-0.021607	-0.083734	-0.13434
YML017W	PSP2	-0.09584	-0.533359	-0.772776	-0.134566
YGL204C	YGL204C	-0.342052	0.00583	0.23536	-0.13515
YJL060W	YJL060W	0.010492	0.331981	0.296689	-0.135197
YBL039C	URA7	0.074477	0.312889	0.164558	-0.135443
YNR007C	AUT1	-0.025051	0.432461	0.269793	-0.135663
YCR102C	YCR102C	-0.020893	0.45917	0.076038	-0.135868
YHR209W	YHR209W	-0.083513	0.407437	0.453942	-0.135915
YJL176C	SWI3	-0.006002	0.320673	0.053492	-0.136115

YNR053C	YNR053C	-0.178152	-0.870618	-0.953269	-0.136833
YLL020C	YLL020C	0.123289	0.296745	0.302104	-0.137061
YOR363C	PIP2	-0.059116	0.361475	0.162321	-0.137128
YDR365W-A	YDR365W-A	0.085614	0.358647	0.22749	-0.137687
YJR029W	YJR029W	0.09967	0.394941	0.313912	-0.138495
YFL002W-A	YFL002W-A	0.12629	0.334081	0.246893	-0.138702
YPR167C	MET16	-0.062711	0.356242	0.030068	-0.139103
YPR007C	SPO69	-0.185403	0.675511	0.347504	-0.139684
YLL037W	YLL037W	-0.028203	0.347395	0.089709	-0.140343
YML039W	YML039W	0.083017	0.407855	0.244885	-0.140705
YBR101C	YBR101C	0.143118	-0.080478	-0.370784	-0.140962
YPL194W	DDC1	-0.10349	0.644633	0.414374	-0.141699
YOR317W	FAA1	-0.034873	0.251566	0.318996	-0.142357
YBR301W	YBR301W	-0.139209	0.547172	0.224037	-0.142744
YER036C	YER036C	-0.060683	-0.438893	-0.461898	-0.142907
YNL208W	YNL208W	-0.057824	0.445793	0.341982	-0.142929
YPR077C	YPR077C	0.115194	0.220104	0.315619	-0.143499
YMR004W	MVP1	-0.164787	0.910308	0.360588	-0.143881
YGL056C	SDS23	-0.103443	0.357969	0.066912	-0.145057
YPR110C	RPC40	0.18511	-0.272817	-0.325826	-0.145382
YDR341C	YDR341C	0.111239	-0.202714	-0.350541	-0.145683
YOR003W	YSP3	-0.212986	0.406154	0.430336	-0.145972
YBR027C	YBR027C	-0.140264	-0.047161	-0.318488	-0.146682
YIL088C	YIL088C	0.033326	0.438326	0.562642	-0.14717
YEL063C	CAN1	-0.272358	0.539501	0.49603	-0.147602
YMR008C	PLB1	-0.117294	0.414596	0.44374	-0.148155
YDR365W-B	YDR365W-B	0.114194	0.480541	0.285917	-0.149941
YEL058W	PCM1	0.155025	0.343244	0.182833	-0.150544
YDR133C	YDR133C	-0.108574	0.332311	0.394503	-0.150991
YFR034C	PHO4	-0.055991	0.345514	0.042724	-0.151142
YOR192C-B	YOR192C-B	0.077498	0.489451	0.281094	-0.151377
YBL113C	YBL113C	-0.012553	0.317463	0.017349	-0.152938
YDR222W	YDR222W	0.035594	-0.157033	-0.35627	-0.153239
YDR481C	PHO8	-0.197658	0.121979	0.428414	-0.153903
YPL231W	FAS2	0.126087	0.393895	0.295924	-0.154011
YLR410W-B	YLR410W-B	0.035292	0.494512	0.248067	-0.154321
YAL060W	YAL060W	-0.312146	0.301542	0.621873	-0.154969
YHR214C-B	YHR214C-B	0.085685	0.452212	0.307469	-0.155034
YPR158CD01	#N/A	0.146548	0.524196	0.31297	-0.155564
YLR227W-B	YLR227W-B	0.0948	0.420404	0.298722	-0.156389
YDR358W	GGA1	-0.017138	0.432547	0.409272	-0.156419
YLR149C	YLR149C	-0.087833	0.31652	0.291054	-0.156497
YGR047C	TFC4	0.052267	0.305815	0.285163	-0.156667
YJL161W	YJL161W	-0.480156	0.689885	0.795974	-0.156781
YDR134C	YDR134C	-0.052296	0.369009	0.370832	-0.156902
YJL217W	YJL217W	-0.03413	0.272435	0.370066	-0.157081
YOR188W	MSB1	-0.025936	0.34613	0.054709	-0.157289
YBR139W	YBR139W	-0.152643	0.541265	0.601846	-0.157368
YJL171C	YJL171C	0.034276	0.415262	0.51092	-0.157575
YBR241C	YBR241C	0.02341	0.366391	0.398508	-0.157638
YER158C	YER158C	-0.289249	0.611234	0.218483	-0.157879
YGL101W	YGL101W	-0.078879	-0.326736	-0.228646	-0.158021

YLR110C	YLR110C	-0.050773	0.349895	0.434119	-0.158087
YPR194C	OPT2	-0.062168	0.572266	0.565028	-0.158411
YPL087W	YDC1	-0.04522	0.421628	0.464761	-0.158608
YGR008C	STF2	-0.199798	0.234474	0.402932	-0.158617
YJL213W	YJL213W	-0.215938	0.331419	0.220967	-0.159452
YOL100W	PKH2	0.016412	0.305993	0.16541	-0.15966
YNL168C	YNL168C	0.002527	0.352447	0.331078	-0.159882
YJR084W	YJR084W	-0.08938	0.665121	0.294465	-0.160959
YLR080W	YLR080W	-0.133017	0.346803	0.326617	-0.161175
YDR197W	CBS2	-0.022749	0.439217	0.222829	-0.161521
YMR135W-A	YMR135W-A	-0.287223	-0.317514	-0.131765	-0.161729
YKL096W-A	CWP2	0.038206	0.22627	0.580977	-0.161797
YDR388W	RVS167	-0.078684	0.361947	0.184597	-0.161837
YDL022W	GPD1	-0.482885	0.002208	0.495091	-0.162534
YHL045W	YHL045W	-0.065831	0.243247	0.402365	-0.162638
YOR092W	ECM3	-0.336578	0.137165	0.031301	-0.162988
YBR093C	PHO5	-0.3629	-0.531908	-0.369078	-0.163355
YJR016C	ILV3	-0.143972	-0.128985	-0.338932	-0.163357
YMR049C	ERB1	-0.197885	0.422893	0.083194	-0.163774
YMR321C	YMR321C	0.065356	-0.249114	-0.380109	-0.165005
YBL005W-B	YBL005W-B	0.068434	0.41538	0.247163	-0.165083
YML083C	YML083C	-0.183225	0.578105	0.118201	-0.166519
YGR070W	ROM1	-0.118868	0.446377	0.349164	-0.166711
YPR158C-C	YPR158C-C	0.118832	0.599612	0.307612	-0.167294
YMR181C	YMR181C	-0.1453	0.348354	0.202613	-0.167366
YGR175C	ERG1	-0.056302	0.359714	0.317414	-0.167542
YMR151W	YIM2	-0.006722	0.499009	0.403716	-0.167565
YLL031C	GPI13	-0.190826	0.457156	-0.076493	-0.168015
YLR464W	YLR464W	-0.209229	0.421256	-0.011264	-0.16876
YDR248C	YDR248C	-0.267655	0.203014	0.410859	-0.169389
YLR300W	EXG1	-0.122964	0.224033	0.318766	-0.16942
YHR030C	SLT2	-0.012127	0.45223	0.350654	-0.169824
YDR538W	PAD1	-0.183606	0.379651	0.108226	-0.169941
YBR156C	SLI15	0.034092	0.485243	0.213062	-0.169948
YIL067C	YIL067C	-0.301949	0.341922	0.196446	-0.169955
YMR045C	YMR045C	0.114084	0.370656	0.262819	-0.170011
YHR135C	YCK1	0.006612	0.305497	-0.003492	-0.170088
YHL021C	YHL021C	-0.244576	0.451894	0.661447	-0.170368
YDL238C	YDL238C	-0.035865	0.65016	0.496737	-0.170629
YDR009W	GAL3	0.006635	0.553757	0.326104	-0.171713
YDR316W-B	YDR316W-B	0.104039	0.413644	0.245122	-0.171969
YKR034W	DAL80	0.179235	1.049538	1.104084	-0.172456
YDR210WD01	#N/A	0.121377	0.539233	0.426099	-0.172805
YDR043C	NRG1	0.035543	0.36037	0.290729	-0.173686
YDR416W	SYF1	-0.02912	0.455352	0.227476	-0.173984
YJL163C	YJL163C	-0.109301	0.402652	0.435175	-0.174138
YOR322C	YOR322C	-0.183648	0.451386	0.136975	-0.174791
YMR164C	MSS11	-0.3329	0.233123	-0.197091	-0.175821
YAL062W	GDH3	-0.228107	0.432885	0.506433	-0.177246
YBR132C	AGP2	-0.202222	0.438852	0.484604	-0.177823
YEL059W	YEL059W	-0.01216	0.391231	0.084475	-0.178014
YBL001C	ECM15	-0.166123	0.256348	0.310021	-0.178851

YGR204W	ADE3	0.178651	0.45419	0.348877	-0.180152
YMR204C	YMR204C	-0.233452	0.482238	0.105577	-0.180274
YPL166W	YPL166W	-0.022993	0.523432	0.434278	-0.180375
YFR044C	YFR044C	0.143708	0.561003	0.363861	-0.182732
YER141W	COX15	-0.233592	0.700921	0.535674	-0.182927
YNL098C	RAS2	0.139743	-0.304343	0.08721	-0.183768
YJL069C	YJL069C	-0.193936	0.34975	-0.076909	-0.183907
YBL064C	YBL064C	-0.372236	0.324182	0.691182	-0.184163
YPL154C	PEP4	-0.04653	0.841395	0.873214	-0.184445
YMR250W	GAD1	-0.338132	0.437227	0.68919	-0.184595
YDL134C	PPH21	-0.087597	0.535651	0.626753	-0.185162
YBL048W	YBL048W	-0.034516	0.329867	0.186752	-0.186111
YEL073C	YEL073C	-0.011992	0.40126	0.166173	-0.186674
YCR005C	CIT2	0.00775	0.711442	0.713786	-0.1879
YOR264W	YOR264W	-0.385107	0.122555	-0.150477	-0.189016
YGR166W	KRE11	-0.010126	0.355627	0.230607	-0.18923
YPR128C	YPR128C	0.06871	0.337166	0.103562	-0.191181
YMR226C	YMR226C	0.088419	0.539402	0.318959	-0.191453
YIL117C	PRM5	0.199572	0.477969	0.413511	-0.192806
YAL044C	GCV3	-0.396254	0.463483	0.704468	-0.193484
YNR066C	YNR066C	0.061405	0.365061	0.14363	-0.194173
YML010C-B	YML010C-B	-0.021119	0.307472	0.147504	-0.195515
YHR070W	YHR070W	-0.114232	0.451533	-0.093885	-0.196021
YJL142C	YJL142C	-0.017812	0.36012	0.222241	-0.196207
YGL026C	TRP5	0.28925	0.504346	0.109972	-0.198254
YGR282C	BGL2	0.109288	0.551127	0.52757	-0.199484
YER053C	YER053C	-0.168759	0.582537	0.364071	-0.201446
YDR019C	GCV1	-0.422971	0.469179	0.825765	-0.201476
YKR091W	SRL3	-0.043791	0.886293	0.918773	-0.201709
YCR101C	YCR101C	0.092779	0.480447	0.137732	-0.201753
YDR058C	TGL2	-0.245692	0.281322	0.371406	-0.202315
YDR525W-A	YDR525W-A	-0.149962	0.375485	0.41172	-0.203434
YBL049W	YBL049W	-0.050927	0.362627	0.193165	-0.203508
YER115C	SPR6	0.021383	0.367293	0.144519	-0.204485
YNL068C	FKH2	-0.014388	0.354179	0.184241	-0.204803
YBL101W-B	YBL101W-B	0.065366	0.382425	0.231021	-0.204933
YER041W	YER041W	-0.1286	0.55813	0.432151	-0.205168
YHR208W	BAT1	0.024674	-0.279794	-0.433322	-0.205212
YPR076W	YPR076W	-0.13037	0.62699	0.320312	-0.205669
YCR052W	RSC6	-0.344657	0.206978	-0.04386	-0.205841
YNL173C	MDG1	-0.171706	0.312323	0.325602	-0.210945
YIL152W	YIL152W	0.076291	0.332488	0.133999	-0.214362
YML117W-A	YML117W-A	-0.044411	0.344062	0.241043	-0.21499
YMR174C	PAI3	-0.197196	0.40925	0.192943	-0.215333
YOL103W-B	YOL103W-B	0.103805	0.590035	0.373514	-0.215904
YCL040W	GLK1	-0.282656	0.486133	0.619606	-0.216486
YBR267W	YBR267W	-0.243704	0.684333	-0.051709	-0.217117
YDR055W	PST1	0.088491	0.449329	0.24159	-0.219137
YOR100C	CRC1	-0.011183	0.47327	0.257418	-0.22017
YLR027C	AAT2	0.101683	0.361955	0.173284	-0.220245
YIL025C	YIL025C	-0.116445	0.180753	0.313663	-0.22088
YOR383C	YOR383C	-0.124561	0.328858	0.094004	-0.221119

YGL059W	YGL059W	-0.06826	0.404905	0.075913	-0.221482
YLR342W	FKS1	0.138873	0.374969	0.289116	-0.222079
YPR098C	YPR098C	-0.147282	0.358208	0.165127	-0.222302
YIL167W	YIL167W	-0.332708	0.196854	0.038602	-0.222616
YOR053W	YOR053W	-0.117524	0.320771	0.218411	-0.22299
YJL072C	YJL072C	-0.113506	0.568574	0.247273	-0.225505
YLL025W	YLL025W	-0.139419	0.680722	0.191543	-0.22576
YDL178W	AIP2	-0.027745	0.374344	0.401182	-0.2264
YER121W	YER121W	-0.302866	0.243609	0.137747	-0.227721
YGR032W	GSC2	0.265638	0.574619	0.123917	-0.229624
YJR096W	YJR096W	-0.075439	0.438034	0.349009	-0.229997
YOR142W-A	YOR142W-A	0.107933	0.530618	0.287001	-0.232769
YCL019W	YCL019W	0.116815	0.382136	0.308385	-0.232984
YER067W	YER067W	-0.455758	0.107912	0.379006	-0.233571
YBL029W	YBL029W	-0.386931	-0.12067	0.000059	-0.233963
YPR002C-A	YPR002C-A	0.044377	0.403362	0.128429	-0.234962
YMR297W	PRC1	-0.005643	0.52935	0.454263	-0.237969
YOR273C	YOR273C	-0.30299	0.238355	0.429204	-0.238801
YJL018W	YJL018W	0.069775	0.392951	0.372672	-0.240218
YDL054C	YDL054C	0.254499	0.533453	0.182254	-0.240223
YEL017C-A	PMP2	-0.18332	0.333712	0.189265	-0.241445
YAR071W	PHO11	-0.486915	-0.388472	-0.207292	-0.241542
YPR055W	SEC8	-0.144616	0.740306	0.280857	-0.241767
YPL240C	HSP82	0.341214	0.140102	-0.255097	-0.242056
YOL058W	ARG1	0.523787	1.39019	0.845557	-0.243036
YFL039C	ACT1	0.114086	0.55685	0.344983	-0.243116
YDR171W	HSP42	-0.076278	0.393441	0.343483	-0.243625
YJL185C	YJL185C	-0.037875	0.401193	0.395332	-0.245247
YPR184W	GDB1	0.052374	0.455678	0.340721	-0.245478
YPR058W	YMC1	0.076399	0.34698	0.130931	-0.246553
YIL053W	RHR2	-0.15574	-0.406175	-0.410055	-0.247152
YGR203W	YGR203W	-0.049351	0.302796	0.199325	-0.247214
YBR293W	YBR293W	-0.14448	0.354325	0.239059	-0.247444
YNL055C	POR1	-0.139711	0.824113	0.727544	-0.24774
YLR372W	SUR4	-0.024742	0.468807	0.142439	-0.247901
YIL157C	YIL157C	-0.418169	0.417018	0.226859	-0.2505
YOL119C	YOL119C	0.10643	0.569568	0.45961	-0.251503
YGL186C	YGL186C	-0.028643	0.302148	0.178075	-0.251837
YIL136W	OM45	-0.124747	0.345559	0.297526	-0.253002
YKL161C	YKL161C	-0.089446	0.521031	0.260489	-0.254406
YJR027W	YJR027W	0.110264	0.752752	0.530798	-0.254943
YGR180C	RNR4	-0.109705	0.526516	0.290394	-0.255499
YDR243C	PRP28	0.061321	0.386308	-0.029063	-0.256772
YOR028C	CIN5	-0.184909	0.449597	0.138408	-0.258058
YOR027W	STI1	0.199653	-0.016425	-0.401624	-0.259559
YLR251W	YLR251W	-0.156792	0.316156	0.535764	-0.260271
YGR124W	ASN2	0.364988	0.587483	0.005068	-0.261161
YKR092C	SRP40	0.095811	-0.062946	-0.301313	-0.261385
YHR004C	NEM1	-0.168009	0.78083	0.330672	-0.261571
YMR189W	GCV2	-0.207162	0.599012	0.732495	-0.262679
YNL017C	YNL017C	-0.297028	0.804391	0.154096	-0.262753
YPR112C	MRD1	0.15872	-0.138709	-0.560011	-0.263034

YNL169C	PSD1	-0.074156	0.789825	0.280898	-0.263216
YBR162C	TOS1	0.043714	0.347432	0.2358	-0.263403
YDR080W	VPS41	0.049805	0.345615	0.099968	-0.265358
YIL029C	YIL029C	-0.242286	0.351515	-0.068122	-0.265397
YGR142W	BTN2	0.253412	0.044775	-0.466039	-0.267647
YNL311C	YNL311C	-0.024201	0.362443	0.096863	-0.268775
YLR097C	YLR097C	-0.384222	0.18486	0.167212	-0.27051
YEL035C	UTR5	-0.009595	0.405199	0.190784	-0.273732
YDR005C	MAF1	0.024186	0.550647	0.47921	-0.274803
YJL222W	VTH2	-0.051939	0.307487	0.098445	-0.274917
YKL004W	AUR1	-0.075106	0.14245	-0.35563	-0.275164
YDR034CD01	#N/A	0.083747	0.673084	0.271648	-0.276201
YIL037C	PRM2	-0.156043	0.080065	0.710041	-0.277608
YBR012W-B	YBR012W-B	0.083441	0.275147	0.331799	-0.277757
YHR215W	PHO12	-0.498884	-0.318693	-0.241414	-0.280131
YGR279C	SCW4	-0.043028	0.386298	0.264839	-0.280258
YER128W	YER128W	-0.341484	0.672298	0.141151	-0.281772
YJL078C	PRY3	-0.210817	0.785318	0.302566	-0.283736
YOL083W	YOL083W	-0.069194	0.332008	0.062755	-0.283737
YDL124W	YDL124W	-0.575265	0.185936	0.516487	-0.283977
YIR028W	DAL4	-0.091494	0.647581	0.522906	-0.284011
YHR186C	YHR186C	-0.596753	0.410164	0.011863	-0.286146
YLL024C	SSA2	0.059893	-0.013924	-0.383579	-0.287017
YBR249C	ARO4	-0.043564	-0.227386	-0.447026	-0.287905
YLR178C	TFS1	-0.372903	0.679849	1.013938	-0.288971
YJL089W	SIP4	0.067765	0.388471	0.159068	-0.292783
YDL244W	THI13	-0.047272	0.515982	0.343001	-0.293967
YNL058C	YNL058C	0.147787	0.648448	0.336928	-0.294952
YGR023W	MTL1	0.031938	0.573722	0.21169	-0.29533
YNL125C	ESBP6	0.276995	0.439883	0.229442	-0.296086
YBR046C	ZTA1	-0.144379	0.482375	0.562569	-0.296404
YML045W-A	YML045W-A	0.082624	0.580884	0.311449	-0.297185
YPL243W	SRP68	-0.417588	0.739453	0.631788	-0.298864
YNL239W	LAP3	0.210379	0.657085	0.382622	-0.299048
YNL293W	MSB3	-0.096283	0.402055	0.1588	-0.300181
YMR130W	YMR130W	-0.46694	-0.355317	-0.295487	-0.302986
YLR258W	GSY2	-0.230747	0.290587	0.396942	-0.302993
YOR202W	HIS3	0.083657	0.483422	0.239399	-0.304291
YLR042C	YLR042C	-0.29137	-0.062076	-0.06588	-0.30514
YMR105C	PGM2	-0.171459	0.141294	0.289993	-0.305648
YAL005C	SSA1	0.096351	0.098236	-0.29625	-0.307057
YLR164W	YLR164W	-0.068666	0.413708	0.33312	-0.307233
YDR421W	YDR421W	-0.041575	0.390117	0.194497	-0.308134
YLR152C	YLR152C	-0.243353	0.336193	0.080477	-0.30875
YCL055W	KAR4	-0.086954	0.172152	0.03062	-0.311346
YMR103C	YMR103C	-0.06899	0.595722	0.405981	-0.311395
YDL167C	NRP1	0.126337	0.201288	-0.16141	-0.311733
YGR071C	YGR071C	-0.048356	-0.075956	-0.018561	-0.312614
YER061C	CEM1	-0.116188	0.420129	0.362109	-0.313681
YGR240C	PFK1	-0.190022	0.100428	-0.027651	-0.316188
YLL026W	HSP104	0.024947	0.22527	0.068608	-0.316404
YDR070C	YDR070C	-0.140641	0.301042	-0.072849	-0.317402

YKR042W	UTH1	-0.082779	0.482167	0.202749	-0.317931
YOR011W	AUS1	0.037341	0.247451	0.015136	-0.317988
YNL334C	SNO2	-0.227884	0.185502	0.196725	-0.318192
YDR403W	DIT1	-0.071736	0.119551	0.100275	-0.318735
YPL092W	SSU1	-0.039634	0.305479	0.046372	-0.319207
YMR170C	ALD2	-0.073632	0.566681	0.427302	-0.322337
YHR041C	SRB2	-0.288401	0.003319	0.039454	-0.322464
YOR153W	PDR5	-0.142479	0.146548	0.026362	-0.322676
YAL037W	YAL037W	-0.080948	0.208156	0.09301	-0.324096
YLR001C	YLR001C	0.012776	0.257524	0.241533	-0.326049
YER091C	MET6	-0.244049	0.08048	0.165706	-0.326589
YDR406W	PDR15	-0.292158	-0.049953	-0.074819	-0.327252
YLR355C	ILV5	-0.157798	-0.271827	-0.356595	-0.327573
YOL153C	YOL153C	-0.002207	0.357337	0.332761	-0.328901
YHR084W	STE12	-0.115272	0.006766	-0.064205	-0.329808
YBR287W	YBR287W	-0.152758	0.468055	0.51843	-0.330537
YKL163W	PIR3	0.029939	0.615176	0.445292	-0.330708
YOL064C	MET22	-0.211772	0.16805	0.002512	-0.331633
YHR162W	YHR162W	0.008651	0.416008	0.230073	-0.331778
YDR037W	KRS1	0.187571	0.019958	-0.34292	-0.332112
YNL296W	YNL296W	0.047669	0.274371	0.004913	-0.332124
YER103W	SSA4	-0.07623	0.199543	-0.014161	-0.332647
YPL264C	YPL264C	-0.053107	0.269528	0.256094	-0.332905
YIR034C	LYS1	0.144586	0.697962	0.402139	-0.333875
YKL051W	YKL051W	-0.005803	0.220934	0.139885	-0.335336
YEL057C	YEL057C	0.030477	0.169347	0.104031	-0.335578
YDR354W	TRP4	0.085276	0.37593	0.035347	-0.335737
YER187W	YER187W	-0.237968	0.004786	0.026184	-0.335767
YNL114C	YNL114C	-0.194355	0.055255	0.104299	-0.336028
YGL146C	YGL146C	-0.156883	0.234847	0.117102	-0.337479
YDR474C	YDR474C	-0.062401	0.275409	0.176646	-0.338405
YCR089W	FIG2	-0.094202	0.295183	0.074137	-0.339749
YGL028C	SCW11	-0.28487	-0.017087	0.063928	-0.341019
YHR190W	ERG9	-0.193453	0.539324	0.365968	-0.341706
YIL166C	YIL166C	-0.189057	0.373307	0.164554	-0.342002
YIL111W	COX5B	-0.323242	0.303922	0.584559	-0.342079
YKL162C-A	YKL162C-A	-0.154062	0.435802	0.179439	-0.342965
YKR097W	PCK1	-0.025771	0.342241	0.179135	-0.347761
YLR252W	YLR252W	-0.292706	0.57042	0.723202	-0.348519
YLR142W	PUT1	0.139068	0.766015	0.641795	-0.348712
YIR017C	MET28	-0.076154	0.34949	0.181437	-0.349311
YFL026W	STE2	-0.198013	0.280251	0.297982	-0.349603
YER062C	HOR2	-0.189016	-0.135466	-0.231921	-0.351101
YFL060C	SNO3	-0.247745	0.194937	0.051692	-0.351807
YBR115C	LYS2	0.14623	0.533484	0.01268	-0.353166
YLR422W	YLR422W	-0.046893	0.036739	0.070206	-0.35365
YDR085C	AFR1	0.014194	0.5659	0.339826	-0.354698
YOL134C	YOL134C	-0.047621	0.303521	0.080717	-0.355253
YBR256C	RIB5	0.105681	0.50822	0.215852	-0.359916
YHR021W-A	ECM12	0.082909	0.052483	-0.061016	-0.361544
YDL246C	YDL246C	-0.356074	0.169535	0.191564	-0.362333
YCR099C	YCR099C	0.104223	0.428006	0.14003	-0.36415

YNL015W	PBI2	-0.228142	0.432731	0.457772	-0.365132
YER124C	YER124C	-0.261197	-0.005672	0.137381	-0.365565
YMR271C	URA10	-0.142688	0.351465	0.187435	-0.366981
YHR005C	GPA1	-0.169324	0.041728	0.026451	-0.367169
YGR086C	YGR086C	-0.067957	0.27544	0.180912	-0.368294
YIL015C-A	YIL015C-A	-0.17153	0.17823	-0.049144	-0.369223
YKL185W	ASH1	-0.202577	-0.094848	0.174841	-0.369613
YGL259W	YPS5	-0.024561	0.488106	0.32204	-0.370448
YBR104W	YMC2	0.000783	0.133635	-0.017634	-0.370511
YPL280W	YPL280W	-0.033027	0.225809	-0.086866	-0.371297
YJL108C	PRM10	0.014486	0.187684	-0.03469	-0.37149
YML116W	ATR1	-0.041568	0.394431	0.149718	-0.371596
YNR069C	YNR069C	0.154367	0.537992	0.151706	-0.371613
YER090W	TRP2	0.180452	0.276823	-0.10438	-0.371968
YDL066W	IDP1	0.002413	0.870355	0.708827	-0.371977
YBR169C	SSE2	-0.123941	0.557275	0.254705	-0.372113
YGL248W	PDE1	-0.179289	0.585086	0.448765	-0.373183
YIL082W	YIL082W	-0.115673	0.042313	-0.060842	-0.375876
YKL211C	TRP3	0.368065	0.514437	0.160595	-0.377116
YOR382W	YOR382W	-0.306987	0.12416	0.309617	-0.377768
YHR059W	YHR059W	-0.307265	0.344675	-0.021445	-0.380198
YGL202W	ARO8	0.15963	0.52498	0.145151	-0.380362
YGL117W	YGL117W	0.014116	0.356986	0.312346	-0.384817
YIL082W-A	YIL082W-A	-0.105787	0.249007	0.011037	-0.390803
YJL157C	FAR1	-0.360245	0.001716	-0.177794	-0.394371
YDR127W	ARO1	0.062123	0.54332	0.114887	-0.394564
YKL103C	LAP4	-0.172764	1.013898	0.93521	-0.397741
YIL009C-A	EST3	-0.23825	0.381858	0.250242	-0.398817
YER069W	ARG5	-0.011835	0.541998	0.323904	-0.399564
YIL080W	YIL080W	-0.12394	0.236733	0.100068	-0.40194
YPR005C	HAL1	-0.188569	0.342575	0.0785	-0.402921
YGL008C	PMA1	-0.163021	0.087463	0.073131	-0.403357
YGR109W-B	YGR109W-B	-0.12085	0.209758	0.055341	-0.40518
YKL184W	SPE1	-0.046916	-0.073397	-0.038922	-0.408222
YDR195W	REF2	-0.200272	0.279089	-0.002878	-0.40965
YER055C	HIS1	0.060489	0.388926	-0.042669	-0.40977
YPL058C	PDR12	-0.10724	0.171046	-0.035309	-0.410013
YIR042C	YIR042C	-0.211233	0.271643	0.246561	-0.413973
YML047C	PRM6	-0.083426	0.137588	0.174358	-0.416169
YBR183W	YPC1	-0.360249	0.288321	0.326935	-0.41755
YDR022C	CIS1	-0.225598	0.221287	0.17624	-0.419312
YIL015W	BAR1	-0.258876	0.135815	0.146635	-0.419639
YNR006W	VPS27	-0.017534	0.114449	0.060121	-0.419809
YGL249W	ZIP2	-0.48171	0.297508	0.121583	-0.420382
YML128C	YML128C	-0.091135	0.248486	0.236334	-0.421026
YLR286C	CTS1	-0.291034	0.216107	0.200342	-0.423777
YDR380W	YDR380W	-0.387843	0.978495	1.105585	-0.424258
YLR040C	YLR040C	-0.210411	0.140804	0.007752	-0.42998
YDR158W	HOM2	0.070999	0.300643	-0.080463	-0.43141
YHR071W	PCL5	-0.001653	0.419492	0.071148	-0.434594
YGR045C	YGR045C	-0.50939	0.482198	0.35824	-0.435918
YGR146C	YGR146C	-0.223005	0.455955	-0.040668	-0.438244

YML100W	TSL1	-0.382924	0.346232	0.534967	-0.439893
YGR248W	SOL4	-0.231611	0.365474	0.341212	-0.441817
YPL036W	PMA2	-0.221191	0.221415	-0.003495	-0.450778
YLR327C	YLR327C	-0.289002	0.183661	0.04854	-0.452206
YOR344C	TYE7	-0.296214	-0.29066	-0.429219	-0.460682
YDR242W	AMD2	-0.216534	0.582242	0.384142	-0.461206
YOR130C	ORT1	-0.052211	0.687063	0.262181	-0.461525
YGR044C	RME1	-0.198599	0.388402	0.356749	-0.463962
YHL044W	YHL044W	-0.33031	0.208663	0.163649	-0.465728
YHR143W	YHR143W	-0.228154	0.348587	0.1428	-0.468622
YOR173W	YOR173W	-0.190927	0.307119	0.142467	-0.468968
YER096W	SHC1	0.058543	0.362562	-0.069738	-0.472529
YHR136C	SPL2	-0.708303	-0.530314	-0.164793	-0.473752
YMR062C	ECM40	0.05744	0.583549	0.263236	-0.473956
YDL024C	DIA3	-0.334823	-0.084242	-0.057837	-0.474197
YDR276C	PMP3	-0.22416	0.606718	0.341807	-0.475874
YLR058C	SHM2	-0.278496	0.679907	0.371355	-0.476756
YHR018C	ARG4	0.044947	0.766549	0.499888	-0.477498
YLR452C	SST2	-0.263218	0.190769	0.095315	-0.477844
YLR231C	YLR231C	-0.340625	0.047456	0.027951	-0.479202
YBR019C	GAL10	-0.26304	0.297299	0.118725	-0.479889
YKL198C	PTK1	-0.322506	0.461861	-0.020557	-0.484809
YDR035W	ARO3	0.015042	0.400705	0.008138	-0.485976
YHR137W	ARO9	-0.171875	0.849595	0.823657	-0.486721
YER150W	SPI1	-0.284042	0.157473	0.161233	-0.488071
YBR047W	YBR047W	-0.232982	0.590797	0.421703	-0.490378
YOR161C	YOR161C	-0.325614	0.042306	0.091225	-0.491724
YHR055C	CUP1-2	-0.187186	0.078795	-0.360055	-0.492158
YOL052C-A	DDR2	-0.122586	0.703923	0.531207	-0.496948
YPR149W	NCE102	-0.129731	0.225901	0.050066	-0.498487
YNL145W	MFA2	-0.511145	0.546998	0.428067	-0.509493
YDL021W	GPM2	-0.383405	0.285419	0.319147	-0.515086
YDR461W	MFA1	-0.448953	0.543978	0.369231	-0.519327
YHR053C	CUP1-1	-0.166623	0.06233	-0.343291	-0.51944
YDR533C	YDR533C	-0.239769	0.508582	0.405639	-0.524845
YNR068C	YNR068C	0.048953	0.66567	0.197678	-0.531243
YHR087W	YHR087W	-0.291229	0.280079	0.086917	-0.543689
YDR539W	YDR539W	-0.298457	0.16781	-0.016633	-0.545101
YPL017C	YPL017C	-0.078845	0.486604	0.231652	-0.554159
YIL116W	HIS5	-0.027416	0.727489	0.225026	-0.559924
YMR173W	DDR48	-0.451869	0.50679	0.601642	-0.565994
YPL088W	YPL088W	-0.082344	0.582238	0.10663	-0.566916
YDR034W-B	YDR034W-B	-0.075265	0.481306	0.215965	-0.571688
YER073W	ALD5	-0.06945	0.080313	-0.313855	-0.575212
YMR095C	SNO1	-0.210673	0.595927	-0.003512	-0.578372
YJL172W	CPS1	-0.176631	1.15873	0.821546	-0.581988
YJR109C	CPA2	0.230992	1.06566	0.435157	-0.600245
YER175C	YER175C	-0.076483	0.817369	0.417705	-0.604561
YOR302W	YOR302W	-0.037873	0.905205	0.141217	-0.609478
YPL250C	ICY2	-0.138315	0.642618	0.284234	-0.615706
YJL170C	ASG7	-0.462173	0.42178	0.122168	-0.61595
YER052C	HOM3	0.078179	0.512225	-0.069507	-0.627622

YGL121C	YGL121C	-0.278984	1.010944	0.745394	-0.630517
YKL218C	SRY1	-0.126132	0.728258	0.408319	-0.639946
YML123C	PHO84	-0.46475	-0.099003	-0.196904	-0.647639
YCL030C	HIS4	0.29915	1.021913	0.154499	-0.649936
YIL164C	NIT1	-0.105998	0.805832	0.179743	-0.653088
YPR160W	GPH1	-0.161789	0.570299	0.461824	-0.654157
YDR453C	YDR453C	-0.13258	0.728644	0.439639	-0.664586
YIL165C	YIL165C	-0.191721	0.6157	0.055208	-0.697728
YKL096W	CWP1	-0.241469	0.287099	-0.128879	-0.71544
YOR303W	CPA1	0.096226	0.84978	0.174042	-0.721809
YCL027W	FUS1	-0.466599	0.313765	0.119912	-0.726476
YHR029C	YHR029C	-0.156708	0.744605	0.346573	-0.744948
YBR072W	HSP26	-0.414335	0.474147	0.20479	-0.757502
YBR285W	YBR285W	-0.136539	0.61513	0.375846	-0.757813
YDR033W	MRH1	-0.778339	0.199817	0.284524	-0.760212
YGL032C	AGA2	-0.542064	0.433769	0.222921	-0.769891
YJR025C	BNA1	-0.325773	0.311586	0.192118	-0.794256
YNR044W	AGA1	-0.410736	0.543859	0.252537	-0.823156
YHR022C	YHR022C	-0.201995	0.605543	0.087981	-0.856121
YMR096W	SNZ1	0.006831	1.048419	0.074325	-0.943327
YNL160W	YGP1	-0.695198	0.304312	0.16843	-0.95492
YBR054W	YRO2	-0.37618	0.425113	-0.011021	-0.99495
YFL014W	HSP12	-0.463788	0.787702	0.353922	-1.064175
