# Supplementary material for: Genetic Interactions of MAF1 Identify a Role for Med20 in Transcriptional Repression of Ribosomal Protein Genes
Source: PLoS Genet. 2008 Jul 4;4(7):e1000112. doi: 10.1371/journal.pgen.1000112 (PMC2435279; doi:10.1371/journal.pgen.1000112)
Supplement: Table S3 — Yeast GO bioprocess terms represented in merged med20Δ versus wild-type datasets. (0.04 MB PDF) [file pgen.1000112.s007.pdf]

Table S3

Yeast GO Bioprocess Terms Represented in Merged *med20Δ* versus Wild-type Datasets.

Genes underexpressed in *med20Δ* versus wild-type strains

GOID	GO term	Frequency	Gene(s)
6950	response to stress	16 out of 106 genes, 15.1%	PRX1,HSP26,PHO5,HSP30,GPD1,HSP12,STF2,YHB1,WSC4,KOG1,TSL1,DDR48,GAD1,HOR7,YGP1,ATX1
6810	transport	15 out of 106 genes, 14.2%	GLK1,HXT7,HXT6,PIC2,HXK1,ZRT1,STF2,TPO2,WSC4,PTK1,PHO84,ATX1,TPO4,FIT2,SRP68
5975	carbohydrate metabolic process	11 out of 106 genes, 10.4%	TKL2,GLK1,SOR2,ICL1,HXK1,TSL1,PGM2,MSS11,TYE7,CIT3,GPH1
16070	RNA metabolic process	8 out of 106 genes, 7.5%	RSC6,EMI2,PNC1,BCD1,YHR087W,MSS11,TYE7
746	conjugation	7 out of 106 genes, 6.6%	FUS1,MFA1,AGA2,FAR1,ASG7,MFA2,AGA1
6996	organelle organization and biogenesis	7 out of 106 genes, 6.6%	GCV3,RSC6,PNC1,ZIP2,FYV4,YMR134W
6091	generation of precursor metabolites and energy	6 out of 106 genes, 5.7%	BDH1,STF2,COX5B,TSL1,CIT3,GPH1
6350	transcription	6 out of 106 genes, 5.7%	RSC6,EMI2,PNC1,MSS11,TYE7
6519	amino acid and derivative metabolic process	6 out of 106 genes, 5.7%	GCV3,CHA1,GCV1,ARO10,SDL1,GAD1
9653	anatomical structure morphogenesis	5 out of 106 genes, 4.7%	PHO5,FUS1,DIA3,KOG1,MSS11
6766	vitamin metabolic process	5 out of 106 genes, 4.7%	TKL2,GPD1,PNC1,BNA1,BNA5
7165	signal transduction	4 out of 106 genes, 3.8%	MFA1,KOG1,FAR1,MFA2
6259	DNA metabolic process	4 out of 106 genes, 3.8%	RSC6,PNC1,MSC1,DDR48
19725	cellular homeostasis	4 out of 106 genes, 3.8%	PRX1,GPD1,YMR134W,ATX1
7049	cell cycle	4 out of 106 genes, 3.8%	ZIP2,FAR1,MSC1,TYE7
7047	cell wall organization and biogenesis	4 out of 106 genes, 3.8%	DSE1,DSE2,CWP1,ECM3
910	cytokinesis	2 out of 106 genes, 1.9%	SCW11,DSE2
7126	meiosis	2 out of 106 genes, 1.9%	ZIP2,MSC1
7124	pseudohyphal growth	2 out of 106 genes, 1.9%	DIA3,MSS11
6118	electron transport	1 out of 106 genes, 0.9%	COX5B
45333	cellular respiration	1 out of 106 genes, 0.9%	CIT3
6464	protein modification process	1 out of 106 genes, 0.9%	HRT3
30435	sporulation	1 out of 106 genes, 0.9%	EMI2
6629	lipid metabolic process	1 out of 106 genes, 0.9%	YPC1
30163	protein catabolic process	1 out of 106 genes, 0.9%	HRT3
42254	ribosome biogenesis and assembly	0 out of 106 genes, 0%	none
16044	membrane organization and biogenesis	0 out of 106 genes, 0%	none
6997	nuclear organization and biogenesis	0 out of 106 genes, 0%	none
7114	cell budding	0 out of 106 genes, 0%	none
7010	cytoskeleton organization and biogenesis	0 out of 106 genes, 0%	none
16192	vesicle-mediated transport	0 out of 106 genes, 0%	none
6412	translation	0 out of 106 genes, 0%	none

Table S3 continued
Yeast GO Bioprocess Terms Represented in Merged *med20Δ* versus Wild-type Datasets.

Genes overexpressed in *med20Δ* versus wild-type strains

GOID	GO term	Frequency	Gene(s)
6519	amino acid and derivative metabolic process	13 out of 56 genes, 23.2%	CYS3,ILV6,SER3,ASN2,THR1,MET28,TRP3,ASP3-1,ASP3-2,ASP3-3,ASP3-4,ARG1,GDH1
6412	translation	8 out of 56 genes, 14.3%	MNP1,ZUO1,SSZ1,MRPL8,RSM7,MRPL31,RPP0,MRP51
6810	transport	8 out of 56 genes, 14.3%	SIT1,SSC1,VPS1,GAP1,COX17,HSP60,FIT2,FIT3
6950	response to stress	7 out of 56 genes, 12.5%	ASP3-1,ASP3-2,ASP3-3,ASP3-4,SCJ1,NCE103,HSP82
6996	organelle organization and biogenesis	6 out of 56 genes, 10.7%	MGR1,NOP7,MRPL8,VPS1,XDJ1,RPP0
7047	cell wall organization and biogenesis	3 out of 56 genes, 5.4%	TIP1,CDC3,SPO77
16070	RNA metabolic process	2 out of 56 genes, 3.6%	NOP7,MET28
7049	cell cycle	2 out of 56 genes, 3.6%	NOP7,PCL5
42254	ribosome biogenesis and assembly	2 out of 56 genes, 3.6%	NOP7,RPP0
910	cytokinesis	1 out of 56 genes, 1.8%	CDC3
7010	cytoskeleton organization and biogenesis	1 out of 56 genes, 1.8%	VPS1
746	conjugation	1 out of 56 genes, 1.8%	PRM1
6091	generation of precursor metabolites and energy	1 out of 56 genes, 1.8%	MNP1
6350	transcription	1 out of 56 genes, 1.8%	MET28
7114	cell budding	1 out of 56 genes, 1.8%	CDC3
45333	cellular respiration	1 out of 56 genes, 1.8%	MNP1
7165	signal transduction	1 out of 56 genes, 1.8%	SCJ1
30163	protein catabolic process	1 out of 56 genes, 1.8%	SCJ1
9653	anatomical structure morphogenesis	1 out of 56 genes, 1.8%	CDC3
16044	membrane organization and biogenesis	1 out of 56 genes, 1.8%	PRM1
6464	protein modification process	1 out of 56 genes, 1.8%	CYC3
30435	sporulation	1 out of 56 genes, 1.8%	SPO77
19725	cellular homeostasis	1 out of 56 genes, 1.8%	SIT1
6997	nuclear organization and biogenesis	0 out of 56 genes, 0%	none
6766	vitamin metabolic process	0 out of 56 genes, 0%	none
6259	DNA metabolic process	0 out of 56 genes, 0%	none
5975	carbohydrate metabolic process	0 out of 56 genes, 0%	none
6118	electron transport	0 out of 56 genes, 0%	none
16192	vesicle-mediated transport	0 out of 56 genes, 0%	none
6629	lipid metabolic process	0 out of 56 genes, 0%	none
7124	pseudohyphal growth	0 out of 56 genes, 0%	none
7126	meiosis	0 out of 56 genes, 0%	none
