# Supplementary material for: Genetic Interactions of MAF1 Identify a Role for Med20 in Transcriptional Repression of Ribosomal Protein Genes
Source: PLoS Genet. 2008 Jul 4;4(7):e1000112. doi: 10.1371/journal.pgen.1000112 (PMC2435279; doi:10.1371/journal.pgen.1000112)
Supplement: Table S4 — Expression ratios (log base 10) comparing med20Δ versus wild-type strains under different repressing conditions. (0.41 MB PDF) [file pgen.1000112.s008.pdf]

Table S4

Expression ratios (log base 10) comparing med20Δ versus wild-type strains under different repressing conditions

The data have been filtered to select genes whose expression increased or decreased two fold or more in any of the six conditions

UNIQID	Gene Name	med20/WT + CPZ	med20/WT + Tun	med20/WT + Rap	med20/WT post-diauxic shift	med20/WT + 39°C	med20/WT + H2O2
YLR158C	ASP3-3	0.410483	0.368541	NaN	0.344405	0.513131	0.209393
YLR160C	ASP3-4	0.399223	0.373836	NaN	0.322089	0.501698	0.224975
YKL010C	UFD4	-0.032438	-0.01506	NaN	-0.381176	-0.03946	0.03094
YPL081W	RPS9A	0.391589	0.443295	0.792405	0.084829	-0.192565	-0.220462
YLR185W	RPL37A	0.233866	0.258443	0.705401	0.323472	-0.001406	-0.081463
YHR061C	GIC1	0.051333	-0.052266	0.688293	0.14648	0.192479	0.125596
YDR500C	RPL37B	0.224098	0.265027	0.688107	0.352047	0.001827	-0.027847
YLL009C	COX17	0.717663	0.677414	0.682061	0.778913	0.334059	0.705616
YBL071C	YBL071C	0.070486	-0.079693	0.668368	-0.127289	-0.090836	-0.197597
YBR189W	RPS9B	0.277546	0.437632	0.661152	0.192406	-0.181065	-0.206546
YOL040C	RPS15	0.328483	0.469024	0.637436	0.293758	0.159913	-0.075712
YLR406C	RPL31B	0.230141	0.216856	0.635714	0.226366	-0.107939	-0.079661
YIL018W	RPL2B	0.201167	0.330847	0.622047	0.151181	0.029201	-0.146089
YFR031C-A	RPL2A	0.208509	0.344808	0.582971	0.123202	-0.138752	-0.152433
YGL189C	RPS26A	0.172511	0.248373	0.577591	0.204386	0.19805	-0.063803
YGR214W	RPS0A	0.190469	0.421905	0.576644	0.036457	0.002275	-0.145539
YGR034W	RPL26B	0.269672	0.434331	0.570964	0.383549	-0.110274	-0.097611
YOL039W	RPP2A	0.154861	0.372864	0.56857	0.243187	0.164951	-0.089765
YKR057W	RPS21A	0.303123	0.444206	0.563017	0.340418	0.201607	-0.18405
YFL034C-A	RPL22B	0.261085	0.331747	0.557962	0.246217	-0.018987	-0.23719
YDL082W	RPL13A	0.26553	0.359156	0.557959	0.140809	0.033094	-0.040861
YBR040W	FIG1	NaN	-0.523687	0.555703	-0.678834	-0.39772	-0.782338
YLR367W	RPS22B	0.204148	0.452012	0.548732	0.324521	0.089823	-0.176378
YBR048W	RPS11B	0.306095	0.322184	0.546169	0.173002	0.061122	-0.011817
YPL249C-A	RPL36B	0.244937	0.194899	0.545808	0.272879	0.359539	0.018772
YJR094W-A	RPL43B	0.160792	0.251163	0.544692	0.423436	0.038495	-0.023261
YDR471W	RPL27B	0.384809	0.38117	0.540626	0.181567	-0.09952	-0.065677
YML024W	RPS17A	0.161466	0.490445	0.538612	0.318448	-0.05763	-0.1055
YER102W	RPS8B	0.312408	0.424209	0.536283	0.084388	0.101714	-0.000223
YLL045C	RPL8B	0.173555	0.400705	0.533813	0.131507	0.084737	-0.158635
YPL079W	RPL21B	0.535535	0.443682	0.532388	0.051378	-0.146176	-0.141607
YGL147C	RPL9A	0.212502	0.490069	0.521344	0.211013	-0.162322	-0.181934
YOR293W	RPS10A	0.259574	0.470791	0.520309	0.262671	-0.086061	-0.101412
YHR021C	RPS27B	0.056407	0.487769	0.517029	0.252965	-0.364286	-0.155521
YOR167C	RPS28A	0.126915	0.142342	0.514154	0.350328	0.099159	-0.140972
YDR447C	RPS17B	0.211208	0.510304	0.513686	0.166362	-0.032881	-0.155261
YFR032C-A	RPL29	0.187803	0.045064	0.512835	0.081605	-0.190759	0.033434
YER074W	RPS24A	0.166174	0.32872	0.512531	0.276126	0.065764	-0.008182
YER058W	PET117	0.102417	0.221411	0.509352	0.353832	0.063606	0.255433
YJL190C	RPS22A	0.267	0.411998	0.507972	0.325178	0.062398	-0.266188
YBL072C	RPS8A	0.367569	0.446078	0.505028	0.114309	0.136098	-0.063667
YLR264W	RPS28B	0.172757	0.206097	0.504382	0.34264	0.059013	-0.153033
YBR191W	RPL21A	0.300784	0.573047	0.504293	0.156741	-0.23133	-0.149896

YBL087C	RPL23A	0.239184	0.379934	0.50283	0.300545	-0.008098	-0.068932
YBR071W	YBR071W	0.180768	0.175802	0.495155	0.04537	0.276773	0.369628
YPL143W	RPL33A	0.174872	0.400272	0.493553	0.307383	-0.156373	-0.034114
YPL198W	RPL7B	0.380328	0.500574	0.491217	0.195756	0.135597	-0.192329
YER131W	RPS26B	0.185748	0.232817	0.491196	0.178412	0.240295	-0.010472
YGL068W	YGL068W	0.513192	0.421909	0.483462	0.393093	0.161526	0.225001
YDR450W	RPS18A	0.236168	0.402508	0.482657	0.093271	0.156065	-0.153286
YGL076C	RPL7A	0.327603	0.459276	0.482097	0.163811	0.159369	-0.271195
YDL213C	YDL213C	0.061665	0.146346	0.48058	0.326591	-0.087864	-0.008179
YGL103W	RPL28	0.059891	0.324025	0.480058	0.195046	0.268941	-0.05271
YDR418W	RPL12B	0.212848	0.526549	0.479573	0.210956	-0.179379	-0.181766
YER056C-A	RPL34A	0.254927	0.253246	0.475857	0.225992	0.015209	0.029243
YMR142C	RPL13B	0.157977	0.203284	0.475665	0.13347	0.141145	0.00671
YOL127W	RPL25	0.266965	0.478228	0.47215	0.19738	0.048352	-0.042369
YPL131W	RPL5	0.184783	0.258642	0.471607	0.134204	-0.031988	-0.077726
YIL070C	MAM33	0.480009	0.604843	0.469288	0.278502	0.100105	0.487417
YOR234C	RPL33B	0.081091	0.35239	0.467382	0.289399	-0.106026	-0.116194
YOR020C	HSP10	0.212971	0.343527	0.463932	0.369255	0.313899	0.405313
YHR010W	RPL27A	0.296147	0.419546	0.453166	0.194122	-0.097971	-0.053607
YDR025W	RPS11A	0.259931	0.276525	0.451136	0.224849	-0.012444	-0.038392
YJL180C	ATP12	0.214403	0.29094	0.45012	0.10578	0.175571	0.357295
YDL165W	CDC36	0.051376	0.053264	0.448653	0.128161	0.212915	0.003238
YBL074C	AAR2	-0.06691	0.109915	0.448	-0.14222	0.017281	0.010057
YGR109C	CLB6	0.137462	0.057809	0.447643	0.438567	-0.09864	-0.168912
YLR344W	RPL26A	0.22195	0.347486	0.447175	0.202809	-0.116745	-0.065663
YNL069C	RPL16B	0.29926	0.380546	0.440531	0.11974	-0.19964	-0.063461
YNR037C	RSM19	0.404501	0.261229	0.437527	0.43801	0.23976	0.345838
YLR333C	RPS25B	0.099656	0.073813	0.435642	0.111632	0.176978	0.006
YLR048W	RPS0B	0.255568	0.327078	0.429804	0.066698	-0.031778	-0.040463
YDR064W	RPS13	0.129506	0.351113	0.427738	0.187521	0.077466	-0.216283
YKR085C	MRPL20	0.500146	0.495788	0.42716	0.321056	0.209993	0.374012
YHR141C	RPL42B	0.212297	0.329195	0.426889	0.330433	-0.224438	-0.098538
YML009C	MRPL39	0.404876	0.22065	0.425152	0.675053	0.106857	0.347442
YAL003W	EFB1	0.09017	0.224239	0.42319	-0.029073	0.159882	-0.321055
YMR143W	RPS16A	0.109346	0.259084	0.422739	0.109635	-0.080102	-0.0769
YER117W	RPL23B	0.247238	0.326839	0.420713	0.218249	0.047912	-0.054425
YGR140W	CBF2	0.08838	0.037997	0.419817	-0.063225	-0.072862	-0.257711
YPL072W	UBP16	0.289832	0.249006	0.419687	0.199204	0.291255	0.288083
YJL191W	RPS14B	0.198341	0.332396	0.417614	0.172399	0.178962	0.004285
YJR048W	CYC1	0.271246	0.295422	0.416884	0.233364	-0.109968	0.596666
YBL027W	RPL19B	0.255568	0.420594	0.416712	0.056179	-0.105754	-0.001077
YNL162W	RPL42A	0.145356	0.238015	0.416163	0.250773	-0.196402	-0.065054
YML063W	RPS1B	0.166952	0.283266	0.415906	0.064177	-0.096839	-0.058068
YNL067W	RPL9B	0.358063	0.450119	0.411194	0.170452	-0.236213	-0.381718
YDR296W	MHR1	0.535173	0.303708	0.410293	0.273865	0.287264	0.29954
YJR083C	YJR083C	0.008974	0.069792	0.409907	0.410569	0.092236	0.053956
YDR543C	YDR543C	-0.034939	-0.134705	0.408006	-0.083235	0.089419	0.081106
YOR075W	UFE1	0.212339	0.187108	0.407202	0.033812	0.284013	-0.068939
YOR096W	RPS7A	0.298155	0.507796	0.405812	0.104073	-0.206699	-0.244441
YOL071W	YOL071W	0.219093	0.306797	0.404289	0.200416	0.136295	0.548265
YGR215W	RSM27	0.265759	0.208868	0.400247	0.106712	0.044101	-0.06389
YJL189W	RPL39	0.199516	0.111072	0.398495	0.259468	0.012694	-0.058134

YJR145C	RPS4A	0.240342	0.407465	0.39833	0.27418	0.048763	-0.04733
YDR382W	RPP2B	0.199432	0.252783	0.397804	0.343731	0.159336	-0.069618
YIL133C	RPL16A	0.238008	0.368791	0.391845	0.093528	-0.197072	-0.050254
YOL121C	RPS19A	0.24357	0.387693	0.391486	0.24528	0.028691	-0.106277
YGL030W	RPL30	0.11295	0.264653	0.389675	0.108245	-0.060755	-0.156047
YOR369C	RPS12	0.216122	0.5069	0.388255	0.560128	0.270258	-0.067113
YJR123W	RPS5	-0.017958	0.093312	0.38763	-0.003945	-0.249767	-0.140335
YJL177W	RPL17B	0.079248	0.211726	0.383436	0.170264	-0.152446	-0.034602
YKR094C	RPL40B	0.170731	0.411936	0.380461	0.182871	0.008113	-0.029797
YLR075W	RPL10	0.386521	0.47704	0.376675	0.085632	-0.105522	0.054335
YLR340W	RPP0	0.382796	0.525997	0.37464	0.413386	0.420054	0.015658
YDR083W	YDR083W	0.204228	-0.022282	0.371954	0.05409	0.144724	-0.011502
YLR314C	CDC3	0.179237	0.065312	0.370649	0.315875	0.113867	-0.019883
YLR150W	STM1	0.271884	0.375579	0.368421	0.039173	0.136281	-0.078347
YHR203C	RPS4B	0.121187	0.2444	0.365001	0.137919	0.010442	-0.070789
YML073C	RPL6A	0.127343	0.290791	0.364937	0.188901	0.063584	-0.060965
YLR287C-A	RPS30A	0.172897	0.154127	0.359564	0.273099	0.028148	-0.060487
YPR133W-A	TOM5	0.325742	0.176152	0.358756	0.239716	0.086425	0.196127
Q0297	Q0297	0.069134	-0.016558	0.357117	-0.011072	0.283824	0.053693
YOL007C	CSI2	0.35039	-0.030284	0.355542	0.583003	0.196119	0.164445
YPL197C	YPL197C	0.099173	0.25846	0.353701	0.05654	0.151782	-0.052507
YHL001W	RPL14B	0.045812	0.245267	0.350893	0.226762	-0.1287	-0.190351
YCR031C	RPS14A	0.196829	0.24208	0.350842	0.046977	0.160153	-0.023114
YNR072W	HXT17	0.069076	-0.027547	0.349399	-0.047152	0.011777	0.148992
YNL284C	MRPL10	0.119626	0.288258	0.348994	0.252259	0.15256	0.382903
YDR041W	RSM10	0.443643	0.353457	0.348736	0.222218	0.175426	0.413236
YBR037C	SCO1	0.179936	0.304239	0.348494	0.365866	0.27489	0.566007
YMR116C	ASC1	0.318623	0.425669	0.348045	0.170401	0.274952	-0.214158
YJR113C	RSM7	0.288413	0.186371	0.347527	0.15674	0.168598	0.283621
YMR016C	SOK2	0.07586	-0.007227	0.346888	0.174131	0.17777	-0.005766
YOR348C	PUT4	0.192041	-0.067192	0.345096	-0.043215	0.307411	0.137595
YMR242C	RPL20A	0.145257	0.535447	0.341661	0.131791	-0.221046	0.048471
YIL148W	RPL40A	0.20774	0.374421	0.341504	0.125768	-0.029669	-0.140047
YBR069C	TAT1	0.288209	0.116129	0.340058	-0.076884	0.250944	-0.098006
YML026C	RPS18B	0.134155	0.352728	0.339834	0.116948	0.06932	-0.060771
YOR150W	MRPL23	0.242439	0.193892	0.339406	0.232888	0.194365	0.304442
YMR229C	RRP5	-0.042135	0.177106	0.338826	0.08573	0.230673	0.296611
YMR214W	SCJ1	-0.101969	0.076234	0.338039	0.118308	0.096041	0.18834
YDL045W-A	MRP10	-0.001202	0.056738	0.337685	0.25453	0.069673	0.321401
YBL092W	RPL32	-0.029819	0.00448	0.336843	-0.123647	-0.115861	0.067695
YLR341W	YLR341W	0.192337	0.022067	0.335291	0.29298	0.082502	-0.034069
YGR118W	RPS23A	0.11082	0.164243	0.334289	0.229517	-0.066063	-0.107902
YPR102C	RPL11A	0.148607	0.315381	0.334261	0.158054	0.140972	0.202473
YDL227C	HO	0.088696	-0.061113	0.33416	0.195769	0.128132	-0.675624
YNL184C	YNL184C	0.157068	0.374727	0.333158	0.410287	0.177908	0.223672
YLR448W	RPL6B	0.090934	0.214041	0.332598	0.139253	0.05244	-0.04769
YOR312C	RPL20B	0.20456	0.467038	0.331968	0.186733	-0.100017	-0.052044
YNL289W	PCL1	0.425246	-0.137053	0.331473	0.528135	0.215559	0.280727
YJR034W	PET191	0.330195	0.166166	0.331199	0.236554	0.060603	0.062545
YJR118C	ILM1	0.035213	0.049485	0.330807	0.077495	0.043701	0.018096
YCR046C	IMG1	0.401746	0.199297	0.328754	0.175761	0.153598	0.121789
YNL185C	MRPL19	0.205431	0.215971	0.328244	0.179357	0.204269	0.108935

YDR042C	YDR042C	0.496154	0.249985	0.326342	0.114216	0.398561	0.562948
YOL026C	YOL026C	0.166693	0.103886	0.324703	0.303313	0.153587	0.066576
YKL156W	RPS27A	0.117984	0.320751	0.323653	0.25326	-0.280589	-0.256538
YKR044W	YKR044W	0.175426	0.192119	0.323244	0.254317	0.168475	0.240207
YCL044C	YCL044C	-0.040273	-0.055543	0.322095	0.029352	0.10043	0.306508
YMR011W	HXT2	0.030517	-0.022464	0.321224	0.131697	0.070184	0.119375
YKL147C	YKL147C	0.158052	0.0978	0.320896	0.079906	0.155803	-0.206884
YGL135W	RPL1B	0.301901	0.393251	0.320189	0.182194	0.045516	-0.128701
YLR441C	RPS1A	0.173336	0.359969	0.318908	0.06459	-0.236839	-0.004997
YJL115W	ASF1	0.015059	-0.000959	0.315974	-0.044331	0.209618	0.075681
YDR115W	YDR115W	0.130325	0.111418	0.314334	0.124348	0.185969	0.195207
YDL215C	GDH2	0.122284	-0.008595	0.313371	0.465328	0.080784	-0.026976
YGL123W	RPS2	0.092399	0.368877	0.313174	0.35475	0.018118	-0.186379
YKL138C	MRPL31	0.294476	0.318553	0.31147	0.328066	0.198454	0.427897
YLR294C	YLR294C	0.107047	0.149827	0.310647	0.26252	0.082179	0.36779
YDR493W	YDR493W	0.459253	0.283033	0.310084	0.367389	0.13229	0.434485
YJR144W	MGM101	0.092462	0.191207	0.310004	0.143725	0.049254	0.10816
YGR150C	YGR150C	0.223992	0.137117	0.309669	0.102453	0.186859	-0.122816
YLR295C	ATP14	0.147845	0.176308	0.309063	0.238839	0.099952	0.324718
YGR027C	RPS25A	0.186695	0.19766	0.307441	0.240977	0.141481	-0.065338
YKL180W	RPL17A	0.067215	0.287514	0.305242	0.092628	-0.217639	-0.069645
YDR430C	YDR430C	0.29667	0.239455	0.304947	0.064695	0.162303	0.139367
YDL192W	ARF1	0.329319	0.233806	0.304866	0.349881	0.092581	0.162507
YNL101W	YNL101W	0.109214	0.12126	0.30457	0.115304	-0.015402	-0.147654
YLR009W	YLR009W	0.124728	0.071859	0.304569	0.100593	-0.231824	-0.245067
YKL137W	YKL137W	0.039643	0.125218	0.304354	0.275911	0.104403	0.214033
YDR454C	GUK1	0.296119	0.199967	0.304121	0.131508	0.229405	-0.130805
YMR024W	MRPL3	0.442763	0.369587	0.302783	0.212172	0.249603	0.294228
YOL038W	PRE6	0.229191	0.083565	0.30109	0.199256	0.052163	0.164706
YDR144C	MKC7	0.230656	0.11873	0.299253	0.323753	0.025107	-0.231783
YHL011C	PRS3	0.146873	0.3067	0.298342	0.156924	0.132846	-0.039801
YPR100W	YPR100W	0.256938	0.292799	0.296829	0.315932	0.190057	0.251134
YML025C	YML025C	0.167596	0.176807	0.294596	0.189681	0.204247	0.334954
YPL173W	MRPL40	0.313416	0.283331	0.29284	0.066522	0.156553	0.417279
YPL118W	MRP51	0.511617	0.304628	0.291206	0.121479	0.18945	0.259812
YNL005C	MRP7	0.298062	0.227133	0.289384	0.263613	0.229681	0.332492
YGR085C	RPL11B	0.111162	0.337953	0.28932	0.165185	-0.195342	-0.039599
YBR088C	POL30	0.090909	0.127594	0.288631	0.05018	0.034059	-0.365174
YOL077W-A	ATP19	0.233981	0.017421	0.287886	0.313882	0.023691	0.132396
YHR064C	PDR13	0.403042	0.234354	0.287656	0.211547	0.345118	0.094347
YNR036C	YNR036C	0.320866	0.27783	0.286269	0.24331	0.166927	0.177898
YGR182C	YGR182C	0.389742	0.176374	0.285807	-0.158967	0.03276	0.243964
YJL063C	MRPL8	0.485529	0.39896	0.285155	0.09335	0.194713	0.30547
YBR084C-A	RPL19A	0.236677	0.319881	0.285104	0.041142	-0.138841	-0.07629
YGR285C	ZUO1	0.505513	0.2619	0.283333	0.216446	0.323504	0.11811
YNR041C	COQ2	0.204727	0.145434	0.282963	0.359102	0.156751	0.086644
YHR038W	FIL1	-0.004494	0.254248	0.277292	0.315051	-0.083468	-0.18843
YHR005C-A	MRS11	0.299608	0.242414	0.275601	0.397369	-0.044507	0.164499
YJR020W	YJR020W	0.397693	0.16426	0.269058	0.27183	0.228874	0.21667
YKL053C-A	YKL053C-A	0.076566	0.382246	0.269008	0.433716	0.077289	0.395257
YMR002W	YMR002W	0.238834	0.308129	0.265373	0.068517	-0.002721	0.221555
YLL061W	MMP1	-0.346114	-0.016027	0.259984	0.316214	-0.123021	0.061101

YOL152W	FRE7	NaN	0.091936	0.258435	1.24975	-0.212749	0.641374
YOR375C	GDH1	0.252814	0.285229	0.256995	0.434453	0.348078	0.113848
YBR146W	MRPS9	0.486441	0.454438	0.254214	0.290644	0.165338	0.361023
YCR034W	FEN1	0.219304	0.112732	0.252584	0.233446	-0.038343	-0.494619
YBL090W	MRP21	0.183693	0.320163	0.250636	0.224019	-0.036317	0.208936
YNL315C	ATP11	0.490424	0.267527	0.24938	0.134268	0.03036	0.273001
YBR208C	DUR1	-0.01096	-0.145133	0.248365	0.311279	-0.047405	0.041237
YGR076C	MRPL25	0.077421	0.089477	0.248232	0.260954	0.134627	0.39955
YNR022C	YNR022C	0.401307	0.176818	0.247392	0.099112	0.073357	0.193146
YML087C	YML087C	-0.064881	0.080045	0.246398	0.217792	0.036823	1.078359
YER050C	RSM18	0.216461	0.143502	0.245452	0.177617	0.147593	0.31711
YPL098C	YPL098C	0.33757	0.249021	0.24521	0.268751	0.166799	0.084653
Q0115	BI3	0.1139	-0.063798	0.244166	-0.307469	-0.026312	-0.421178
YLR203C	MSS51	0.349259	0.313602	0.239903	0.164968	0.154556	0.397537
YLR167W	RPS31	0.263367	0.494868	0.236729	0.31987	0.031595	-0.015322
YDR045C	RPC11	0.086644	0.135517	0.227645	0.316234	-0.005293	-0.114314
YAL025C	MAK16	0.05895	0.093835	0.224956	0.164959	-0.246827	-0.511433
YHR116W	YHR116W	0.332285	0.200305	0.224602	0.130044	0.109677	0.19861
YLR204W	QRI5	0.411921	0.244198	0.220888	0.321344	0.143436	0.192743
YNL070W	TOM7	0.296617	0.387742	0.220066	0.495565	0.07649	0.169593
YGR055W	MUP1	-0.510421	-0.141523	0.219673	-0.093863	-0.044554	0.085825
YOR310C	NOP58	0.23634	0.28184	0.219049	0.388265	-0.118209	-0.402371
YBR185C	MBA1	0.417804	0.414748	0.218987	0.320254	0.138732	0.403437
YOR187W	TUF1	0.314053	0.374939	0.218314	0.369333	0.147663	0.188951
YLR106C	YLR106C	0.333822	0.355021	0.217024	0.025314	0.277299	0.026984
YPR020W	ATP20	0.024175	0.004186	0.216464	0.358395	0.028656	0.015832
YMR260C	TIF11	0.342066	0.256482	0.215526	0.197137	0.1366	0.02503
YDL052C	SLC1	0.064448	0.074501	0.215424	0.376879	-0.008865	0.036882
YML051W	GAL80	0.436356	0.176698	0.214823	0.242929	0.308651	0.23406
YOR004W	YOR004W	0.20218	0.134334	0.212211	0.12232	-0.33727	-0.215168
YER070W	RNR1	0.067419	0.039935	0.210952	0.180483	-0.008206	-0.496385
YPL215W	CBP3	0.13229	0.144883	0.209541	0.064106	0.069705	0.320318
YBR262C	YBR262C	0.016357	0.13655	0.209414	0.376258	0.056701	0.280817
YNL061W	NOP2	0.328784	0.281243	0.207755	0.096166	-0.222823	-0.121747
YNL081C	YNL081C	0.196575	0.321286	0.206333	0.180397	0.01415	0.152527
YGR165W	YGR165W	0.238386	0.263107	0.206199	0.221901	0.110291	0.37364
YNL213C	YNL213C	0.063242	0.081688	0.205735	0.11027	0.299844	0.301698
YNL096C	RPS7B	0.12736	0.427831	0.204671	0.176764	-0.226384	-0.387608
YOR309C	YOR309C	0.26817	0.326974	0.202579	0.319723	-0.248703	-0.408183
YBR181C	RPS6B	0.244441	0.405619	0.201896	0.077926	-0.270929	-0.190248
YIL134W	FLX1	0.303889	0.330163	0.201449	0.341042	0.038525	0.134577
YDR101C	YDR101C	0.387458	0.142786	0.200512	0.056326	-0.247598	-0.262804
YIL073C	SPO22	0.116351	0.152526	0.200056	0.284369	0.166395	0.477592
YPL104W	MSD1	0.301657	0.240926	0.199769	0.281884	0.16349	0.287778
YJR135W-A	TIM8	0.279061	0.217115	0.199165	0.397379	0.047521	-0.012304
YCR087C-A	YCR087C-A	0.326064	0.161931	0.197226	0.189262	0.012582	-0.007517
YLR439W	MRPL4	0.310079	0.081827	0.197116	0.010747	0.162187	0.17359
YDL229W	SSB1	0.169903	0.249711	0.195167	0.126598	-0.221744	-0.301591
YJL126W	NIT2	0.041905	0.026339	0.194958	-0.413473	0.100324	0.167412
YCR082W	YCR082W	0.309568	0.140988	0.194379	0.113402	0.037419	0.167674
YKR039W	GAP1	0.034291	0.017228	0.193433	0.250696	0.385729	0.335838
YHL033C	RPL8A	0.126083	0.322345	0.191937	0.135577	0.008316	-0.154145

YOR205C	YOR205C	0.367604	0.175997	0.190378	0.244348	0.139994	0.040025
YMR038C	LYS7	0.12706	0.109056	0.189611	0.11665	0.068746	0.311013
YFR011C	YFR011C	0.197836	0.166131	0.188043	0.400277	-0.037109	0.08973
YLR008C	YLR008C	0.441419	0.281119	0.187568	0.301799	0.127329	0.097794
YLR320W	YLR320W	0.549753	0.322255	0.187022	0.248131	0.187464	0.216373
YOR063W	RPL3	0.253667	0.429125	0.186333	0.356167	-0.096079	-0.009515
YBL080C	PET112	0.131155	0.112848	0.185487	0.089025	0.192252	0.325039
YHR143W-A	RPC10	0.170936	0.131756	0.184101	0.452903	-0.102568	-0.251224
YNR075W	COS10	-0.496211	-0.204176	0.183801	0.098281	-0.119954	0.010447
YEL076C-A	YEL076C-A	-0.064799	0.063701	0.180551	-0.019464	-0.531739	NaN
YOL144W	NOP8	0.044294	0.109949	0.179696	0.214662	-0.432599	-0.136406
YGR103W	YGR103W	0.354949	0.259969	0.17746	0.131219	-0.308592	-0.083765
YHR165C	PRP8	0.325081	0.174812	0.177005	0.034487	0.198109	0.000546
YDR237W	MRPL7	0.304814	0.215449	0.176847	0.174145	0.169324	0.200059
YEL026W	SNU13	0.252211	0.324496	0.175848	0.527847	-0.077387	-0.453627
YHR072W-A	NOP10	0.20662	0.019751	0.175799	0.360229	-0.150368	-0.201597
YLR202C	YLR202C	0.298358	0.329852	0.175271	0.16784	0.154826	0.412339
YLR222C	YLR222C	0.239746	0.198191	0.175158	0.187056	-0.207042	-0.314692
YGL070C	RPB9	-0.014638	0.110223	0.174969	0.428269	0.151943	-0.273412
YPL029W	SUV3	0.390286	0.232318	0.174094	0.200445	0.233205	0.073564
YFL016C	MDJ1	-0.130865	-0.029944	0.170805	-0.010689	0.255328	0.391441
YHL038C	CBP2	0.103249	0.16643	0.168908	0.147459	0.158864	0.405092
YNR040W	YNR040W	0.231517	0.215747	0.168448	0.314283	0.11873	0.083594
YOR341W	RPA190	0.290938	0.207676	0.167063	0.083741	-0.240195	-0.325217
YCR072C	YCR072C	0.183589	0.119102	0.166975	0.294307	-0.293699	-0.55938
YML091C	RPM2	-0.048287	0.098069	0.16674	0.057195	0.029768	0.944405
YGL033W	HOP2	0.086399	0.390918	0.166462	-0.124618	-0.16967	-0.101352
YNL306W	YNL306W	0.191163	0.302045	0.160297	0.088325	0.078171	0.265469
YBL070C	YBL070C	0.076703	0.062979	0.16018	-0.411915	0.116163	-0.084079
YDR375C	BCS1	0.262286	0.171036	0.158622	0.046654	0.127053	0.31112
YIL170W	HXT12	-0.104155	0.010532	0.157944	0.047097	-0.3393	0.020931
YFR057W	YFR057W	0.331053	0.000924	0.157831	0.072638	0.162512	-0.22702
YDR268W	MSW1	0.278469	0.236438	0.157728	0.421869	0.10211	0.182139
YOR268C	YOR268C	0.199964	0.167923	0.153591	0.300512	0.020727	0.402438
YFR026C	YFR026C	0.094126	-0.462255	0.153246	0.297581	0.244154	0.287266
YHR052W	YHR052W	0.113274	0.140603	0.152085	0.011435	-0.204097	-0.30441
YOR340C	RPA43	0.112428	0.129406	0.151122	0.389512	-0.141106	-0.117095
YPR137W	RRP9	0.018691	0.0817	0.149741	0.09335	-0.124395	-0.377323
YGR272C	YGR272C	-0.078886	-0.115925	0.148806	0.230824	-0.352318	-0.437002
YGR035C	YGR035C	-0.147231	0.240957	0.148618	0.161401	0.169654	-0.365426
YML093W	YML093W	0.244755	0.172798	0.146942	0.106284	-0.126698	-0.304818
YLR248W	RCK2	0.047038	0.196576	0.14602	-0.034215	0.277726	0.412852
YKL040C	NFU1	0.19173	0.127152	0.145846	0.06043	0.052613	0.341607
YPL211W	NIP7	0.376078	0.247295	0.145734	0.167115	-0.312029	-0.324582
YBR029C	CDS1	0.196875	0.168023	0.144406	0.141416	-0.096681	-0.308424
YLR382C	NAM2	0.295492	0.204599	0.144216	0.22781	0.189678	0.408384
YDR020C	YDR020C	0.171275	-0.047501	0.143247	0.11052	-0.031715	-0.314027
YLR172C	DPH5	0.318319	0.127921	0.142431	0.021144	-0.040788	-0.124909
YDR347W	MRP1	0.320995	0.248424	0.14237	0.165683	0.066247	0.189943
YLR129W	DIP2	0.192399	0.157603	0.141538	0.078711	-0.129299	-0.453444
YAL046C	YAL046C	0.202753	0.149423	0.13989	0.32157	0.020912	-0.082961
YOR246C	YOR246C	0.276434	0.083528	0.139389	0.376032	0.211162	-0.123584

YPL021W	ECM23	0.127292	0.037915	0.137477	0.104476	-0.143383	-0.460136
YJR122W	CAF17	0.335805	0.254947	0.136725	0.296328	0.286795	0.551405
YNL135C	FPR1	0.176566	0.1784	0.13546	0.007681	0.345498	0.02877
YIR011C	STS1	0.070323	-0.160712	0.135134	0.310515	0.152812	0.032078
YNL142W	MEP2	0.053371	-0.055434	0.134617	0.363005	-0.01754	0.069797
YER110C	KAP123	0.300746	0.094971	0.134546	-0.021684	-0.108347	-0.387236
YGR123C	PPT1	0.034773	-0.010017	0.134407	0.167571	-0.358698	-0.379792
YNR074C	YNR074C	0.030446	0.022868	0.132657	0.199221	0.002592	0.355406
YNL030W	HHF2	-0.018548	0.022814	0.131138	0.242825	0.224364	-0.345858
YHL020C	OPI1	-0.002138	-0.008061	0.130929	0.114822	0.304033	0.143897
YCR026C	YCR026C	0.169001	0.056203	0.130551	0.017975	0.131896	0.306704
YAR008W	SEN34	0.10988	0.057402	0.13041	0.142107	0.057789	-0.412627
YBR284W	YBR284W	-0.316745	-0.035436	0.129764	0.007723	-0.003843	-0.004431
YKR006C	MRPL13	0.329549	0.334264	0.129667	0.190416	0.076557	0.279616
YJL046W	YJL046W	0.091407	0.121409	0.129218	0.219439	0.002214	0.30338
YCR051W	YCR051W	0.301697	0.015926	0.128523	0.162398	0.032952	-0.008497
YEL054C	RPL12A	0.105508	0.32091	0.128349	0.13464	-0.110517	-0.138007
YBL045C	COR1	0.182227	0.119657	0.126831	-0.087326	0.166422	0.345594
YNL247W	YNL247W	0.36948	0.19265	0.126787	0.114228	0.03698	-0.076981
YDR194C	MSS116	0.246308	0.32652	0.126712	0.105116	0.148195	0.226136
YEL020W-A	TIM9	0.015725	-0.011754	0.122642	0.290607	-0.120994	-0.341197
YKL149C	DBR1	0.081321	-0.060512	0.122007	-0.098039	0.310106	0.086877
YNL175C	NOP13	0.326304	0.217455	0.121888	0.075438	-0.08923	-0.147124
YPL093W	NOG1	0.229778	0.129658	0.120615	-0.00633	-0.348272	-0.368517
YBR009C	HHF1	0.047363	-0.044895	0.120465	0.090865	0.234959	-0.323019
YLR056W	ERG3	0.526349	0.034641	0.120306	0.078624	0.066916	-0.182895
YKL078W	YKL078W	0.26131	0.032219	0.118702	0.070148	-0.174717	-0.340937
YBR121C	GRS1	0.302011	0.121329	0.118	0.251727	0.069809	-0.069791
YIR032C	DAL3	-0.02696	-0.030104	0.117101	0.320986	-0.123758	-0.114139
YMR318C	YMR318C	0.174256	0.202819	0.115581	0.427706	0.054251	0.209176
YHR174W	ENO2	-0.154948	-0.302524	0.115494	-0.13581	-0.219045	-0.290744
YPL252C	YAH1	0.33212	0.328742	0.115039	0.256605	0.074274	0.245076
YPR124W	CTR1	-0.081436	0.000433	0.114604	-0.315618	-0.349034	0.109514
YDL039C	PRM7	0.479961	0.287138	0.113736	0.204014	-0.280668	-0.188933
YDL181W	INH1	0.06687	0.026068	0.112462	-0.213865	0.178247	0.424154
YPL174C	NIP100	0.190933	0.037967	0.111807	0.117114	0.168979	0.328119
YDR106W	ARP10	0.121646	0.109385	0.111139	0.119223	0.313077	0.057156
YLR214W	FRE1	-0.180476	0.068054	0.110818	0.174057	-0.067418	0.765663
YEL010W	YEL010W	-0.164756	0.006494	0.108279	-0.175033	0.389683	0.171349
YBR067C	TIP1	0.227311	0.365987	0.108262	0.045602	0.647124	0.40052
YGL129C	RSM23	0.130114	0.129848	0.106437	0.105859	0.104975	0.356701
YIL042C	YIL042C	0.156515	-0.023778	0.106007	-0.048577	0.258948	0.319191
YLR428C	YLR428C	-0.013845	-0.064929	0.105761	0.071464	0.007187	0.3311
YCL049C	YCL049C	0.036965	0.107799	0.105634	0.078311	-0.335615	-0.028445
YLR259C	HSP60	0.0201	0.118436	0.105464	0.111803	0.390316	0.371709
YMR146C	TIF34	0.390856	0.153114	0.104694	0.116922	0.002155	-0.03573
YPL059W	GRX5	0.23437	0.130456	0.103777	0.024655	0.063021	0.394047
YLR249W	YEF3	0.117957	0.160257	0.102971	0.263551	-0.362401	-0.424962
YKL043W	PHD1	0.01554	0.048204	0.102383	-0.073494	0.194733	0.301779
YHR184W	SSP1	-0.040334	-0.006256	0.101175	-0.317236	-0.048539	-0.053418
YJL109C	YJL109C	0.207325	0.105064	0.100974	0.12148	-0.136314	-0.358824
YER119C-A	YER119C-A	-0.308089	-0.281027	0.099197	-0.247998	0.039292	0.265128

YHR025W	THR1	0.305194	0.161316	0.098953	0.139014	0.255427	-0.020028
YHR157W	REC104	0.080983	0.02532	0.098451	0.039346	0.401137	0.127401
YGL118C	YGL118C	-0.437892	-0.117459	0.098289	0.288379	NaN	0.000466
YOR095C	RKI1	-0.037546	-0.048949	0.097738	0.040314	0.045466	-0.329105
YIL006W	YIL006W	0.284112	0.154359	0.09682	0.173864	0.265827	0.494425
YOR256C	YOR256C	-0.097403	0.035084	0.096251	0.069899	0.1044	0.393581
YDR309C	GIC2	0.206068	0.107823	0.095825	0.125448	0.232935	0.315427
YDR462W	MRPL28	0.346539	0.182662	0.095469	0.116303	0.108752	0.188129
YNL079C	TPM1	0.305713	0.152494	0.094262	0.199407	0.106166	0.01669
YNL170W	YNL170W	-0.16725	-0.157749	0.094038	0.413908	0.127092	0.17937
YKR051W	YKR051W	-0.025472	0.005263	0.092725	-0.016572	0.201387	0.527794
YJR136C	YJR136C	0.105102	0.120813	0.092676	0.062824	0.343248	0.206539
YDR513W	TTR1	-0.143602	-0.037759	0.091265	-0.167267	-0.088076	0.38339
YJR091C	JSN1	-0.317441	-0.321218	0.090607	-0.225204	-0.133799	0.308199
YLL011W	SOF1	-0.033666	0.06592	0.089554	0.189798	-0.103369	-0.358679
YPR154W	YPR154W	-0.215196	0.027375	0.089168	0.020338	0.339266	0.279852
YML022W	APT1	0.022259	0.243748	0.088916	0.124532	-0.27877	-0.400544
YIR026C	YVH1	0.357482	0.091134	0.088042	0.056464	0.088022	-0.009268
YNL156C	YNL156C	-0.001394	0.003536	0.086374	0.003953	0.037277	0.311383
YCL065W	YCL065W	-0.091396	-0.156	0.086046	-0.307265	-0.044102	-0.124216
YOR288C	MPD1	-0.574659	-0.065096	0.085667	-0.077971	-0.31059	0.02559
YDR091C	RLI1	0.077124	0.083516	0.085632	0.059976	-0.19378	-0.503634
YOR354C	YOR354C	0.345137	0.230816	0.08554	0.164054	0.161541	0.276741
YDL060W	YDL060W	0.111916	0.080115	0.084828	0.098842	-0.275539	-0.463289
YPR126C	YPR126C	0.314014	0.152557	0.084603	0.093095	0.642215	0.213261
YOL031C	YOL031C	-0.329874	-0.007094	0.084409	-0.048519	0.030128	0.114062
YJR074W	MOG1	0.122752	0.146361	0.084407	-0.090446	0.046025	-0.303399
YOR045W	TOM6	0.405141	0.395147	0.082101	0.392701	0.135065	0.092803
YGR293C	YGR293C	0.016814	-0.012991	0.082018	-0.221304	NaN	0.311414
YOR059C	YOR059C	0.088667	0.098793	0.081784	0.049591	0.125784	0.379723
YNL008C	YNL008C	0.012667	0.092432	0.080365	0.013696	0.385084	0.075176
YJR153W	PGU1	-0.038706	0.108105	0.079109	-0.409697	-0.004827	-0.040724
YDL136W	RPL35B	0.376362	0.105072	0.078982	0.18955	-0.059798	-0.046744
YGL263W	COS12	0.137031	-0.139607	0.078321	0.344506	0.005825	-0.084569
YLL014W	YLL014W	-0.038699	0.115033	0.078314	0.378959	0.051698	-0.153399
YPR040W	YPR040W	0.084694	-0.0486	0.077087	-0.205098	0.05789	-0.308114
YJL011C	YJL011C	0.072151	0.016699	0.076009	0.068942	0.004866	-0.345218
YOR359W	YOR359W	-0.052196	0.145812	0.073867	0.287875	-0.177044	-0.634791
YDR036C	YDR036C	0.19882	0.196808	0.073667	0.09072	0.045019	0.327831
YOR145C	YOR145C	0.04547	-0.020327	0.072567	-0.068745	-0.155381	-0.323317
YLR168C	MSF1'	-0.075314	0.03519	0.071313	0.275855	0.15397	0.364243
YGR128C	YGR128C	0.22174	0.14299	0.070541	-0.00091	-0.222494	-0.360862
YOR226C	ISU2	-0.074986	0.06049	0.070394	-0.238899	-0.069533	0.503056
Q0130	OLI1	0.264305	0.352817	0.070343	0.043762	0.48958	0.033055
YOR147W	YOR147W	-0.001207	0.080095	0.07029	0.064394	0.130663	0.415496
YDR472W	TRS31	0.098275	-0.004596	0.069519	0.038863	0.004127	0.301755
YOL036W	YOL036W	0.021871	-0.062205	0.069404	0.012953	0.235082	0.337535
YDR114C	YDR114C	-0.00897	0.099764	0.06863	-0.560034	0.29116	-0.077865
YKL216W	URA1	-0.206532	0.084441	0.068264	0.120021	-0.086012	-0.385771
YNL037C	IDH1	-0.255443	-0.019057	0.066847	0.051977	-0.119866	0.403033
YJL010C	YJL010C	0.07341	-0.014022	0.066818	0.097883	-0.157207	-0.313568
YML120C	NDI1	-0.032108	-0.067173	0.066652	-0.033808	-0.000977	0.409729

YLR090W	XDJ1	0.062328	0.024175	0.065133	0.018038	0.417473	0.182468
YPR010C	RPA135	0.456909	0.164784	0.064725	0.092345	-0.391635	-0.40864
YOR172W	YOR172W	-0.177319	-0.109364	0.064695	0.016882	0.076046	0.344022
YDR216W	ADR1	-0.092031	0.173725	0.064431	-0.174862	0.204374	0.419656
YPL241C	CIN2	0.31835	0.12804	0.063915	0.024958	0.255603	-0.075867
YCL068C	YCL068C	-0.108921	0.025004	0.063434	-0.41626	0.077883	0.186669
YHR085W	YHR085W	0.041968	0.029759	0.062947	0.080353	-0.129922	-0.46259
YOR346W	REV1	0.05722	0.129199	0.062909	0.065757	0.030009	-0.305885
YBR050C	REG2	0.130458	-0.014665	0.061961	-0.408865	0.067349	-0.08948
YKL056C	YKL056C	0.164876	0.349689	0.061528	-0.051947	0.032249	-0.343
YCL026C-A	FRM2	-0.019329	-0.093783	0.060748	-0.097233	0.047897	0.726058
YCR079W	YCR079W	0.042291	-0.037925	0.059982	-0.05029	0.055025	0.338039
YNL172W	APC1	0.052469	0.0973	0.059168	0.100892	0.070565	-0.409247
YGR210C	YGR210C	0.019641	0.074992	0.05826	-0.079311	0.40836	-0.047993
YMR201C	RAD14	0.017224	-0.060143	0.056804	0.000571	0.35721	0.103186
YDL122W	UBP1	-0.008271	0.12429	0.056689	-0.012005	-0.076607	0.341935
YLR348C	DIC1	0.051042	0.017002	0.056439	0.060227	0.109639	-0.405556
YBL075C	SSA3	-0.103645	0.070662	0.051975	-0.368447	0.323008	0.229399
YMR093W	YMR093W	0.066376	0.039158	0.05168	0.173301	-0.077975	-0.365208
YLR336C	SGD1	0.164172	0.072663	0.051372	0.017996	0.026981	-0.312064
YKL221W	YKL221W	-0.01463	-0.031959	0.051102	-0.514881	-0.218654	-0.055941
YDR413C	YDR413C	0.045403	0.055903	0.049769	-0.027227	0.423607	0.042829
YJR044C	YJR044C	-0.066419	0.106753	0.049423	0.069406	-0.385702	0.091873
YOR328W	PDR10	0.03377	-0.016514	0.04916	-0.392336	-0.114815	0.175362
YOL081W	IRA2	-0.069589	0.077012	0.049044	-0.271083	0.311893	0.005122
YKL029C	MAE1	0.021637	0.01314	0.048661	0.068281	-0.049139	-0.311007
YBR010W	HHT1	-0.012733	-0.024648	0.04866	-0.001954	0.129024	-0.313099
YBL062W	YBL062W	-0.144917	0.053874	0.048343	-0.335943	-0.154118	-0.248231
YDR399W	HPT1	0.147462	-0.03172	0.047879	-0.001483	-0.221104	-0.493357
YOL080C	REX4	0.008286	0.121924	0.047852	0.037756	-0.244142	-0.528032
YMR316W	DIA1	-0.417106	-0.148431	0.047123	-0.067095	0.085232	-0.108169
YMR067C	YMR067C	-0.16778	-0.08298	0.046644	-0.356123	-0.030953	0.116582
YGR112W	SHY1	0.124384	-0.000507	0.045469	0.142249	-0.070652	0.379634
YIL151C	YIL151C	-0.312611	-0.109463	0.044028	0.169922	0.119314	-0.065388
YGL218W	YGL218W	-0.005653	-0.062178	0.044028	-0.440689	0.128853	0.046577
YPL016W	SWI1	-0.153408	-0.068126	0.043822	0.01681	-0.195631	-0.417586
YGR225W	AMA1	-0.127516	-0.075385	0.043513	-0.442163	-0.260524	0.412669
YJL101C	GSH1	0.00524	-0.013716	0.043236	0.032191	0.014486	0.330236
YCR024C	YCR024C	-0.029363	0.097993	0.041593	0.13109	-0.022541	0.405048
YDL091C	YDL091C	-0.157399	-0.199213	0.041306	-0.110523	0.016366	0.480956
YKR026C	GCN3	0.014476	0.085728	0.039174	-0.504387	0.132186	0.432505
YKL201C	MNN4	-0.094886	-0.162373	0.036875	-0.118951	0.392624	0.096321
YLR092W	SUL2	-0.085113	-0.158356	0.036815	0.205899	0.203355	0.312695
YMR085W	YMR085W	-0.389835	-0.042289	0.036021	0.014574	0.058106	0.447271
YJR045C	SSC1	0.026152	0.215108	0.035854	-0.0124	0.570292	0.312889
YBL002W	HTB2	0.020395	0.035118	0.034528	-0.059758	0.026644	-0.337101
YCR043C	YCR043C	0.157243	0.213463	0.032567	0.339659	0.055214	-0.338128
YNL323W	LEM3	-0.037017	0.064744	0.031321	-0.591009	0.028848	-0.016309
YDR353W	TRR1	0.071984	0.157626	0.029553	-0.130232	-0.06253	0.555755
YGR236C	SPG1	-0.279664	-0.088285	0.029271	-0.387618	-0.13225	-0.088624
YBR201W	DER1	-0.3108	-0.048836	0.027225	0.04437	-0.062453	0.047061
YIL110W	YIL110W	-0.017421	0.019431	0.026783	0.075897	0.029573	-0.421354

YLR003C	YLR003C	0.1145	-0.021288	0.026541	0.013478	-0.158702	-0.321585
YGR087C	PDC6	0.118785	0.055698	0.023909	-0.381399	-0.100391	-0.196368
YMR084W	YMR084W	-0.536712	-0.012587	0.023616	-0.077246	0.049252	0.438103
YDR488C	PAC11	-0.212443	-0.225131	0.023608	-0.180604	-0.087965	0.347059
YOR220W	YOR220W	-0.148261	-0.022209	0.022425	-0.249512	0.052022	0.389223
YDL069C	CBS1	-0.050729	-0.080718	0.022015	0.031281	0.081675	0.325506
YEL069C	HXT13	-0.418716	-0.120974	0.020658	-0.201421	-0.226465	-0.039936
YOR305W	YOR305W	0.067467	0.115195	0.020185	0.395697	0.127439	-0.071943
YHR096C	HXT5	-0.516128	-0.341847	0.018874	-0.397885	-0.527164	0.126344
YDR324C	YDR324C	0.038751	0.054634	0.018858	0.048912	-0.201387	-0.313351
YJR105W	ADO1	0.310087	0.246422	0.018818	0.183503	0.120295	-0.032136
YKR066C	CCP1	0.071	-0.115511	0.017856	0.018291	0.048133	0.778088
YML130C	ERO1	-0.275468	-0.128449	0.017464	-0.003868	0.372661	0.409956
YBR090C	YBR090C	-0.168481	-0.024875	0.017027	0.069273	-0.295986	-0.336728
YOR116C	RPO31	0.093337	0.049888	0.016914	0.069257	-0.040788	-0.517169
YPR065W	ROX1	-0.099632	0.023969	0.015686	-0.090484	-0.052307	0.353479
YDL035C	GPR1	0.042865	-0.005347	0.01481	-0.053184	0.082397	0.493414
YPL083C	SEN54	0.326566	0.234054	0.014078	0.026607	-0.148031	-0.288394
YPR174C	YPR174C	-0.007883	NaN	0.013937	0.089944	0.146431	0.340894
YKR079C	YKR079C	0.288652	0.306314	0.013397	0.110465	-0.220487	-0.120506
YOR108W	YOR108W	0.136122	0.103269	0.013236	-0.303049	0.019788	-0.124995
YJL036W	SNX4	-0.173003	-0.148304	0.013073	-0.210781	0.021141	0.317068
YER044C-A	MEI4	-0.158737	-0.16537	0.013052	-0.254138	0.323774	0.382435
YNL331C	AAD14	0.028632	-0.205599	0.012517	0.076538	0.20151	0.345583
YBL024W	NCL1	0.050511	0.121759	0.012259	-0.013269	-0.081577	-0.333326
YGL050W	YGL050W	-0.181839	-0.081587	0.011479	-0.010278	-0.129987	-0.348764
YPL012W	YPL012W	0.183662	0.093917	0.010922	-0.003481	-0.284767	-0.367493
YKL097C	YKL097C	-0.101225	0.051306	0.010715	NaN	0.064785	0.481054
YGR139W	YGR139W	-0.034425	0.076338	0.010303	0.00133	0.126411	0.373669
YGR011W	YGR011W	-0.041072	0.009584	0.009822	0.041995	0.004863	0.358417
YOR170W	YOR170W	-0.330323	-0.170415	0.007774	-0.251138	0.248303	0.252904
YGR159C	NSR1	0.125098	0.015314	0.007591	0.106305	-0.293048	-0.398479
YJL214W	HXT8	-0.340828	-0.177008	0.007497	-0.340728	-0.220832	-0.368801
YGR052W	YGR052W	-0.419576	-0.080748	0.006808	-0.711218	-0.067951	0.299511
YNL014W	YNL014W	0.048281	0.131681	0.005439	-0.073839	-0.354981	-0.536659
YMR184W	YMR184W	-0.351432	-0.098539	0.004428	0.138739	-0.091164	-0.041029
YLR054C	YLR054C	-0.166724	-0.421527	0.004317	0.043566	-0.120233	0.019243
YGR046W	YGR046W	-0.027114	-0.042104	0.00381	-0.081421	0.11379	0.380217
YNL141W	AAH1	0.198288	-0.00685	0.003705	0.243424	-0.346698	-0.646859
YLR163C	MAS1	0.008362	0.147699	0.003295	-0.113149	0.059767	0.376985
YMR053C	STB2	-0.029641	0.057521	0.003169	0.002394	0.15171	0.359897
YMR304C-A	YMR304C-A	0.154036	-0.108046	0.00315	0.169618	0.339936	-0.093383
YLR184W	YLR184W	0.116809	0.349361	0.002757	0.098315	0.092902	-0.007791
YEL017W	YEL017W	0.093679	0.022441	0.002297	0.160997	-0.056649	-0.339355
YOR044W	YOR044W	-0.21421	0.051474	0.002167	0.187659	0.328614	0.381088
YHR144C	DCD1	-0.099732	0.019063	0.00057	0.190079	-0.209641	-0.577662
YOR360C	PDE2	-0.17318	0.0575	0.00037	0.06856	-0.033342	0.302318
YGR090W	YGR090W	-0.016359	0.039996	0.000032	-0.053257	-0.174013	-0.343866
YER189W	YER189W	-0.367596	0.025985	-0.000223	0.06272	-0.175802	-0.175706
YHR019C	DED81	0.302325	0.323684	-0.000408	0.347408	0.18895	-0.064126
YML131W	YML131W	-0.110058	-0.00983	-0.001666	-0.027099	0.065267	0.630732
YLR414C	YLR414C	-0.362198	-0.235178	-0.002079	0.028555	0.025646	0.358066

YPR202W	YPR202W	-0.321175	0.034988	-0.002351	0.136314	-0.089153	-0.16742
YBL085W	BOI1	-0.043684	-0.04492	-0.006158	0.101602	-0.029829	0.309821
YKL193C	SDS22	0.022406	-0.063159	-0.006264	-0.181192	-0.028791	0.304057
YML080W	YML080W	0.107501	0.08787	-0.006618	0.051466	-0.071586	-0.329967
YHL036W	MUP3	-0.40042	-0.133256	-0.008001	0.050232	0.095938	0.690502
YDL200C	MGT1	-0.036525	-0.103641	-0.010462	-0.151038	-0.11909	0.303466
YLR155C	ASP3-1	0.425533	0.366714	-0.011449	0.413369	0.464942	0.214006
YBR011C	IPP1	0.315029	0.074675	-0.012489	0.030013	0.205546	0.046481
YBR008C	FLR1	0.223574	0.062511	-0.012582	-0.00812	0.230318	0.898917
YPL168W	YPL168W	0.079268	0.039435	-0.012606	-0.016093	0.211076	0.38801
YCR020C-A	MAK31	0.088848	0.164817	-0.012765	0.322431	0.063245	-0.08722
YOL123W	HRP1	0.033787	0.021612	-0.014153	0.022691	0.140525	-0.306833
YDR154C	YDR154C	-0.163528	-0.348843	-0.014895	-0.102069	0.014793	-0.074901
YLR307W	CDA1	-0.01317	-0.103354	-0.015771	-0.292554	-0.357112	-0.084871
YOR186W	YOR186W	-0.022121	0.032968	-0.017205	-0.42981	0.002026	-0.115853
YDR342C	HXT7	-0.345643	-0.077958	-0.018273	-0.092415	-0.257161	-0.066983
YBR199W	KTR4	0.057243	-0.02219	-0.018306	-0.091415	0.094224	0.320066
YDL110C	YDL110C	0.00142	-0.174094	-0.018775	-0.100882	-0.12835	0.451635
YDR256C	CTA1	0.064445	0.011049	-0.019646	-0.151369	0.14833	0.316033
YFL057C	YFL057C	-0.091855	-0.146615	-0.020661	0.04628	0.149996	0.398236
YOR379C	YOR379C	0.007937	0.383212	-0.021544	0.133346	0.048522	0.202414
YMR032W	HOF1	-0.060547	0.160681	-0.021969	0.814229	0.243084	-0.20414
YDR504C	YDR504C	-0.065198	0.054399	-0.02268	-0.433343	-0.025219	0.018257
YLR350W	YLR350W	-0.141682	-0.162971	-0.022916	-0.308067	0.019287	-0.006299
YEL075C	YEL075C	-0.345386	0.033844	-0.02292	0.160691	-0.138619	-0.049874
YJL153C	INO1	-0.273458	-0.367242	-0.023566	-0.2223	-0.072583	-0.039224
YDR224C	HTB1	-0.000159	0.019593	-0.024994	0.008694	-0.011891	-0.306444
YGL245W	YGL245W	0.39688	0.117213	-0.025323	0.260221	0.334606	0.105244
YKL027W	YKL027W	-0.106967	-0.053971	-0.025578	-0.065972	-0.096316	-0.316655
YBR089C-A	NHP6B	-0.091253	-0.054123	-0.026181	0.045316	-0.257594	-0.328122
YBR034C	HMT1	0.018038	0.049686	-0.028109	-0.079563	-0.180953	-0.352916
YLL060C	GTT2	-0.034505	-0.036864	-0.028315	0.031628	0.04894	0.500057
YNL180C	RHO5	-0.003365	0.043688	-0.028438	-0.186856	-0.122007	0.366787
YHR048W	YHR048W	-0.305547	-0.123762	-0.029221	-0.077711	0.100595	0.471754
YDL025C	YDL025C	-0.057988	0.054455	-0.030578	-0.109386	0.107997	0.812474
YEL048C	YEL048C	0.058752	-0.010002	-0.030887	0.174882	-0.023935	-0.53391
YOR247W	SRL1	0.400098	0.002305	-0.031483	0.218786	0.21689	-0.179683
YNL255C	GIS2	0.130244	0.200106	-0.032534	0.0864	-0.083237	-0.307079
YNL305C	YNL305C	-0.07357	0.086106	-0.033015	-0.144224	0.075522	0.346118
YCR068W	CVT17	-0.046152	-0.027888	-0.033185	-0.131825	-0.054544	0.340946
YDR142C	PEX7	-0.048733	-0.116873	-0.033278	-0.086302	0.042241	0.324646
YNL134C	YNL134C	-0.076736	-0.050246	-0.034875	-0.171353	0.057638	0.531816
YNR050C	LYS9	0.328995	0.254423	-0.036014	0.013813	0.082933	0.040529
YAL061W	YAL061W	-0.276301	0.037622	-0.036784	-0.304064	-0.138284	0.003666
YLR081W	GAL2	-0.322574	-0.151983	-0.037406	-0.139153	-0.232713	0.036033
YGR069W	YGR069W	-0.217675	-0.234982	-0.038144	-0.172712	-0.401959	-0.243948
YDR072C	IPT1	-0.382037	-0.116201	-0.039158	-0.045424	-0.038942	0.037296
YNL077W	YNL077W	-0.347764	-0.043126	-0.039494	-0.262099	0.256622	0.02509
YKR067W	YKR067W	-0.071944	0.017822	-0.040795	0.032684	0.019898	0.503535
YOR058C	ASE1	-0.134497	0.029296	-0.042041	0.01483	0.097311	0.323627
YLR216C	CPR6	-0.088195	-0.031438	-0.042257	-0.112403	0.265192	0.405439
YPL187W	MF(ALPHA)1	0.23594	0.296712	-0.04307	-0.442995	0.019251	-0.083674

YCR014C	POL4	-0.148839	-0.070958	-0.043102	-0.320228	-0.106577	-0.216155
YDR096W	GIS1	-0.318779	-0.104822	-0.046169	-0.327458	-0.190343	0.065445
YBL018C	POP8	0.312595	0.117246	-0.047546	0.091454	0.108559	-0.145009
YPR012W	YPR012W	-0.422683	0.009236	-0.048572	0.063657	-0.040508	-0.04909
YDL231C	BRE4	0.303683	0.081916	-0.049721	0.105791	0.111617	0.127203
YOL032W	YOL032W	-0.292071	-0.139576	-0.049804	-0.134062	0.340418	0.618318
YKR080W	MTD1	0.441098	-0.126821	-0.051546	-0.090098	-0.439111	-0.17598
YGL053W	PRM8	-0.120521	-0.12503	-0.05207	-0.056917	-0.015064	0.361429
YOR342C	YOR342C	0.074993	0.042886	-0.052518	-0.026374	-0.088493	-0.308629
YMR140W	YMR140W	-0.021985	0.020835	-0.052757	0.026127	-0.028504	0.469028
YEL012W	UBC8	-0.340884	-0.28272	-0.053042	-0.189584	-0.261603	0.052773
YDL020C	RPN4	-0.015719	-0.137772	-0.053109	-0.264955	0.215017	0.389432
YFL031W	HAC1	-0.308817	-0.126281	-0.053553	-0.069972	-0.00756	-0.132297
YOR381W	FRE3	-0.047541	0.383312	-0.055015	0.163545	0.08634	0.161855
YIR016W	YIR016W	0.041938	-0.080744	-0.057161	0.136932	0.084893	0.350964
YMR081C	ISF1	0.157811	0.020629	-0.058218	-0.370453	-0.036691	-0.009268
YIL168W	SDL1	-0.096123	-0.079437	-0.058749	-0.061444	-0.042011	0.368739
YHR049W	YHR049W	0.140506	0.081608	-0.058981	0.189697	0.536553	-0.033854
YPL279C	YPL279C	0.115041	0.08278	-0.059386	0.054636	-0.13071	-0.40648
YBR230C	YBR230C	-0.164941	-0.102228	-0.059644	-0.214859	-0.381828	-0.10837
YDL112W	TRM3	0.080284	0.117131	-0.06078	0.080302	-0.070633	-0.312481
YBR092C	PHO3	0.214989	-0.001079	-0.062327	-0.09437	0.053778	-0.554323
YGR291C	YGR291C	-0.020503	0.315724	-0.06294	-0.079786	NaN	0.082407
YKR024C	DBP7	0.014941	0.037333	-0.063616	0.055613	-0.190156	-0.347028
YFL030W	YFL030W	-0.075013	-0.256344	-0.064827	-0.085916	-0.096256	0.474716
YGR019W	UGA1	-0.101594	-0.17309	-0.064868	-0.091695	-0.370899	0.193791
YPL057C	SUR1	-0.302952	-0.102644	-0.065774	-0.220353	0.000455	-0.264562
YDR508C	GNP1	-0.113245	-0.074276	-0.066296	-0.037488	-0.291231	-0.319465
YIL051C	MMD1	-0.105349	0.055018	-0.06733	-0.131476	-0.188212	-0.682573
YIL057C	YIL057C	-0.689129	-0.274165	-0.067498	-0.057888	-0.515595	0.08628
YOR032C	HMS1	-0.013983	-0.080888	-0.067651	0.264259	0.257874	0.636906
YDR345C	HXT3	-0.313806	-0.142922	-0.06809	-0.083807	-0.221011	-0.006347
YGR015C	YGR015C	0.036891	-0.064153	-0.068171	0.169799	0.083268	0.314471
YOL014W	YOL014W	-0.605152	0.037434	-0.068585	-0.126721	-0.112731	-0.456129
YCL009C	ILV6	0.385238	0.137142	-0.06895	0.176033	0.359258	0.11751
YDR021W	FAL1	0.171062	-0.011047	-0.070196	0.048776	-0.316526	-0.187017
YKL223W	YKL223W	0.001001	-0.212367	-0.070943	-0.324942	-0.006725	-0.065252
YHR008C	SOD2	0.11062	0.055708	-0.071845	-0.077843	-0.045638	0.506083
YPL203W	TPK2	-0.209207	-0.146362	-0.072593	-0.256095	-0.112905	0.562713
YNL027W	CRZ1	-0.405185	-0.099196	-0.072737	-0.056324	0.062551	0.199831
YNR014W	YNR014W	-0.019743	-0.051556	-0.073567	-0.345087	0.065851	-0.084854
YHR179W	OYE2	-0.076192	-0.09083	-0.074157	0.102342	0.066986	0.324249
YNL195C	YNL195C	-0.199437	-0.002065	-0.074254	-0.683636	-0.330367	0.138778
YLR304C	ACO1	0.162709	0.015842	-0.074651	0.125437	-0.087574	0.523319
YCL064C	CHA1	-0.125178	-0.144342	-0.07476	-0.372015	-0.391838	-0.451586
YBR099C	YBR099C	-0.352702	-0.064147	-0.077996	-0.660114	0.445816	-0.226492
YNL064C	YDJ1	-0.3539	-0.148083	-0.078353	-0.116013	0.124833	-0.037306
YHR040W	YHR040W	-0.254648	-0.125982	-0.078608	-0.259296	-0.0158	-0.302271
YGR162W	TIF4631	0.060549	0.072731	-0.078868	0.038436	-0.209704	-0.494665
YKL120W	OAC1	-0.147844	-0.102148	-0.079222	0.141169	-0.256701	-0.311419
YOL162W	YOL162W	-0.261692	-0.286218	-0.079543	-0.216165	-0.428044	-0.106443
YDR003W	YDR003W	-0.232117	-0.092284	-0.079649	-0.139404	-0.119697	0.43564

YOL084W	PHM7	-0.263768	-0.104593	-0.080127	-0.390412	-0.101839	0.002446
YGL162W	SUT1	-0.033772	-0.013695	-0.080243	0.368143	0.003446	0.048951
YGR122W	YGR122W	-0.486187	-0.191239	-0.081579	-0.274988	-0.076363	-0.157238
YKL107W	YKL107W	0.065168	0.015889	-0.082772	-0.465257	-0.205072	-0.124259
YBR137W	YBR137W	-0.154841	-0.201024	-0.082962	-0.090256	-0.308057	-0.042294
YBR280C	YBR280C	-0.1006	-0.12847	-0.083358	-0.085962	-0.024099	0.477337
YGL157W	YGL157W	-0.162702	0.055531	-0.083541	0.398026	-0.157148	-0.012833
YOR248W	TOS11	0.272582	-0.000161	-0.083587	0.347444	0.219726	-0.101024
YGL046W	YGL046W	-0.419798	-0.097485	-0.085347	-0.154915	0.143882	0.143192
YAR047C	YAR047C	-0.037742	-0.162057	-0.08595	-0.362297	-0.129327	-0.146465
YGR256W	GND2	-0.271521	-0.156103	-0.086961	-0.555334	-0.420244	-0.176152
YBR116C	YBR116C	-0.294212	-0.000575	-0.087095	-0.725419	-0.251659	-0.002334
YKL093W	MBR1	0.059795	0.002634	-0.088471	-0.349471	-0.070041	0.001834
YNL260C	YNL260C	0.037393	-0.008973	-0.088898	0.074915	0.027386	0.556242
YGR211W	ZPR1	-0.20349	-0.049675	-0.090797	-0.152851	0.404824	0.216147
YLR068W	YLR068W	-0.393819	-0.322717	-0.091008	-0.152668	0.531148	0.311566
YEL030W	ECM10	0.025668	-0.047807	-0.091906	0.006477	0.385668	0.182218
YFR015C	GSY1	-0.334288	-0.30696	-0.092331	-0.0995	-0.090189	0.34823
YLL016W	SDC25	-0.123919	-0.078232	-0.092465	-0.309385	-0.098637	-0.170143
YIL074C	SER33	-0.027707	-0.203464	-0.093269	0.027165	-0.30672	-0.087382
YLR369W	SSQ1	-0.218403	-0.019	-0.095583	-0.315705	0.294013	0.192636
YJL165C	HAL5	-0.372813	-0.081035	-0.095853	-0.056764	0.096031	0.362518
YPL177C	CUP9	-0.106548	-0.165359	-0.09624	-0.120511	-0.341388	-0.10733
YBL053W	YBL053W	-0.169611	0.188548	-0.096861	-0.078454	-0.343856	0.017703
YNL035C	YNL035C	-0.417243	-0.122772	-0.09724	-0.338021	0.263831	0.217924
YJL012C	VTC4	0.072165	0.017251	-0.097245	0.014424	-0.318971	-0.294121
YOR067C	ALG8	-0.335799	0.030649	-0.097942	-0.035716	0.036176	-0.203601
YPL123C	RNY1	-0.004762	-0.084529	-0.098575	-0.164241	-0.125733	0.302415
YDR102C	YDR102C	0.09575	-0.1484	-0.099915	-0.334483	-0.213174	-0.272792
YPL230W	USV1	-0.163179	-0.088174	-0.101015	-0.346411	-0.209153	0.054776
YBR033W	YBR033W	-0.305129	-0.104252	-0.10121	-0.205646	-0.165598	-0.033577
YDR516C	YDR516C	-0.306944	-0.306065	-0.101527	-0.198813	-0.233903	0.179182
YER035W	EDC2	-0.253154	-0.125202	-0.102426	-0.302957	0.161942	0.29011
YGL051W	YGL051W	0.467799	-0.0698	-0.102776	0.060921	-0.019678	-0.04455
YKL060C	FBA1	-0.121309	-0.179828	-0.105285	-0.303516	-0.249309	-0.245563
YOR355W	GDS1	0.193084	0.114586	-0.105514	0.016787	-0.518942	-0.364646
YCL042W	YCL042W	-0.330099	-0.222029	-0.105841	-0.269728	-0.26912	0.189992
YGR022C	YGR022C	0.071182	0.059053	-0.106473	-0.376443	0.022159	0.003899
YLR377C	FBP1	0.02679	-0.009579	-0.106691	-0.372731	-0.015363	0.016508
YAL034W-A	MTW1	-0.403506	-0.352664	-0.108913	-0.258567	-0.28225	0.259141
YPL126W	NAN1	0.551951	0.185831	-0.110645	-0.019081	-0.190138	-0.118879
YOR030W	DFG16	0.079995	0.126254	-0.110902	-0.09985	0.137092	0.390835
YHR032W	YHR032W	-0.170915	-0.077605	-0.111101	-0.053597	-0.080069	-0.400482
YKL071W	YKL071W	-0.125155	-0.030621	-0.111713	-0.138598	-0.081307	0.308074
YGR194C	XKS1	-0.139923	-0.092097	-0.112671	-0.100242	0.032349	0.342846
YLR083C	EMP70	0.085533	0.040499	-0.112877	0.108036	-0.075117	-0.552892
YDR343C	HXT6	-0.469519	-0.126408	-0.11333	-0.118542	-0.281139	-0.103141
YGR242W	YGR242W	-0.11452	-0.150305	-0.11392	-0.361881	-0.145068	0.106184
YNR061C	YNR061C	-0.338397	-0.002396	-0.114593	-0.091345	-0.004831	-0.156837
YKL086W	YKL086W	-0.12034	-0.132373	-0.114926	-0.008934	-0.15898	0.942376
YNL036W	NCE103	0.02912	0.1642	-0.116704	-0.023525	-0.13245	0.836639
YCL073C	YCL073C	-0.049489	-0.051735	-0.119559	-0.327872	0.016475	-0.141759

YDL180W	YDL180W	-0.228404	-0.32074	-0.119666	-0.259061	-0.147622	0.085018
YER072W	VTC1	-0.064746	0.04352	-0.119985	-0.035811	-0.298678	-0.319614
YLL056C	YLL056C	-0.096574	-0.061887	-0.120307	-0.109816	0.036472	0.625288
YOR049C	YOR049C	-0.533789	0.105945	-0.120607	-0.385082	-0.073976	-0.003443
YDR069C	DOA4	-0.095407	-0.047712	-0.120975	-0.21027	-0.042868	0.755052
YNL212W	VID27	-0.359877	-0.103692	-0.121321	-0.169542	-0.141098	0.035255
YBL033C	RIB1	-0.258238	-0.164398	-0.122466	-0.092469	-0.082695	0.36961
YPL223C	GRE1	-0.049348	-0.183053	-0.123755	-0.591245	-0.017223	0.155626
YGL037C	PNC1	-0.166839	-0.316271	-0.123822	-0.335677	-0.092732	0.283428
YLR180W	SAM1	-0.08458	-0.173046	-0.124838	0.125704	-0.263815	-0.356472
YPL171C	OYE3	-0.10448	-0.110666	-0.126689	-0.067942	-0.037952	0.668037
YCR012W	PGK1	-0.300528	-0.420348	-0.126821	-0.042095	-0.176437	-0.158363
YNL259C	ATX1	-0.293412	-0.476871	-0.126906	0.156387	-0.415051	-0.415652
YKL026C	GPX1	-0.361361	-0.287579	-0.129723	-0.265039	-0.373963	-0.011741
YDR151C	CTH1	-0.23968	-0.069473	-0.130134	-0.132093	0.292509	0.540045
YDR001C	NTH1	-0.041704	-0.11009	-0.130477	-0.148838	0.018227	0.474028
YPL253C	VIK1	-0.384759	-0.340417	-0.130678	-0.358575	-0.232158	0.39827
YML004C	GLO1	-0.152916	-0.060795	-0.130798	-0.448304	-0.186545	0.179841
YML058W-A	HUG1	-0.066317	0.059855	-0.131119	0.000651	-0.086107	-0.406747
YDL135C	RDI1	-0.316432	0.057649	-0.131436	-0.378748	-0.086945	0.094746
YPL273W	SAM4	0.385906	0.223886	-0.132107	0.389544	0.145653	-0.019786
YDL243C	AAD4	0.066864	-0.093708	-0.132527	-0.124394	-0.069283	0.922062
YOR052C	YOR052C	-0.239992	-0.154218	-0.133171	-0.25761	-0.071771	0.306441
YOR338W	YOR338W	-0.423645	-0.217015	-0.134313	-0.168451	-0.366929	-0.117271
YBR117C	TKL2	-0.005388	-0.025336	-0.13434	-0.459658	-0.382192	-0.110774
YML017W	PSP2	-0.027985	-0.091433	-0.134566	-0.007558	-0.02962	-0.303855
YGL204C	YGL204C	-0.095697	-0.139407	-0.13515	-0.040509	-0.282924	-0.332632
YNR007C	AUT1	-0.027877	-0.029558	-0.135663	-0.079202	-0.088675	0.392719
YCR102C	YCR102C	-0.097246	0.0366	-0.135868	0.077619	-0.032715	0.941601
YHR209W	YHR209W	-0.20853	-0.079169	-0.135915	0.010984	-0.36432	0.071126
YNR053C	YNR053C	0.214377	0.075508	-0.136833	0.082216	-0.368804	-0.68046
YLL020C	YLL020C	-0.183383	-0.039116	-0.137061	-0.246805	-0.088475	0.443631
YOR343C-A	YOR343C-A	-0.517231	-0.15464	-0.137786	0.082564	-0.242568	-0.150019
YDL223C	YDL223C	-0.251791	-0.103718	-0.140499	-0.645891	-0.483463	-0.004907
YBR101C	YBR101C	-0.187977	-0.153123	-0.140962	-0.339878	0.502751	0.469744
YHR140W	YHR140W	-0.217311	-0.132131	-0.141301	-0.238251	-0.569289	-0.236093
YHR056C	YHR056C	-0.012977	-0.121197	-0.142244	-0.512019	-0.354073	-0.136351
YPR052C	NHP6A	-0.083317	-0.159388	-0.142705	-0.022641	-0.111917	-0.394066
YER036C	YER036C	-0.141573	-0.028085	-0.142907	-0.20025	-0.064251	-0.512101
YML050W	YML050W	-0.310429	-0.192072	-0.144136	-0.183205	-0.095197	-0.152195
YPR110C	RPC40	0.321186	0.218577	-0.145382	0.194006	-0.138613	-0.082883
YOR003W	YSP3	-0.084314	-0.165041	-0.145972	0.086596	-0.33822	-0.136425
YJL045W	YJL045W	-0.106611	-0.215175	-0.147611	-0.401747	-0.621514	-0.055354
YNL167C	SKO1	-0.116603	-0.040621	-0.147631	-0.008999	0.187086	0.319867
YDL113C	YDL113C	-0.01238	-0.106637	-0.148576	-0.011608	0.029138	0.331163
YDR455C	YDR455C	0.051831	-0.04112	-0.149389	-0.120846	0.063493	0.395849
YJL034W	KAR2	-0.362718	-0.248934	-0.149411	-0.392077	0.438921	0.490954
YDR222W	YDR222W	0.104513	-0.099301	-0.153239	0.143793	0.32801	0.342331
YOL106W	YOL106W	0.117888	0.424607	-0.153332	-0.308725	-0.146499	-0.317983
YAL060W	YAL060W	-0.325754	-0.193035	-0.154969	-0.2954	-0.225948	-0.102761
YLR120C	YPS1	-0.40767	-0.098464	-0.155422	-0.125477	-0.032714	0.086183
YDR358W	GGA1	-0.038608	-0.076186	-0.156419	-0.309786	-0.037304	0.432322

YJL161W	YJL161W	-0.397281	-0.128613	-0.156781	-0.255064	-0.470137	0.009038
YJL171C	YJL171C	-0.398035	0.068623	-0.157575	0.146345	0.031505	0.504234
YGL101W	YGL101W	-0.119878	0.058425	-0.158021	0.203861	0.047006	-0.423647
YGR008C	STF2	-0.264326	-0.41739	-0.158617	0.030443	-0.178431	0.133869
YJR047C	ANB1	0.103683	0.124	-0.159687	-0.216299	-0.146002	-0.339564
YDR011W	SNQ2	-0.256499	-0.015175	-0.16109	-0.221616	0.108543	0.410699
YLR080W	YLR080W	-0.321238	-0.263871	-0.161175	-0.216196	-0.255461	0.12751
YMR135W-A	YMR135W-A	-0.014446	-0.013504	-0.161729	-0.055842	-0.387194	0.047811
YGR088W	CTT1	-0.018775	-0.103699	-0.161882	-0.621483	-0.21277	0.830516
YDL022W	GPD1	-0.132983	-0.297959	-0.162534	-0.073959	-0.154484	0.316637
YLR354C	TAL1	-0.023161	-0.077166	-0.162805	-0.306196	-0.189957	-0.361534
YOR092W	ECM3	-0.313412	-0.308962	-0.162988	-0.259653	-0.251738	0.3247
YBR093C	PHO5	0.040811	-0.065837	-0.163355	-0.123209	-0.056029	-0.638936
YGL006W	PMC1	-0.351326	-0.173164	-0.163651	-0.145916	-0.073081	-0.182491
YPR074C	TKL1	0.075944	0.17834	-0.163761	0.232372	-0.17328	-0.307919
YCR021C	HSP30	-0.582403	-0.1338	-0.164094	-0.803813	0.417494	0.756145
YBR045C	GIP1	-0.414221	-0.347639	-0.164121	-0.266307	0.076372	-0.130775
YFL056C	AAD6	0.081083	-0.085643	-0.164338	-0.238517	-0.005802	0.76413
YMR321C	YMR321C	0.272555	0.102462	-0.165005	0.388382	0.143487	-0.003218
YMR181C	YMR181C	-0.32529	-0.209291	-0.167366	-0.217282	-0.174148	0.227414
YLR300W	EXG1	0.00275	-0.134523	-0.16942	0.10626	-0.274417	-0.429244
YLL018C-A	COX19	-0.339287	-0.273432	-0.169756	-0.260752	-0.163189	0.279649
YHL021C	YHL021C	-0.338618	-0.220596	-0.170368	-0.564442	-0.145775	0.659328
YGR161W-A	YGR161W-A	-0.358161	-0.263292	-0.170432	-0.101476	-0.300249	-0.217245
YMR040W	YMR040W	-0.407666	-0.152522	-0.172173	-0.153072	-0.131046	-0.035655
YDR043C	NRG1	-0.307238	-0.079048	-0.173686	-0.091572	0.174341	0.496865
YMR164C	MSS11	-0.394463	-0.346755	-0.175821	-0.27939	-0.211998	0.421642
YBR132C	AGP2	-0.152425	-0.027322	-0.177823	-0.144924	-0.092909	0.361046
YPL166W	YPL166W	-0.167864	-0.018028	-0.180375	-0.122385	-0.073477	0.404396
YPL202C	YPL202C	-0.060417	-0.063685	-0.182426	-0.06333	-0.036421	0.336933
YGR130C	YGR130C	-0.148636	-0.106309	-0.182969	-0.324194	-0.05245	0.148172
YGL180W	APG1	-0.086495	-0.068134	-0.183221	-0.366468	-0.018169	0.602517
YBL064C	YBL064C	-0.028745	-0.367763	-0.184163	-0.189188	-0.105351	0.31331
YOR192C-A	YOR192C-A	-0.412111	-0.264234	-0.184483	-0.115458	-0.286694	-0.173038
YMR250W	GAD1	-0.14221	-0.145388	-0.184595	-0.3727	-0.14356	0.554368
YGR250C	YGR250C	-0.436691	-0.138064	-0.185098	-0.21501	0.194737	0.2843
YDL134C	PPH21	-0.099432	-0.322257	-0.185162	-0.115832	-0.097158	0.192538
YBL048W	YBL048W	-0.099834	-0.129885	-0.186111	-0.386362	0.008115	0.115694
YEL073C	YEL073C	-0.409168	-0.148831	-0.186674	-0.08093	-0.30248	0.03106
YOR107W	RGS2	-0.395367	-0.341704	-0.186824	-0.274157	-0.205669	0.264728
YJR046W	TAH11	-0.078854	-0.095322	-0.187346	-0.03733	0.437603	0.398151
YCR005C	CIT2	-0.225061	-0.173729	-0.1879	-0.209402	0.09836	0.363926
YNL007C	SIS1	-0.345717	-0.104763	-0.188599	-0.031235	0.225421	0.43208
Q0182	Q0182	0.355523	-0.173022	-0.190398	-0.317234	0.010856	-0.137481
YBL016W	FUS3	-0.077511	-0.135387	-0.190918	-0.210201	-0.139478	-0.323836
YOR146W	YOR146W	-0.159131	-0.168948	-0.191442	-0.336088	-0.016442	-0.211434
YDR074W	TPS2	-0.415833	-0.245939	-0.191852	-0.294355	-0.205425	0.485959
YJL016W	YJL016W	-0.455759	-0.306381	-0.192378	-0.229966	-0.363135	-0.040442
YPL282C	YPL282C	-0.340053	-0.227585	-0.192433	-0.111587	-0.196506	0.030613
YIL117C	PRM5	-0.081576	-0.10364	-0.192806	0.072355	-0.066032	0.315221
YAL044C	GCV3	0.031452	-0.220109	-0.193484	0.042201	-0.481789	-0.479454
YMR118C	YMR118C	-0.271001	-0.133412	-0.194664	-0.595576	-0.121991	0.052777

YJL142C	YJL142C	-0.218515	-0.091338	-0.196207	-0.144729	0.017726	0.363224
YFL002W-B	YFL002W-B	-0.380219	-0.281807	-0.19774	-0.098674	-0.266947	-0.154861
YOL104C	NDJ1	-0.212797	-0.173698	-0.198269	-0.077744	-0.359657	-0.148923
YDR487C	RIB3	0.145909	0.086581	-0.198987	0.201228	0.014955	0.328188
YJL145W	YJL145W	-0.37946	-0.336439	-0.199371	-0.200349	-0.20828	0.254461
YGR282C	BGL2	-0.002785	0.12913	-0.199484	-0.008008	-0.341386	-0.269354
YNL194C	YNL194C	-0.422918	-0.016864	-0.200867	-0.389378	-0.399933	0.124852
YNR064C	YNR064C	-0.434315	0.091524	-0.201121	-0.258504	-0.164935	-0.090618
YDR019C	GCV1	-0.164033	-0.357723	-0.201476	-0.183275	-0.424082	-0.21798
YDR525W-A	YDR525W-A	-0.357261	0.105376	-0.203434	-0.239557	-0.253507	0.071251
YHR208W	BAT1	-0.024054	-0.022247	-0.205212	0.051929	-0.21347	-0.377654
YER054C	GIP2	-0.301216	-0.111874	-0.205562	-0.133805	-0.164694	-0.014509
YCR052W	RSC6	-0.33482	-0.264664	-0.205841	-0.22681	-0.288925	0.313613
YPR172W	YPR172W	-0.185463	-0.109299	-0.2059	-0.15597	-0.47321	-0.06775
YKL209C	STE6	0.065619	0.039466	-0.207998	0.094411	-0.305625	-0.192178
YMR169C	ALD3	-0.30896	-0.161439	-0.208098	-0.856723	-0.676072	0.487035
YBL098W	YBL098W	0.067964	-0.163043	-0.208641	-0.289411	-0.306372	-0.157145
YDR119W	YDR119W	-0.136581	-0.069954	-0.210141	0.048359	-0.222359	-0.345013
YAR015W	ADE1	0.305424	-0.106029	-0.210606	-0.045985	-0.358026	-0.047729
YGR201C	YGR201C	-0.087342	-0.264193	-0.210657	-0.336411	-0.212431	-0.30037
YLR410W-A	YLR410W-A	-0.312671	-0.197053	-0.211518	-0.058854	-0.225286	-0.154202
YOR304W	ISW2	-0.271882	-0.221799	-0.216244	-0.093961	-0.184793	0.359766
YCL040W	GLK1	-0.218404	-0.428504	-0.216486	-0.524276	-0.341239	0.274326
YLL055W	YLL055W	-0.073705	0.073352	-0.219102	0.161322	0.133996	0.74871
YDR258C	HSP78	-0.274325	-0.130725	-0.219253	-0.109006	0.319731	0.199252
YGR213C	RTA1	-0.323296	-0.114973	-0.219273	0.123513	-0.164604	0.174859
YIL167W	YIL167W	0.081889	-0.064219	-0.222616	-0.052184	-0.000417	0.429752
YLR004C	YLR004C	0.149277	-0.101531	-0.222703	-0.446621	-0.163507	-0.057588
YPL135W	ISU1	-0.107601	-0.025033	-0.223067	-0.124781	-0.100956	0.453473
YOR298C-A	MBF1	-0.388805	-0.146068	-0.22831	-0.179415	0.099188	0.078047
YDL048C	STP4	-0.323387	-0.187699	-0.229426	-0.43914	-0.328003	0.016056
YJR096W	YJR096W	-0.244026	-0.26872	-0.229997	-0.530079	-0.335979	0.261754
YBL101W-A	YBL101W-A	-0.345616	-0.171782	-0.232104	-0.126797	-0.238094	-0.158628
YDR261W-A	YDR261W-A	-0.474951	-0.286476	-0.232646	-0.080565	-0.310347	-0.190983
YKL182W	FAS1	0.071611	-0.151983	-0.232738	-0.060264	-0.103175	-0.332619
YBR105C	VID24	0.011029	-0.080954	-0.233441	-0.32497	-0.288036	-0.2054
YER067W	YER067W	-0.69026	-0.342719	-0.233571	-0.206956	-0.598912	0.112365
YKL111C	YKL111C	-0.421477	-0.094312	-0.235484	-0.040051	0.043814	0.05355
YHR097C	YHR097C	-0.223903	-0.411315	-0.235585	-0.319721	-0.243166	0.108505
YOR273C	YOR273C	-0.251153	-0.075422	-0.238801	-0.034085	-0.026329	0.682149
YAR071W	PHO11	-0.109929	-0.072034	-0.241542	-0.064807	-0.220762	-0.758032
YPL240C	HSP82	-0.062779	-0.025161	-0.242056	-0.132928	0.437132	0.131788
YPR158W	YPR158W	-0.34721	-0.074801	-0.242836	-0.049532	0.261513	0.361946
YOL058W	ARG1	-0.152149	0.109983	-0.243036	0.027437	-0.090439	0.600674
YDR171W	HSP42	-0.210359	-0.340348	-0.243625	-0.247884	0.383414	0.532036
YDL043C	PRP11	0.063355	0.308254	-0.246548	-0.014236	-0.053853	-0.139072
YIL053W	RHR2	-0.09189	-0.17044	-0.247152	-0.187199	-0.360417	-0.505963
YLR297W	YLR297W	-0.072099	-0.083593	-0.248559	-0.205818	-0.299185	0.317894
YIL157C	YIL157C	-0.297192	-0.262445	-0.2505	-0.278262	-0.224945	0.409432
YOL151W	GRE2	-0.304179	-0.125189	-0.251292	-0.225015	-0.268467	0.867997
YOL119C	YOL119C	-0.216662	0.034037	-0.251503	-0.118669	-0.131775	0.301582
YKL035W	UGP1	-0.250016	-0.211521	-0.252465	-0.329852	0.025129	0.310767

YPR201W	ARR3	-0.073613	-0.116171	-0.255663	0.077146	-0.213931	0.387633
YJL026W	RNR2	-0.125297	-0.25959	-0.256014	-0.284121	-0.343702	-0.167611
Q0110	BI2	0.058756	-0.151477	-0.25628	-0.35009	0.091022	-0.109121
YNR034W	SOL1	-0.306711	-0.200749	-0.256831	-0.240131	0.112329	0.331927
YGR260W	TNA1	-0.239508	-0.080631	-0.257245	-0.248953	-0.43568	-0.179839
YOR028C	CIN5	0.059719	0.137123	-0.258058	-0.11564	-0.040944	0.621142
YMR176W	ECM5	0.028951	-0.043946	-0.259352	0.024795	-0.138535	-0.320935
YOR027W	STI1	-0.224327	-0.193791	-0.259559	-0.250638	0.132995	0.319778
YLR251W	YLR251W	-0.252896	-0.17349	-0.260271	0.001301	0.002531	0.437782
YDR034C-C	YDR034C-C	-0.371881	-0.23325	-0.261569	-0.14042	-0.371119	-0.234406
YMR189W	GCV2	-0.058942	-0.08024	-0.262679	-0.117689	-0.417705	-0.259651
YHR202W	YHR202W	0.019455	0.046222	-0.262852	-0.057967	-0.214982	-0.317737
YBR162C	TOS1	0.059356	0.073854	-0.263403	-0.023552	-0.012876	-0.31347
YGR142W	BTN2	-0.241517	-0.247998	-0.267647	-0.644855	0.641218	0.308358
YJL017W	YJL017W	-0.489232	-0.319833	-0.268297	-0.248117	-0.319072	-0.016858
YNL327W	EGT2	-0.187504	-0.161663	-0.270243	-0.383997	-0.216559	-0.346877
YLR097C	YLR097C	-0.354565	-0.245455	-0.27051	-0.261753	-0.265199	0.289153
YHR139C	SPS100	-0.467029	-0.04547	-0.271639	-0.911166	-0.079811	-0.112025
YHR215W	PHO12	-0.162832	-0.121358	-0.280131	-0.073803	-0.215999	-0.692945
YGR279C	SCW4	0.214622	0.033898	-0.280258	0.111118	-0.033116	-0.422454
YDL124W	YDL124W	-0.39102	-0.305142	-0.283977	-0.318773	-0.316995	0.458316
YFL058W	THI5	-0.124885	-0.328966	-0.285595	-0.150197	-0.157176	0.052915
YHR186C	YHR186C	-0.374713	-0.337566	-0.286146	-0.354752	-0.23797	0.393718
YLL024C	SSA2	-0.304626	-0.33877	-0.287017	-0.102532	0.238429	0.085912
YJL144W	YJL144W	-0.26797	-0.415892	-0.287598	-0.319162	0.353716	0.521524
YDL049C	KNH1	-0.40244	-0.436132	-0.287995	-0.319586	-0.326079	0.305312
YDR233C	YDR233C	-0.060995	-0.118575	-0.288764	-0.123101	-0.361854	-0.318059
YNL078W	YNL078W	0.197708	0.020903	-0.288954	-0.078393	-0.145604	-0.301195
YLR178C	TFS1	-0.176967	-0.387826	-0.288971	-0.187084	-0.414666	-0.011906
YOL158C	ENB1	-0.390981	0.090968	-0.290907	0.094525	0.084912	-0.238975
YJL089W	SIP4	-0.477106	-0.071762	-0.292783	-0.215429	0.260088	0.189159
YDL244W	THI13	-0.174243	-0.483495	-0.293967	-0.218055	-0.357457	-0.199519
YCL020W	YCL020W	-0.262401	-0.18583	-0.298274	-0.057702	-0.363028	-0.264157
YMR130W	YMR130W	-0.006696	0.062336	-0.302986	-0.158799	0.092648	0.124319
YLR258W	GSY2	-0.061442	-0.101066	-0.302993	-0.345747	-0.045218	0.360561
YOR202W	HIS3	0.174595	0.165178	-0.304291	0.307244	0.034653	-0.094806
YLR042C	YLR042C	-0.134062	-0.08743	-0.30514	-0.162421	-0.340422	-0.055685
YMR105C	PGM2	-0.355104	-0.400031	-0.305648	-0.343023	-0.549025	0.064599
YAL005C	SSA1	-0.300157	-0.412878	-0.307057	-0.212783	0.247822	0.133003
YLR164W	YLR164W	-0.118669	-0.080768	-0.307233	-0.336265	-0.026691	0.416017
YDR421W	YDR421W	0.001518	-0.056278	-0.308134	-0.183604	0.001817	0.263304
YLR152C	YLR152C	-0.248441	-0.079763	-0.30875	-0.121826	-0.097517	-0.253596
YCL055W	KAR4	0.083639	-0.011521	-0.311346	-0.003665	0.001812	-0.241581
YMR103C	YMR103C	-0.095117	-0.050796	-0.311395	0.056305	-0.291424	0.059633
YDL167C	NRP1	0.114409	0.010093	-0.311733	-0.181086	-0.20522	0.353694
YGR071C	YGR071C	0.073348	0.008856	-0.312614	-0.092859	-0.027548	-0.022439
YER061C	CEM1	-0.029881	-0.129242	-0.313681	0.056435	-0.305071	-0.038406
YPL209C	IPL1	-0.077548	-0.061807	-0.314988	0.136169	0.059593	-0.131605
YGR240C	PFK1	-0.215269	-0.153517	-0.316188	-0.088979	-0.152753	-0.192664
YLL026W	HSP104	-0.41055	-0.293414	-0.316404	-0.355957	0.34919	0.747327
YDR070C	YDR070C	-0.393578	-0.306171	-0.317402	-0.300882	-0.307357	0.090248
YHR015W	MIP6	-0.10253	-0.240306	-0.317785	-0.105991	-0.366499	-0.164405

YKR042W	UTH1	-0.04914	0.004715	-0.317931	0.015624	0.00531	0.004509
YOR011W	AUS1	-0.019231	0.046239	-0.317988	-0.03772	-0.035224	-0.070528
YNL334C	SNO2	-0.153383	-0.224614	-0.318192	-0.131708	-0.245176	-0.22615
YDR403W	DIT1	-0.327699	-0.130029	-0.318735	0.275274	-0.183098	-0.16778
YPL092W	SSU1	0.042346	-0.028771	-0.319207	-0.02054	-0.322977	-0.130262
YMR170C	ALD2	0.003476	-0.104312	-0.322337	-0.221988	-0.193367	-0.075635
YHR041C	SRB2	-0.153603	-0.203784	-0.322464	-0.166376	-0.228112	-0.136631
YOR153W	PDR5	-0.254808	-0.171095	-0.322676	0.199149	-0.111529	-0.091031
YAL037W	YAL037W	-0.043243	-0.09938	-0.324096	0.018021	-0.076177	-0.155379
YLR001C	YLR001C	-0.032865	0.000177	-0.326049	-0.122821	-0.033423	0.214836
YER091C	MET6	-0.298619	-0.325945	-0.326589	-0.151097	-0.593581	-0.464551
YDR406W	PDR15	-0.313528	-0.157295	-0.327252	-0.034172	-0.144864	-0.165131
YLR355C	ILV5	-0.028277	-0.049683	-0.327573	-0.179671	-0.305331	-0.145137
YOL153C	YOL153C	-0.2783	-0.090927	-0.328901	-0.092981	-0.063413	0.548814
YHR084W	STE12	0.044353	0.023633	-0.329808	0.01479	-0.111992	-0.305086
YBR287W	YBR287W	-0.273882	-0.000291	-0.330537	-0.172694	-0.155278	0.153822
YKL163W	PIR3	-0.062392	-0.219633	-0.330708	-0.002683	-0.220147	-0.101821
YOL064C	MET22	-0.152958	-0.206054	-0.331633	-0.024881	-0.064082	-0.029623
YHR162W	YHR162W	-0.130285	-0.064157	-0.331778	-0.25166	-0.167653	-0.085086
YDR037W	KRS1	0.285691	0.163246	-0.332112	0.237527	0.000421	-0.007657
YNL296W	YNL296W	-0.137144	-0.082162	-0.332124	-0.042596	0.091598	0.02994
YER103W	SSA4	-0.350994	-0.174637	-0.332647	-0.599336	0.369939	0.56408
YPL264C	YPL264C	-0.050743	-0.04711	-0.332905	-0.130804	-0.043352	0.041107
YIR034C	LYS1	0.033242	0.068247	-0.333875	0.066492	-0.053353	-0.007886
YKL051W	YKL051W	-0.351718	0.110609	-0.335336	-0.166741	-0.212708	0.450369
YEL057C	YEL057C	-0.074518	-0.114065	-0.335578	-0.137496	-0.311021	-0.107924
YDR354W	TRP4	0.047454	0.001525	-0.335737	0.059614	0.01397	0.008647
YER187W	YER187W	-0.180997	-0.264825	-0.335767	-0.495239	-0.13895	-0.157202
YNL114C	YNL114C	0.061176	-0.036235	-0.336028	-0.302691	-0.221208	-0.367763
YGL146C	YGL146C	-0.1697	-0.153596	-0.337479	-0.040879	-0.15524	-0.003234
YDR474C	YDR474C	-0.130143	-0.108406	-0.338405	-0.069253	-0.137897	0.211772
YCR089W	FIG2	0.101372	-0.007185	-0.339749	-0.167092	0.085691	-0.03882
YGL028C	SCW11	-0.041993	-0.127998	-0.341019	-0.279733	-0.363135	-0.213063
YHR190W	ERG9	-0.308192	-0.53245	-0.341706	-0.357102	-0.282087	0.445226
YIL166C	YIL166C	-0.132187	-0.023246	-0.342002	0.048045	-0.014876	0.065337
YIL111W	COX5B	-0.239202	-0.236337	-0.342079	0.10233	-0.091255	-0.09951
YKL162C-A	YKL162C-A	-0.216512	-0.211247	-0.342965	-0.011695	-0.400902	-0.079213
YKR097W	PCK1	-0.170736	-0.055566	-0.347761	-0.236631	-0.215845	-0.108677
YLR252W	YLR252W	-0.274423	-0.165872	-0.348519	-0.006419	-0.052602	0.62214
YLR142W	PUT1	-0.141896	-0.371422	-0.348712	-0.164723	-0.070697	-0.035456
YIR017C	MET28	-0.448303	-0.333615	-0.349311	0.019297	-0.048899	0.131735
YFL026W	STE2	0.24811	-0.041218	-0.349603	-0.108592	-0.364916	-0.284739
YER062C	HOR2	-0.059808	-0.216265	-0.351101	-0.031183	-0.42149	-0.550943
YFL060C	SNO3	-0.139831	-0.241067	-0.351807	-0.179275	-0.24442	-0.162041
YBR115C	LYS2	0.108749	0.08626	-0.353166	0.078708	-0.008773	0.004976
YLR422W	YLR422W	0.162971	0.033634	-0.35365	-0.082009	0.066051	-0.11541
YDR085C	AFR1	-0.125892	-0.051127	-0.354698	-0.113649	-0.203321	0.18705
YOL134C	YOL134C	0.026941	-0.01157	-0.355253	-0.086534	-0.154757	-0.104433
YBR256C	RIB5	0.188965	0.045756	-0.359916	-0.026821	0.07918	0.109894
YHR021W-A	ECM12	-0.016798	0.174006	-0.361544	0.016164	-0.096912	-0.166485
YDL246C	YDL246C	-0.511332	-0.520922	-0.362333	-0.20796	-0.448239	-0.411882
YCR099C	YCR099C	-0.121289	-0.075075	-0.36415	-0.020243	0.128142	0.390498

YNL015W	PBI2	-0.379209	-0.55824	-0.365132	-0.175246	-0.630968	-0.188494
YER124C	YER124C	-0.161954	-0.148778	-0.365565	-0.493284	-0.464944	-0.286689
YMR271C	URA10	-0.114595	-0.076554	-0.366981	-0.361601	-0.292614	-0.001254
YHR005C	GPA1	-0.026249	-0.111647	-0.367169	0.019205	-0.127217	-0.38032
YGR086C	YGR086C	-0.319201	-0.064529	-0.368294	-0.227361	-0.19958	-0.282441
YIL015C-A	YIL015C-A	-0.059194	-0.175128	-0.369223	0.142162	-0.255433	-0.168266
YKL185W	ASH1	-0.053016	-0.146613	-0.369613	-0.094729	-0.219148	-0.173707
YGL259W	YPS5	-0.267324	-0.079997	-0.370448	0.024287	-0.280529	-0.060639
YPL280W	YPL280W	-0.199491	0.038906	-0.371297	-0.397741	-0.248769	-0.096894
YJL108C	PRM10	0.039034	-0.117309	-0.37149	0.221245	-0.139114	0.003646
YML116W	ATR1	0.001909	-0.068686	-0.371596	0.019176	-0.030474	0.23574
YNR069C	YNR069C	-0.238869	-0.078796	-0.371613	-0.149037	0.326835	0.620668
YER090W	TRP2	0.182356	0.087929	-0.371968	0.046761	0.027857	0.092583
YDL066W	IDP1	0.010065	0.052441	-0.371977	0.075815	-0.015345	0.064895
YBR169C	SSE2	-0.236724	-0.368235	-0.372113	-0.411585	0.26432	0.642616
YGL248W	PDE1	-0.392272	-0.186339	-0.373183	-0.110431	-0.272767	-0.061567
YIL082W	YIL082W	-0.014363	-0.013086	-0.375876	-0.080977	-0.033252	-0.226254
YKL211C	TRP3	0.195508	0.033491	-0.377116	0.002057	-0.026377	0.05676
YOR382W	YOR382W	-0.088779	-0.091742	-0.377768	-0.123992	-0.114542	-0.264971
YHR059W	YHR059W	0.049894	0.177399	-0.380198	0.175767	-0.009079	-0.04827
YGL202W	ARO8	0.137329	0.121704	-0.380362	0.085711	0.084388	0.056684
YMR322C	YMR322C	-0.328134	-0.164817	-0.381446	-0.357097	-0.365175	-0.063709
YOR391C	YOR391C	-0.278961	0.009263	-0.383112	-0.368992	-0.380792	-0.049574
YGL117W	YGL117W	0.031021	-0.128117	-0.384817	0.014144	-0.047251	0.497675
YIL082W-A	YIL082W-A	-0.03429	-0.143143	-0.390803	-0.176635	0.010986	-0.137677
YJL157C	FAR1	-0.01378	-0.027973	-0.394371	-0.114447	-0.276226	-0.387717
YDR127W	ARO1	0.131913	-0.000183	-0.394564	0.040476	-0.007309	-0.045563
YKL103C	LAP4	-0.072869	-0.063893	-0.397741	-0.127638	-0.153723	0.656381
YIL009C-A	EST3	-0.332909	-0.037725	-0.398817	-0.050193	-0.381641	-0.414811
YER069W	ARG5	0.063153	-0.090796	-0.399564	0.106734	-0.017226	0.033334
YIL080W	YIL080W	0.00716	-0.134108	-0.40194	-0.128792	-0.032746	-0.166034
YPR005C	HAL1	-0.132868	-0.18983	-0.402921	-0.090596	-0.109331	-0.05513
YGL008C	PMA1	-0.176382	-0.122782	-0.403357	0.022996	-0.115955	-0.095726
YGR109W-B	YGR109W-B	-0.026935	-0.113643	-0.40518	-0.131867	-0.05679	-0.210112
YKL184W	SPE1	0.010787	-0.036547	-0.408222	0.002546	0.049503	0.065984
YDR195W	REF2	-0.007	-0.067075	-0.40965	-0.197215	-0.014136	-0.193327
YER055C	HIS1	0.077865	0.021662	-0.40977	0.113586	-0.268331	-0.06615
YPL058C	PDR12	-0.123662	0.169396	-0.410013	-0.119906	0.191701	0.121278
YIR042C	YIR042C	-0.244592	-0.127582	-0.413973	-0.032233	-0.052715	-0.11012
YGL251C	HFM1	-0.288022	-0.217172	-0.415395	-0.138244	-0.430903	-0.237151
YML047C	PRM6	-0.065884	-0.020044	-0.416169	-0.043192	-0.016632	0.029661
YBR183W	YPC1	-0.473272	-0.32836	-0.41755	-0.338442	-0.347454	-0.06713
YDR022C	CIS1	-0.463181	-0.337959	-0.419312	-0.114446	-0.584797	-0.292732
YIL015W	BAR1	0.046762	-0.044578	-0.419639	-0.3656	-0.474581	-0.494514
YNR006W	VPS27	-0.123013	-0.048192	-0.419809	-0.075123	0.042611	0.122956
YGL249W	ZIP2	-0.246437	-0.246466	-0.420382	-0.308484	-0.281266	0.344033
YML128C	YML128C	-0.211598	-0.15322	-0.421026	-0.434441	-0.361227	0.287129
YLR286C	CTS1	-0.008596	-0.018364	-0.423777	-0.299094	-0.214243	-0.16322
YDR380W	YDR380W	0.022886	-0.345295	-0.424258	-0.330583	-0.333842	-0.208017
YLL057C	YLL057C	-0.400054	-0.205167	-0.429655	-0.266072	-0.193319	-0.052141
YLR040C	YLR040C	-0.15452	-0.182244	-0.42998	-0.272008	-0.265104	-0.210102
YDR158W	HOM2	0.076061	0.064938	-0.43141	-0.008418	0.072558	-0.043778

YHR071W	PCL5	0.068873	0.055245	-0.434594	-0.167947	-0.050805	0.030874
YGR045C	YGR045C	-0.364765	-0.32358	-0.435918	-0.315163	-0.458077	0.129496
YGR146C	YGR146C	-0.150977	-0.239115	-0.438244	-0.312346	0.056827	-0.013062
YML100W	TSL1	-0.430231	-0.390605	-0.439893	-0.242103	-0.183982	0.628394
YGR248W	SOL4	-0.612244	-0.3689	-0.441817	-0.417243	-0.525409	0.175043
YPL036W	PMA2	-0.056877	-0.148307	-0.450778	-0.212377	-0.140688	-0.241433
YLR327C	YLR327C	-0.25134	-0.590651	-0.452206	-0.091003	0.073418	-0.162711
YDR112W	YDR112W	0.14072	0.042206	-0.452495	-0.251885	0.046886	-0.190206
YML060W	OGG1	-0.160697	0.002449	-0.456418	-0.170603	0.086306	-0.018154
YOR344C	TYE7	-0.411351	-0.221971	-0.460682	-0.212176	-0.200955	0.001182
YDR242W	AMD2	-0.054366	-0.111981	-0.461206	0.045877	-0.165465	-0.162958
YOR130C	ORT1	-0.109045	-0.101208	-0.461525	0.144563	-0.046021	0.005196
YGR044C	RME1	-0.32186	-0.327993	-0.463962	-0.294021	-0.514598	0.085046
YHL044W	YHL044W	-0.37612	-0.224095	-0.465728	0.014653	-0.196126	-0.141277
YHL012W	YHL012W	-0.168075	-0.103253	-0.468325	-0.10008	-0.347465	-0.193927
YHR143W	YHR143W	-0.121168	-0.030158	-0.468622	-0.299295	-0.352695	-0.175749
YOR173W	YOR173W	-0.441347	-0.350681	-0.468968	-0.304215	-0.519023	0.132749
YER096W	SHC1	-0.176399	-0.272197	-0.472529	-0.260307	0.111601	0.04593
YHR136C	SPL2	-0.433731	-0.289123	-0.473752	-0.195542	-1.009958	-1.024551
YMR062C	ECM40	-0.074926	0.009106	-0.473956	0.131954	-0.02795	0.040087
YDL024C	DIA3	-0.367467	-0.300903	-0.474197	-0.212908	-0.398915	-0.467243
YDR276C	PMP3	-0.489458	-0.237529	-0.475874	-0.28679	-0.321743	-0.008226
YLR058C	SHM2	-0.002988	-0.160074	-0.476756	-0.116126	-0.594375	-0.131242
YHR018C	ARG4	0.053107	-0.033558	-0.477498	0.005806	-0.167719	0.069807
YLR452C	SST2	0.14045	0.121833	-0.477844	-0.003379	-0.037924	-0.302416
YLR231C	YLR231C	-0.286332	-0.256444	-0.479202	-0.317682	-0.441244	-0.412806
YBR019C	GAL10	-0.360165	-0.10466	-0.479889	-0.251095	-0.355139	-0.210156
YKL198C	PTK1	-0.255789	-0.140398	-0.484809	-0.1964	-0.075489	-0.071857
YDR035W	ARO3	0.005948	0.01164	-0.485976	-0.008716	-0.143135	0.019518
YHR137W	ARO9	0.004883	-0.137055	-0.486721	0.025631	-0.059519	-0.379877
YER150W	SPI1	-0.397976	-0.308153	-0.488071	-0.738257	0.322181	0.391958
YBR047W	YBR047W	-0.214008	-0.167369	-0.490378	-0.140062	-0.238936	-0.032716
YOR161C	YOR161C	-0.417721	-0.238089	-0.491724	-0.518871	-0.453435	0.122734
YHR055C	CUP1-2	-0.227475	-0.360661	-0.492158	-0.278157	0.157166	0.452882
YOL052C-A	DDR2	-0.349228	-0.430802	-0.496948	-0.326215	-0.430296	0.772152
YPR149W	NCE102	-0.390259	-0.291804	-0.498487	-0.297902	-0.171908	0.238696
YOR343C	YOR343C	0.05284	-0.18717	-0.503973	0.091651	0.049356	0.047648
YNL145W	MFA2	-0.208425	-0.292367	-0.509493	-0.197688	-0.525724	-0.643504
YDL021W	GPM2	-0.441104	-0.463048	-0.515086	-0.075118	-0.446783	-0.171504
YDR461W	MFA1	-0.064924	-0.227233	-0.519327	-0.072124	-0.315787	-0.498101
YHR053C	CUP1-1	-0.205954	-0.332633	-0.51944	-0.27609	0.168187	0.456099
YDR533C	YDR533C	-0.464288	-0.204497	-0.524845	-0.33156	-0.874766	0.319947
YNR068C	YNR068C	-0.165886	-0.019492	-0.531243	-0.129399	0.256104	0.655414
YHR087W	YHR087W	-0.348119	-0.691067	-0.543689	-0.411702	-0.229984	0.468616
YDR539W	YDR539W	-0.288575	-0.353027	-0.545101	-0.188679	-0.305448	-0.440107
YPL017C	YPL017C	-0.428933	-0.377405	-0.554159	-0.246075	-0.407137	-0.275578
YIL116W	HIS5	0.057058	-0.057406	-0.559924	0.014176	-0.198888	-0.025787
YMR173W	DDR48	-0.440742	-0.559069	-0.565994	-0.419766	-0.323508	0.569132
YPL088W	YPL088W	-0.013597	-0.122999	-0.566916	-0.096766	-0.185314	-0.22398
YIR030C	DCG1	-0.57233	-0.08335	-0.571511	-0.082281	-0.803485	-0.289312
YDR034W-B	YDR034W-B	-0.493221	-0.545885	-0.571688	-0.426321	-0.792318	-0.087783
YER073W	ALD5	-0.026473	-0.103085	-0.575212	-0.036791	-0.05075	-0.09043

YMR095C	SNO1	0.025373	-0.021679	-0.578372	-0.04892	-0.270909	-0.172633
YJL172W	CPS1	-0.25952	-0.06156	-0.581988	0.005191	-0.191956	-0.11415
YJR109C	CPA2	0.090954	0.110829	-0.600245	0.050148	-0.121489	0.334529
YER175C	YER175C	0.066233	0.003958	-0.604561	-0.025318	0.062376	0.172972
YOR302W	YOR302W	0.039944	-0.075595	-0.609478	-0.275196	-0.094982	0.072081
YPL250C	ICY2	-0.349918	-0.161924	-0.615706	-0.382197	0.362835	0.051354
YJL170C	ASG7	-0.278531	-0.186615	-0.61595	-0.279918	-0.636541	-0.599009
YER052C	HOM3	-0.154944	-0.042391	-0.627622	-0.132266	0.054037	0.247169
YGL121C	YGL121C	-0.158572	-0.439188	-0.630517	-0.012122	-0.589477	-0.080165
YKL218C	SRY1	-0.49615	-0.179288	-0.639946	-0.130482	-0.301836	-0.112752
YML123C	PHO84	-0.087609	-0.172343	-0.647639	-0.347678	-0.78942	-0.763794
YCL030C	HIS4	0.16555	-0.076793	-0.649936	0.181109	-0.253446	0.11029
YIL164C	NIT1	-0.078958	-0.02393	-0.653088	0.037443	0.09519	0.56166
YPR160W	GPH1	-0.293488	-0.11392	-0.654157	-0.268346	-0.355476	-0.091212
YDR453C	YDR453C	-0.069176	-0.167881	-0.664586	-0.292147	-0.481388	0.503458
YIL165C	YIL165C	0.09385	-0.047744	-0.697728	0.193971	-0.073097	0.385659
YKL096W	CWP1	-0.150952	-0.00003	-0.71544	-0.028098	-0.369663	-0.201173
YOR303W	CPA1	0.107332	-0.137549	-0.721809	-0.241027	-0.097561	0.094167
YCL027W	FUS1	-0.355191	-0.172295	-0.726476	-0.304313	-0.371846	-0.205628
YHR029C	YHR029C	-0.179147	-0.233888	-0.744948	0.025696	-0.227156	0.105937
YBR072W	HSP26	-0.629551	-0.891273	-0.757502	-0.384263	0.04902	0.356344
YBR285W	YBR285W	-0.55984	-0.124832	-0.757813	-0.045646	-0.701535	-0.070204
YDR033W	MRH1	-0.448773	-0.365004	-0.760212	0.004482	-0.793385	-0.050532
YGL032C	AGA2	-0.009256	-0.071115	-0.769891	-0.173618	-0.188654	-0.60702
YJR025C	BNA1	-0.305385	-0.349079	-0.794256	-0.325601	-0.393393	-0.3698
YNR044W	AGA1	0.040003	-0.063132	-0.823156	-0.198125	-0.025684	-0.389051
YHR022C	YHR022C	-0.351966	-0.208803	-0.856121	-0.186469	-0.817411	-0.155902
YMR096W	SNZ1	-0.044855	0.040403	-0.943327	-0.273394	-0.306055	0.04024
YNL160W	YGP1	-0.408187	-0.378324	-0.95492	-0.456471	-0.318443	0.156798
YBR054W	YRO2	-0.79197	-0.376038	-0.99495	-0.382902	-0.270761	0.708104
YOR255W	YOR255W	0.155214	0.099769	-1.053917	-0.20161	0.022408	0.017884
YFL014W	HSP12	-0.338492	-0.857479	-1.064175	-0.363242	-0.296316	-0.002745
