# Supplementary material for: Genetic Interactions of MAF1 Identify a Role for Med20 in Transcriptional Repression of Ribosomal Protein Genes
Source: PLoS Genet. 2008 Jul 4;4(7):e1000112. doi: 10.1371/journal.pgen.1000112 (PMC2435279; doi:10.1371/journal.pgen.1000112)
Supplement: Table S5 — Expression ratios of ribosomal protein genes comparing different Mediator subunit deletions versus wild-type after rapamycin treatment. (0.04 MB PDF) [file pgen.1000112.s009.pdf]

Table S5
Expression ratios of ribosomal protein genes comparing different Mediator subunit deletions versus wild-type after rapamycin treatment[a]

UNIQID	Gene Name	med16/WT + Rap	cycC/WT + Rap	med31/WT + Rap
YOR063W	RPL3	2.55	1.13	2.53
YOR096W	RPS7A	1.95	1.19	1.07
YHR203C	RPS4B	1.94	1.06	1.14
YJR145C	RPS4A	1.91	1.13	1.14
YML024W	RPS17A	1.87	1.11	0.90
YJL189W	RPL39	1.85	0.47	0.77
YNL178W	RPS3	1.83	1.36	0.84
YOL127W	RPL25	1.83	1.30	0.96
YHR021C	RPS27B	1.80	1.21	0.83
YDR382W	RPP2B	1.78	1.09	1.40
YNL067W	RPL9B	1.77	0.78	1.17
YJL190C	RPS22A	1.77	1.04	1.54
YOL121C	RPS19A	1.72	0.83	1.10
YMR142C	RPL13B	1.70	1.12	1.24
YMR230W	RPS10B	1.69	1.21	2.57
YJR094W-	RPL43B	1.68	1.12	0.98
YPL081W	RPS9A	1.67	0.90	1.54
YPL079W	RPL21B	1.67	0.97	1.05
YDR418W	RPL12B	1.66	0.91	1.05
YGR034W	RPL26B	1.66	1.03	0.92
YHR141C	RPL42B	1.64	0.90	0.76
YGL103W	RPL28	1.64	1.21	1.16
YLL045C	RPL8B	1.64	1.16	1.21
YBR191W	RPL21A	1.63	0.95	1.03
YLR448W	RPL6B	1.63	1.08	1.08
YML073C	RPL6A	1.63	1.01	1.03
YLR185W	RPL37A	1.63	1.03	0.82
YDR500C	RPL37B	1.62	1.08	0.91
YPL198W	RPL7B	1.62	0.97	1.23
YOR312C	RPL20B	1.62	0.95	1.01
YNL302C	RPS19B	1.62	1.09	1.09
YGL147C	RPL9A	1.62	0.86	1.23
YLR029C	RPL15A	1.61	0.98	1.31
YNL069C	RPL16B	1.61	0.86	0.91
YNL162W	RPL42A	1.60	1.04	0.75
YEL054C	RPL12A	1.60	0.99	1.06
YGL030W	RPL30	1.60	1.06	1.14
YDR471W	RPL27B	1.57	0.88	0.83
YER074W	RPS24A	1.56	1.04	0.95
YIL018W	RPL2B	1.56	0.86	1.04
YLR441C	RPS1A	1.56	0.83	1.39
YGL076C	RPL7A	1.56	0.94	1.27
YOR167C	RPS28A	1.54	0.94	1.09
YLR287C-/	RPS30A	1.54	1.08	0.84
YOR369C	RPS12	1.54	1.15	1.80
YMR242C	RPL20A	1.54	1.04	0.98

YHL033C	RPL8A	1.53	1.13	1.29
YLR075W	RPL10	1.53	1.38	1.66
YER117W	RPL23B	1.52	0.97	0.96
YDL061C	RPS29B	1.51	1.02	0.91
YLR264W	RPS28B	1.51	1.13	1.21
YOR234C	RPL33B	1.51	0.94	0.81
YDL083C	RPS16B	1.49	0.80	1.10
YLR388W	RPS29A	1.49	1.04	0.85
YPL131W	RPL5	1.48	0.91	1.73
YDR450W	RPS18A	1.48	1.00	1.06
YML026C	RPS18B	1.48	1.08	1.04
YKR057W	RPS21A	1.48	0.76	1.09
YER131W	RPS26B	1.48	0.97	0.88
YDR064W	RPS13	1.47	1.03	1.46
YGR085C	RPL11B	1.47	1.13	0.83
YGR027C	RPS25A	1.47	1.00	1.12
YJR123W	RPS5	1.46	0.73	0.88
YBR189W	RPS9B	1.46	0.94	1.34
YFR031C-/	RPL2A	1.45	0.86	1.04
YLR167W	RPS31	1.45	1.04	0.95
YGL189C	RPS26A	1.44	0.96	0.88
YBL087C	RPL23A	1.43	1.02	1.02
YJL177W	RPL17B	1.42	1.22	0.96
YIL148W	RPL40A	1.41	0.86	0.83
YGL123W	RPS2	1.41	0.83	1.37
YBL072C	RPS8A	1.39	1.04	1.22
YHR010W	RPL27A	1.37	1.06	0.86
YOR293W	RPS10A	1.37	0.97	0.90
YKL180W	RPL17A	1.36	1.11	0.99
YFL034C-/	RPL22B	1.36	0.67	0.91
YCR031C	RPS14A	1.36	1.10	1.11
YGR214W	RPS0A	1.35	0.83	1.05
YLR344W	RPL26A	1.35	0.98	0.94
YPL249C-/	RPL36B	1.33	1.04	0.95
YDL082W	RPL13A	1.33	1.12	1.25
YNL096C	RPS7B	1.32	1.03	0.98
YPL143W	RPL33A	1.32	0.91	0.79
YDL081C	RPP1A	1.32	1.04	1.36
YPR102C	RPL11A	1.30	0.61	0.98
YLR048W	RPS0B	1.30	0.99	1.38
YMR143W	RPS16A	1.30	0.89	0.94
YLR406C	RPL31B	1.30	1.42	0.87
YDL184C	RPL41A	1.30	1.28	0.95
YPL090C	RPS6A	1.29	1.06	1.07
YHL001W	RPL14B	1.29	1.08	1.12
YJL191W	RPS14B	1.28	0.94	1.07
YKL156W	RPS27A	1.28	1.03	0.85
YLR340W	RPP0	1.28	1.14	1.27
YGL135W	RPL1B	1.28	0.92	1.09
YBL027W	RPL19B	1.27	1.23	0.92
YBR181C	RPS6B	1.27	1.09	0.98
YOL120C	RPL18A	1.27	0.81	1.07

YIL052C	RPL34B	1.26	1.31	0.81
YLR333C	RPS25B	1.25	0.83	0.97
YBR048W	RPS11B	1.24	1.05	1.30
YPL220W	RPL1A	1.23	1.06	1.10
YER102W	RPS8B	1.22	0.88	1.04
YMR116C	ASC1	1.22	1.02	1.58
YBR031W	RPL4A	1.21	1.05	1.36
YLR325C	RPL38	1.20	1.03	1.13
YER056C-/	RPL34A	1.20	1.35	0.86
YDL133C-/	RPL41B	1.19	1.34	1.17
YDR447C	RPS17B	1.16	1.80	0.99
YBR084C-/	RPL19A	1.16	1.08	0.90
YPR132W	RPS23B	1.16	0.86	0.91
YMR121C	RPL15B	1.16	0.74	0.80
YGR118W	RPS23A	1.15	0.72	0.84
YDR025W	RPS11A	1.15	1.04	1.06
YGR148C	RPL24B	1.15	1.42	0.82
YMR194W	RPL36A	1.12	0.66	0.85
YOL039W	RPP2A	1.11	0.88	0.95
YJL136C	RPS21B	1.11	1.11	0.94
YDR012W	RPL4B	1.02	1.11	1.31
YDL075W	RPL31A	1.01	0.92	0.82
YDL130W	RPP1B	1.00	0.81	0.99

a A clustergram representation of this data are shown in Fig. 5

	Average	1.46	1.02	1.09
	STDev	0.23	0.18	0.28
