# Supplementary material for: Genetic Interactions of MAF1 Identify a Role for Med20 in Transcriptional Repression of Ribosomal Protein Genes
Source: PLoS Genet. 2008 Jul 4;4(7):e1000112. doi: 10.1371/journal.pgen.1000112 (PMC2435279; doi:10.1371/journal.pgen.1000112)
Supplement: Text S1 — Supporting text. (0.10 MB PDF) [file pgen.1000112.s010.pdf]

Supporting Information

Genetic Interactions of *MAF1* Identify a Role for Mediator in Transcriptional Repression of Ribosomal Protein Genes.

Ian M. Willis, Gordon Chua, Amy H. Tong, Renee L. Brost, Timothy R. Hughes, Charles Boone and Robyn D. Moir

Comparison of microarray profiles for genes whose expression in the *med20Δ* strain is changed greater than or less than two fold relative to wild-type.

Processed data from van der Peppel et al., (1) corresponding to two biologically independent datasets for *med20Δ* (S288c background) in experiment E-UMCU-20 were downloaded from ArrayExpress (http://www.ebi.ac.uk/arrayexpress/#ae-main[0]). Intensity ratios from mean data normalized on genes were averaged for the duplicate spots in each experiment. The data was then filtered to extract genes that showed effects greater than or less than two fold in both experiments. This yielded 42 genes whose expression decreased and 17 genes whose expression increased in the *med20Δ* strain. Applying the same criteria to our *med20Δ* versus wild-type microarray data yielded 75 genes whose expression decreased and 41 genes whose expression increased in the *med20Δ* strain. Given the relatively small number of genes affected in both studies and the limited overlap (only 11 genes decreased and 2 genes increased, respectively), we merged the sets of affected genes in both studies and analyzed the combined data for shared GO-Bioprocess terms using SGD GO-Slim Mapper. The results are compiled in Table S3.

Comparison of microarray profiles for genes whose expression in a wild-type strain is changed greater than or less than two fold in response to rapamycin treatment.

Two datasets (GSM70804 and GSM70805) reported by Chen and Powers (2) that examined the effect of rapamycin on gene expression in strain S288C growing in YPD medium were downloaded from the GEO database (http://www.ncbi.nlm.nih.gov/geo). Expression ratios were averaged and filtered to extract genes that showed effects greater than or less than two fold. This yielded 679 genes whose expression decreased and 642 genes whose expression increased following a 60 min. treatment with rapamycin. Using the same microarray platform (spotted ORFs) and the same strain (S288c), growth conditions (YPD medium) and time of rapamycin treatment (1 hour), we identified a similarly large group of genes that were repressed (523 genes) or activated (502 genes) two fold or more. These two groups of genes showed substantial overlap (Fig. S3) with the corresponding data from Chen and Powers (2). Among the genes whose expression changed 2 fold or more in our experiments were 113 out of 138 RP genes and 127 out of 236 *Ribi* genes from the repressed gene set (3) and 125 out of 490 Gcn4 target genes from the induced gene set (4). As found in other microarray studies, rapamycin treatment activates only a subset of Gcn4 target genes (2,4).

References

1. van de Peppel, J. *et al.* Mediator expression profiling epistasis reveals a signal transduction pathway with antagonistic submodules and highly specific downstream targets. *Mol.Cell* **19**, 511-522 (2005).

2. Chen, J. C. & Powers, T. Coordinate regulation of multiple and distinct biosynthetic pathways by TOR and PKA kinases in S. cerevisiae. *Curr.Genet.* **49**, 281-293 (2006).

3. Jorgensen, P. *et al.* A dynamic transcriptional network communicates growth potential to ribosome synthesis and critical cell size. *Genes Dev.* **18**, 2491-2505 (2004).

4. Hardwick, J. S., Kuruvilla, F. G., Tong, J. K., Shamji, A. F., & Schreiber, S. L. Rapamycin-modulated transcription defines the subset of nutrient-sensitive signaling pathways directly controlled by the Tor proteins. *Proc.Natl.Acad.Sci.U.S.A* **96**, 14866-14870 (1999).

Supporting Information Figure Legends

Fig. S1. Northern analysis of RP genes in wild-type and *MAF1* SSL strains before and after rapamycin treatment. A Wild-type and deletion strains were grown in YPD and treated with rapamycin for 1 hour as for the microarray experiments (see Materials and Methods). RNA was extracted and Northern analysis was performed on formaldehyde-agarose gels using ^{32}P-labeled *RPL3*, *RPL28* and U3 snRNA oligonucelotide probes (Upadhya et al. 2002 *Mol Cell* **10**, 1489-1494). Of the 35 non-essential *MAF1* SSL strains (Table S1) only the six shown in the figure were significantly compromised for repression of RP genes. **B** For each strain in panel A, *RPL* bands quantified by phosphorimage analysis were normalized to U3 snRNA and the extent of repression by rapamycin was determined relative to the untreated control. This value was set to 1 for the wild-type strain for comparison with the different mutant strains. **C** Phosphorimage quantitation of signal intensities from multiple biologically independent samples (wild-type n = 4 and *med20Δ* n = 3) were used to calculate the average level of repression, normalized for U3 snRNA. Relative to the untreated controls, the level of repression was reduced from 7 ± 4% in the wild-type strain to 18 ±

1% in the *med20Δ* strain for *RPL3* and from 7 ± 2% in the wild-type strain to 36 ± 10% in the *med20Δ* strain for *RPL28.*

Fig. S2. Transcription of a tRNA^{Leu} gene is robustly repressed by rapamycin in the *med20Δ* strain. A Wild-type and mutant strains in the W303 background were grown at 30°C to prepare total RNA for Northern analysis (Upadhya et al. 2002 *Mol Cell* **10**, 1489-1494). The blot was probed for a short-lived precursor tRNA (pre-tRNA^{Leu}) and for U3 snRNA, which serves as a loading control. **B** Growth phenotypes of the strains shown in panel A and two independent *maf1Δ med20Δ* isolates are compared on YPD medium at 30° and 37°C. Ten-fold serial dilutions were spotted after normalization for cell density. The synthetic phenotype of the double mutant strain is evident at both temperatures.

Fig. S3. Microarray analysis of genes induced and repressed by rapamycin treatment. Microarray datasets GSM70804 and GSM70805 from http://www.ncbi.nlm.nih.gov/geo (Chen and Powers 2006, *Curr.Genet.* **49**, 281-293) and from the current study were analyzed as described in the Supporting Information Text. The Venn diagram displays the overlap between the data from the two studies. The average of the total number of genes affected (2 fold or more) in both studies shows that rapamycin changes the expression profile of ~20% of the genome.

Fig. S4. Clustergram comparison of *med20Δ* versus wild-type expression ratios under different environmental conditions. Microarrays were hybridized to compare expression patterns under six conditions as indicated below the figure. Except for transient heat stress, RNA samples were prepared from cells growing at 30°. Details of

the repressing conditions are given in the Materials and Methods. Decreased (green) and increased (red) expression is shown relative to the wild-type strain. The expression of 1063 genes that increased or decrease by two fold or more in any one of the six comparisons were subjected to two dimensional hierarchical clustering.
